# Integrative taxonomy, biogeography and conservation of freshwater mussels (Unionidae) in Russia

**DOI:** 10.1038/s41598-020-59867-7

**Published:** 2020-02-20

**Authors:** Ivan N. Bolotov, Alexander V. Kondakov, Ekaterina S. Konopleva, Ilya V. Vikhrev, Olga V. Aksenova, Andrey S. Aksenov, Yulia V. Bespalaya, Alexey V. Borovskoy, Petr P. Danilov, Gennady A. Dvoryankin, Mikhail Y. Gofarov, Mikhail B. Kabakov, Olga K. Klishko, Yulia S. Kolosova, Artem A. Lyubas, Alexander P. Novoselov, Dmitry M. Palatov, Grigory N. Savvinov, Nikolay M. Solomonov, Vitaly M. Spitsyn, Svetlana E. Sokolova, Alena A. Tomilova, Elsa Froufe, Arthur E. Bogan, Manuel Lopes-Lima, Alexander A. Makhrov, Maxim V. Vinarski

**Affiliations:** 1 Northern Arctic Federal University, Arkhangelsk, Russia; 2 Federal Center for Integrated Arctic Research, Russian Academy of Sciences, Arkhangelsk, Russia; 3 Laboratory of Macroecology & Biogeography of Invertebrates, Saint Petersburg State University, Saint Petersburg, Russia; 4 Scientific Research Institute of Applied Ecology of the North, M. K. Ammosov North-Eastern Federal University, Yakutsk, Republic of Sakha (Yakutia) Russia; 5 Institute of Natural Resources, Ecology and Cryology, Siberian Branch of the Russian Academy of Sciences, Chita, Russia; 6 A. N. Severtzov Institute of Ecology and Evolution, Russian Academy of Sciences, Moscow, Russia; 7 Lomonosov Moscow State University, Moscow, Russia; 8 CIIMAR/CIMAR – Interdisciplinary Centre of Marine and Environmental Research, University of Porto, Terminal de Cruzeiros do Porto de Leixões, Matosinhos, Portugal; 9 Research Laboratory, North Carolina Museum of Natural Sciences, Raleigh, North Carolina United States of America; 10 CIBIO/InBIO – Research Center in Biodiversity and Genetic Resources, University of Porto, Campus Agrário de Vairão, Vairão, Portugal; 11 SSC/IUCN – Mollusc Specialist Group, Species Survival Commission, International Union for Conservation of Nature, Cambridge, United Kingdom

**Keywords:** Biodiversity, Biogeography, Zoology

## Abstract

Freshwater mussels are ecosystem engineers and keystone species in aquatic environments. Unfortunately, due to dramatic declines this fauna is among the most threatened globally. Here, we clarify the taxonomy and biogeography of Russian Unionidae species based on the most comprehensive multi-locus dataset sampled to date. We revise the distribution and assess the conservation status for each species. This fauna comprises 16 native species from 11 genera and 4 tribes: *Anodonta*, *Pseudanodonta* (Anodontini); *Amuranodonta*, *Beringiana*, *Buldowskia*, *Cristaria*, *Sinanodonta* (Cristariini); *Middendorffinaia*, *Nodularia*, *Unio* (Unionini); and *Lanceolaria* (Lanceolariini). No country-level endemic species are known in Russia, except for *Buldowskia suifunica* that may also occur in China. *Sinanodonta woodiana*, a non-native species, was introduced from China. Russia comprises the northern parts of Western and Eastern Palearctic subregions. The first subregion with six species encompasses a huge area from the western boundary of Russia to the Lena Basin in Siberia. The second subregion with 10 species covers the Amur Basin, rivers east of the Lena Basin, coastal basins of the Japan Sea, and the North Pacific Islands. The fauna of Russia primarily includes widespread generalist species that are here considered Least Concern (LC). However, *Buldowskia suifunica* and *Sinanodonta lauta* have restricted distributions and are assessed here as Vulnerable (VU) and Endangered (EN), respectively.

## Introduction

Freshwater mussels (order Unionida) are ecologically and economically important aquatic animals^1^ that are sensitive to water pollution, habitat loss, climate changes, and other negative anthropogenic and natural impacts^2–4^. These animals are widely distributed throughout Russia, representing keystone taxa in various water bodies of European Russia, Siberia, and the Russian Far East (mainland, Sakhalin, and Kurile Archipelago)^5^. However, freshwater mussels are not known to occur in the Polar Urals, Yamal and Taymyr peninsulas, Arctic Ocean Islands, and several mountain rivers (e.g. headwaters and middle reaches of the Amgun River, a tributary of the Amur River)^6,7^. Furthermore, several large, hard-to-reach river basins in Eastern Siberia and the Far East are still to be explored. In general, the freshwater mussel fauna in Russia largely reflects natural biogeographic and environmental patterns^8^, but a human footprint can also be traced in a few non-native populations of *Unio* and *Sinanodonta* species discovered in Siberia^9,10^.

The study of freshwater mussel systematics in Russia has a long history. The earliest taxonomic works on the unionid fauna of the Russian Empire and its regions appeared in the middle of the 19th century^11–16^. A few decades later, Alexander Buldowski developed a research project on economically important freshwater mussels of the Russian Far East^17^. Prof. Vladimir Zhadin published two large monographs with comprehensive reviews of the taxonomy, distribution, biology and ecology of freshwater mussels throughout the USSR^5,6^. In the early 1970s, Dr. Iya Moskvicheva presented three papers revising the taxonomy of the Unionidae from the Russian Far East, with supplementary data on freshwater mussels from Mongolia, Korea, Japan, and China^7,18,19^. During the next 40 years, Prof. Yaroslav Starobogatov and his disciples published a plethora of taxonomic works and identification guides on freshwater mussels from Russia, with a special focus on the Russian Far East and Siberia^20–24^. A thorough review of the body of historical literature is presented in the recent catalogue of molluscs from fresh and brackish water bodies of ex-USSR^25^.

However, all of these historical works were based solely on a morphological approach that has biased the taxonomic solutions due to the high variability of the shell shape, convexity and anatomical features in freshwater mussels^26–31^. While the taxonomy and distributional patterns of the Russian Margaritiferidae species have been clarified in detail using an integrative approach combining molecular, morphological and biogeographic evidences^27–29,32,33^, those of the family Unionidae, remain largely unclear^25^.

Graf^26^ provided the first critical taxonomic revision of freshwater mussels from the Northern Palearctic Region based on morphological features. Several local integrative revisions on Russian Unionidae taxa have been published, i.e. works on the taxonomy of the genera *Cristaria*^34,35^, *Sinanodonta*^36,37^, *Anodonta*^38^, *Unio*^9^, *Nodularia*^39^, and *Middendorffinaia*^40^. Lopes-Lima *et al*.^41^ revisited the tribal and generic clades within Unionidae using a global multi-locus phylogeny. A broad-scale review of freshwater mussels in Europe includes important information from European Russia^42^. Zieritz *et al*.^43^ compiled a useful summary of the recent knowledge on freshwater mussels of Asia. Recently, Lopes-Lima *et al*.^44^ prepared a comprehensive overview of freshwater mussels of East Asia with a description of several new taxa. However, a revision of the Unionidae in Russia is far from being complete, with multiple taxa having a doubtful taxonomic status, especially those from Siberia and the Far East.

Considering the issues outlined above, this study aims to provide an integrative revision of the Russian Unionidae based on the most comprehensive molecular data set sampled to date. We clarify the actual taxonomic richness of this family in Russia and describe the distribution patterns for each genus and species. Using the distribution data and our multi-locus phylogeny, we propose an updated biogeographic division for Unionidae of Russia and briefly discuss their species richness in each biogeographic region and province. Finally, we assess the conservation status of every valid species-level lineage and propose the national-level priorities for further freshwater mussel research in Russia.

## Results

### Multi-locus phylogeny and species richness of the Russian Unionidae

**Figure 1:**
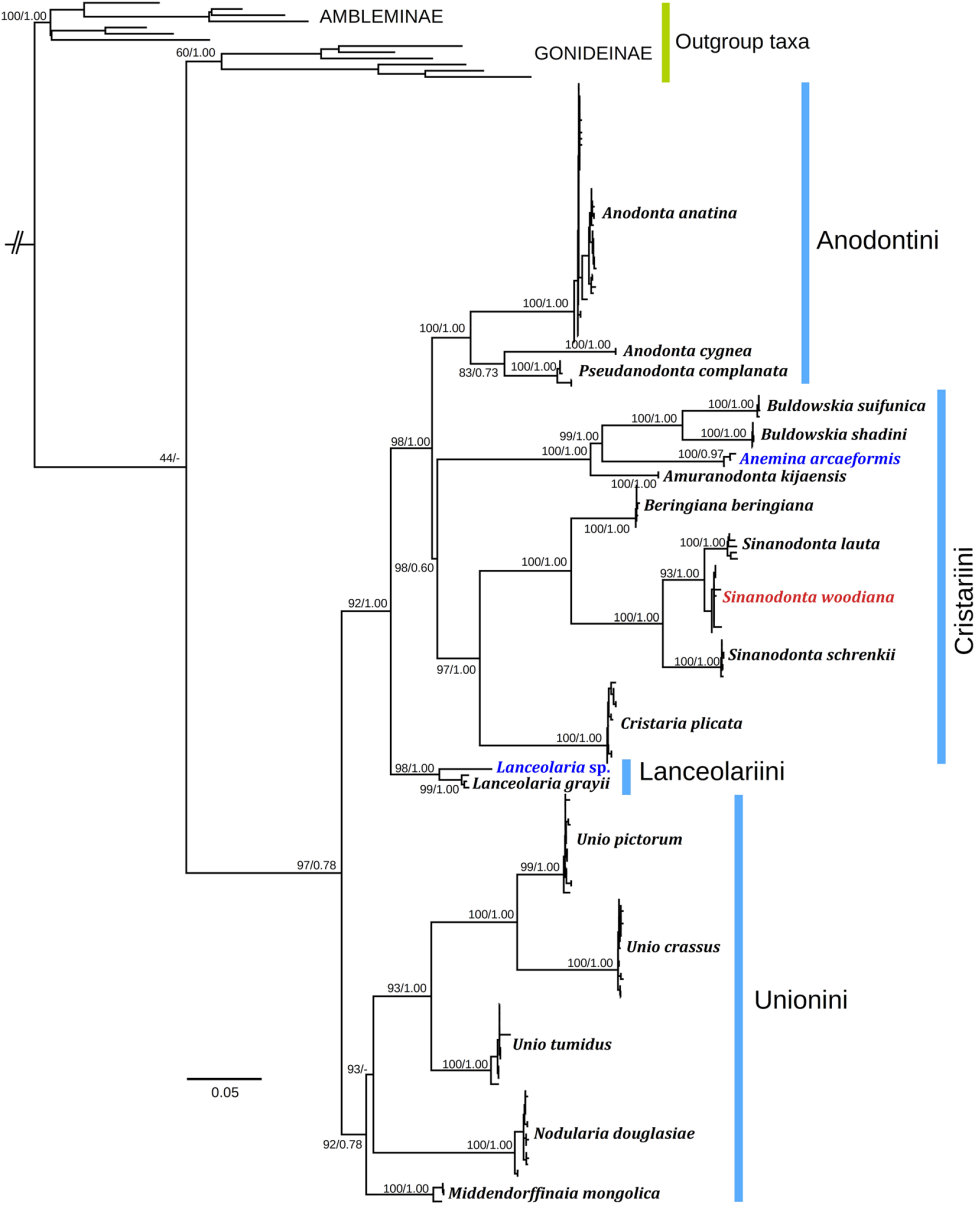
Maximum likelihood (IQ-TREE) phylogeny of freshwater mussels (Unionidae) from Russia and adjacent countries (five partitions: three codons of *COI* + *16S rRNA* + *28S rRNA*). Numbers near nodes are BS/BPP values. Red label indicates a non-native species; blue labels indicate taxa from outside Russia. The list of sequences is presented in Supplementary Table 1.

**Figure 2:**
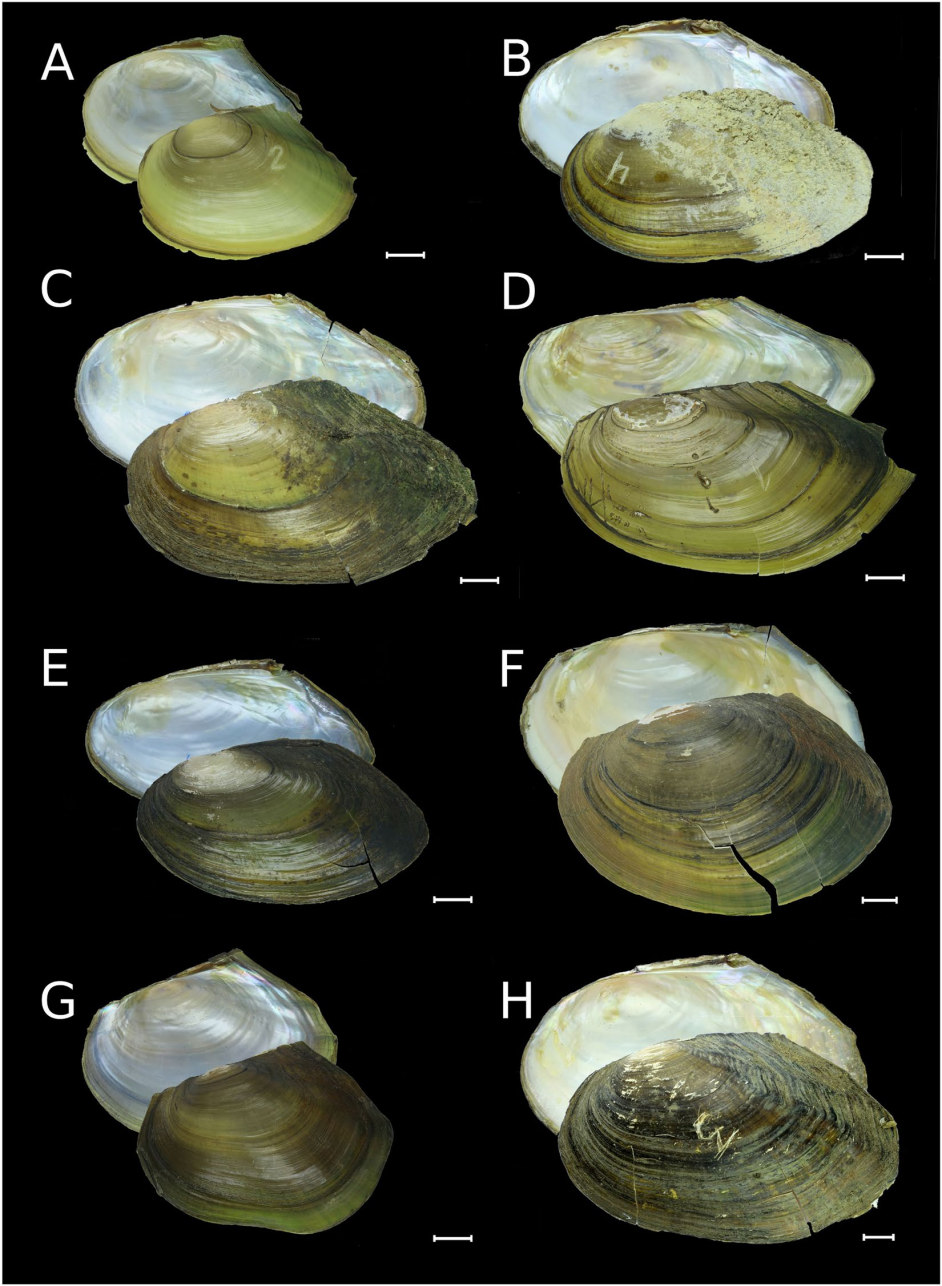
Shells of *Anodonta*, *Pseudanodonta* and *Sinanodonta* from Russia. (**A**) *Anodonta anatina*, Lake Syrdah, Lena River basin, Eastern Siberia [RMBH biv199_2]. (**B**) *Anodonta anatina*, Lake Gusinoye, Yenisei River basin, Eastern Siberia [RMBH biv190_4]. (**C**) *Anodonta anatina*, Oka River, Volga River basin, European Russia [RMBH biv573_4]. (**D**) *Anodonta cygnea*, Medvezhii Lakes, Moscow, European Russia [RMBH biv194_1]. (**E**) *Pseudanodonta complanata*, Khopyor River, Don River basin, European Russia [RMBH 195_1]. (**F**) *Sinanodonta lauta* [=*S. ovata* Bogatov & Starobogatov, 1996, a topotype], Gladkaya River, Russian Far East [RMBH biv225_2]. (**G**) *Sinanodonta schrenkii*, Melgunovka River, Khanka Lake basin, Russian Far East [RMBH biv496_3]. (**H**) *Sinanodonta woodiana*, non-native population, Yenisei River near Krasnoyarsk, Eastern Siberia [RMBH biv191_3]. Scale bars = 10 mm. (Photos: Ekaterina S. Konopleva [**A**,**B**,**D**–**G**] and Olga V. Aksenova [**C**,**H**]).

**Figure 3:**
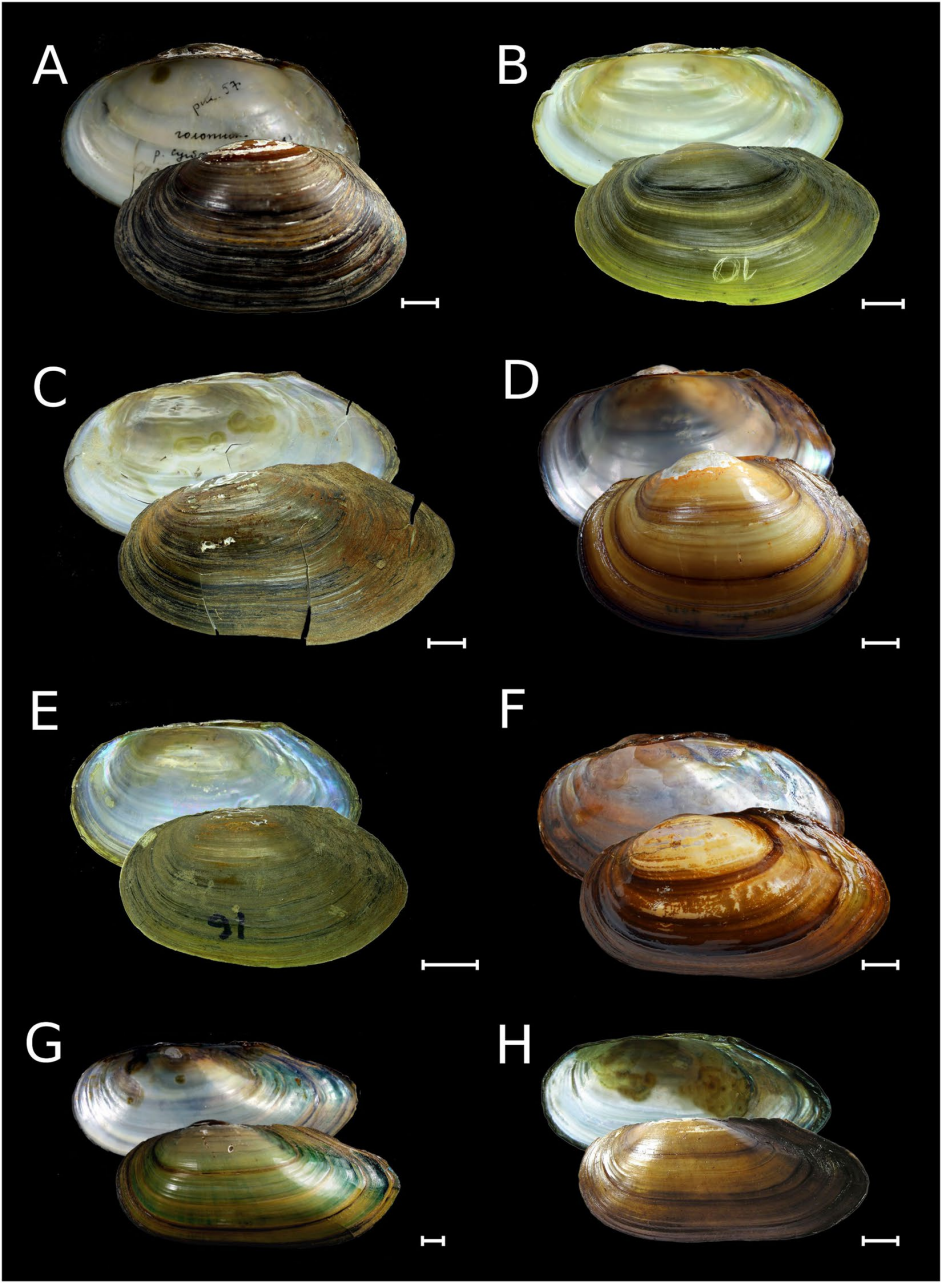
Shells of *Buldowskia* and *Amuranodonta* from Russia. (**A**) *Buldowskia suifunica*, holotype, Razdolnaya River near Ussuriysk city, Russian Far East [ZISP]. (**B**) *Buldowskia suifunica*, a topotype, Lake Soldatskoye near Ussuriysk city, Razdolnaya Basin, Russian Far East [RMBH biv227_10]. (**C**) *Buldowskia suifunica* [=*Buldowskia koreana* Bogatov & Starobogatov, 1996, a topotype], Gladkaya River, Russian Far East [RMBH biv225_11]. (**D**) *Buldowskia shadini*, holotype, Mandzhurka (Novo-Troitskaya) River, Khanka Lake basin, Russian Far East [ZISP]. (**E**) *Buldowskia shadini*, Lake Blagodatnoye, Ussuri Basin, Russian Far East [RMBH 228_16]. (**F**) *Buldowskia shadini*, Onon River, Amur Basin, Transbaikalia [INREC]. (**G**) *Amuranodonta kijaensis*, holotype, Zarechnoye Lake, near Polyotnoye Settlement, Kiya River basin, Amur River drainage, Russian Far East [ZISP]. (**H**) *Amuranodonta kijaensis*, Arey Lake, Transbaikalia [INREC]. Scale bars = 10 mm. (Photos: Maxim V. Vinarski [**A**,**D**,**G**], Ekaterina S. Konopleva [**B**,**E**], Olga V. Aksenova [**C**], and Olga K. Klishko [**F**,**H**]).

**Figure 4:**
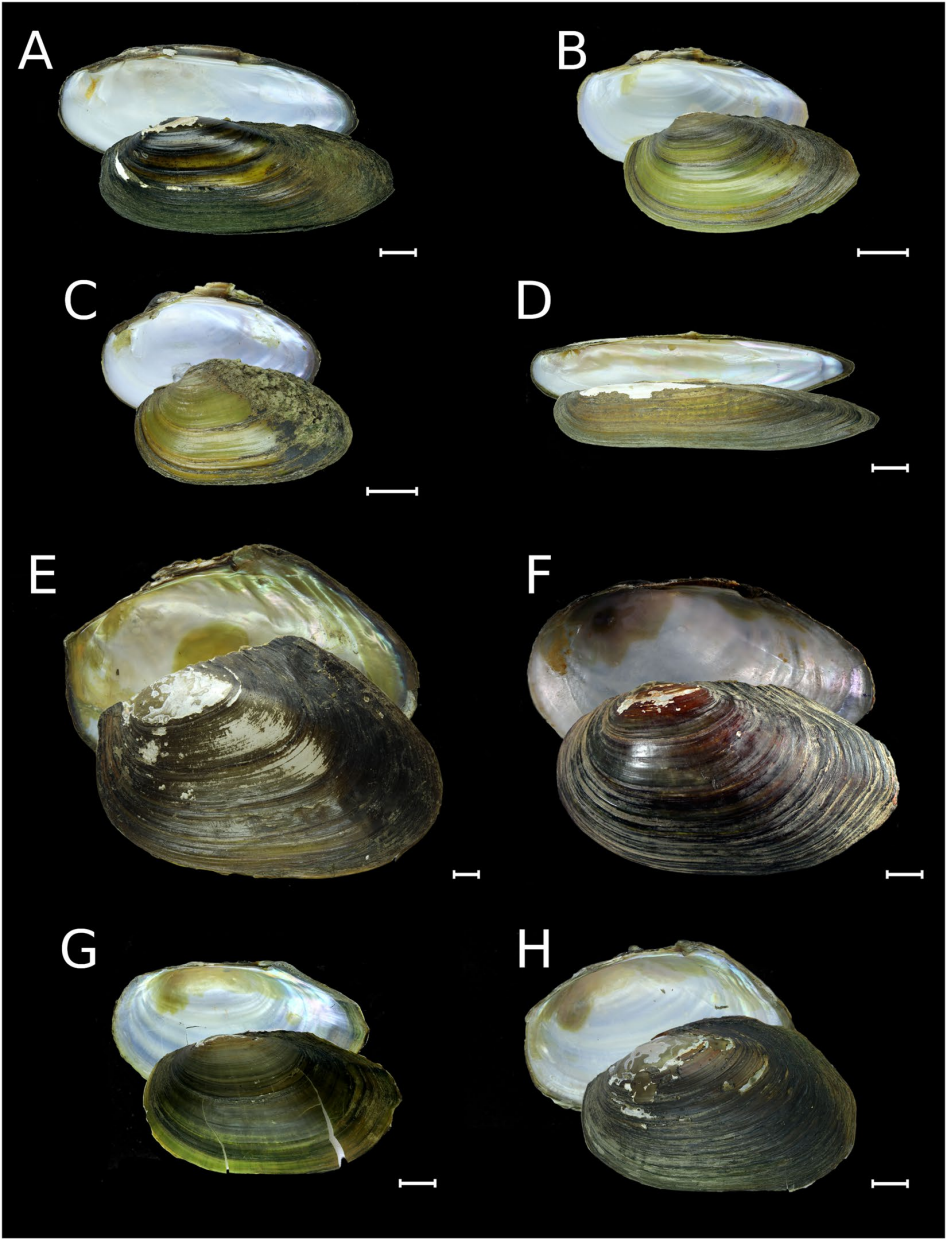
Shells of *Unio*, *Lanceolaria*, *Cristaria*, and *Beringiana* from Russia and the United States of America. (**A**) *Unio pictorum*, Khopyor River, Don River basin, European Russia [RMBH biv282_1]. (**B**) *Unio tumidus*, Lake Lacha, Onega River basin, European Russia [RMBH 568_2]. (**C**) *Unio crassus*, Iren River, Volga River basin, European Russia [RMBH 304_8]. (**D**) *Lanceolaria grayii*, Lake Khanka, Russian Far East [RMBH biv502_2]. (**E**) *Cristaria plicata*, Lake Khanka, Russian Far East [RMBH biv495_27]. (**F**) *Beringiana beringiana*, holotype, Kenai Lake, Unalashka Island, Aleutian Islands, USA [ZISP]. (**G**) *Beringiana beringiana* [=*Arsenievinaia alimovi* Bogatov & Zatravkin, 1988, topotype], Avakumovka River, Russian Far East [RMBH biv272]. (**H**) *Beringiana beringiana* [=*Arsenievinaia coptzevi* Zatravkin & Bogatov, 1987, a topotype], Lake Vas’kovskoye near Rudnaya Pristan village, Russian Far East [RMBH biv273_2]. Scale bars = 10 mm. (Photos: Ekaterina S. Konopleva [**A**–**E**, **G**–**H**] and Maxim V. Vinarski [**F**]).

**Figure 5:**
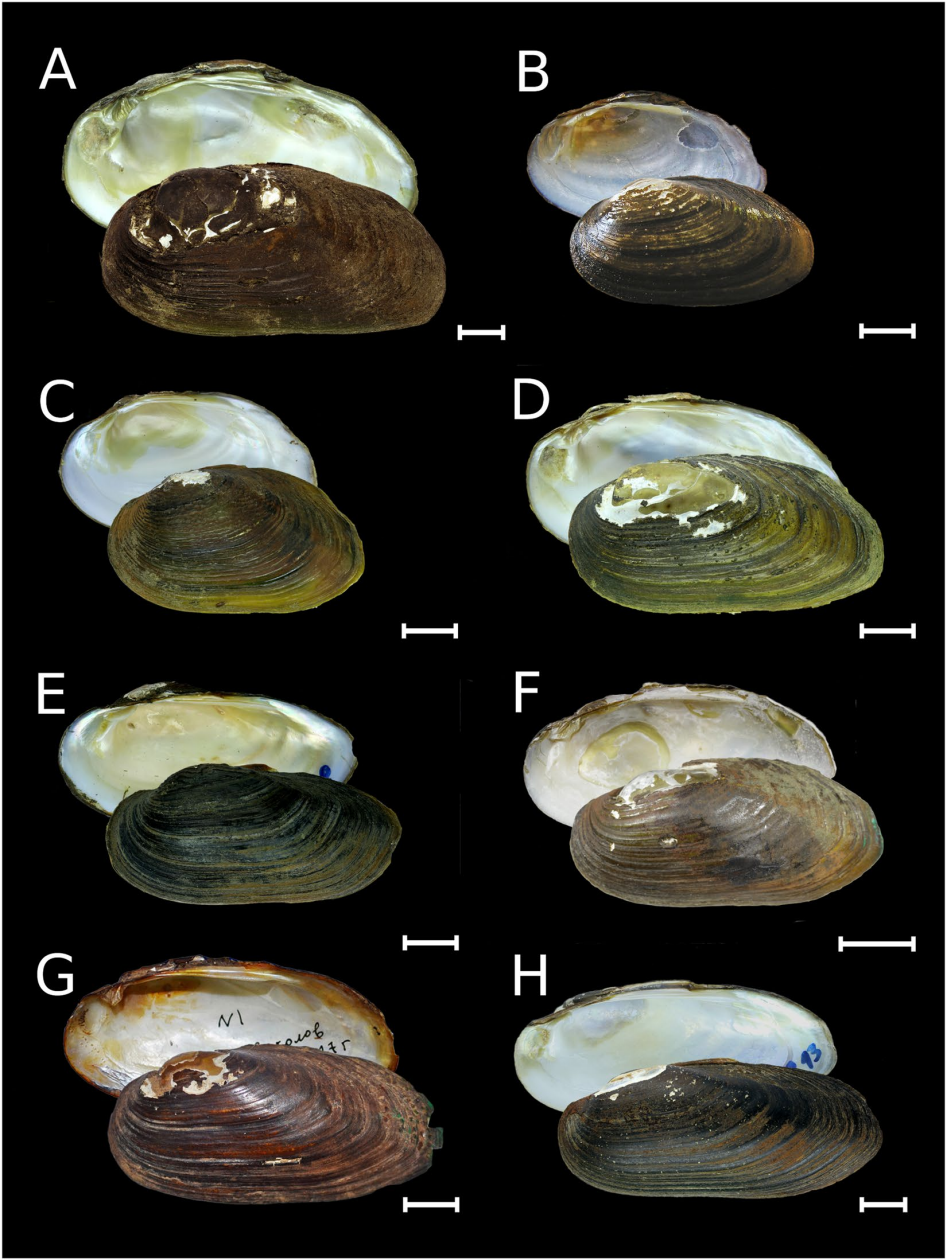
Shells of *Middendorffinaia mongolica* and *Nodularia douglasiae* from Russia. (**A**) *M. mongolica*, Gladkaya River, Russian Far East [neotype RMBH biv229_5]. (**B**) *M. mongolica*, Shilka River, Transbaikalia [INREC]. (**C**) *M. mongolica*, young sculptured shell, Gladkaya River, Russian Far East [RMBH biv229_7]. (**D**) *M. mongolica* [=*M. dulkeitiana* Moskvicheva & Starobogatov, 1973, a topotype], a tributary of Komarovka [Suputinka] River, Razdolnaya Basin, Russian Far East [RMBH 99_3]. (**E**) *N. douglasiae*, Lake Soldatskoye near Ussuriysk city, Razdolnaya Basin, Russian Far East [RMBH biv227_12]. (**F**) *N. douglasiae* [=*Middendorffinaia ochotica* Bogatov, 2000, holotype], Kukhtui River northward of Okhotsk, Russian Far East [ZISP]. (**G**) *N. douglasiae* [=*Middendorffinaia mongolica* sensu Moskvicheva & Starobogatov, 1973 non Middendorff, 1851, a specimen selected by Moskvicheva and Starobogatov^18^ as a representative of Middendorff’s taxon], Arsenievka River near Yakovlevka village, Lake Khanka basin, Russian Far East [ZISP]. (**H**) *N. douglasiae* [=*Unio pictorum* var. *amurensis* Mousson, 1887, a topotype], Amur River near Nikolaevsk-on-Amur, Russian Far East [RMBH biv134_13]. Scale bars = 10 mm. (Photos: Ekaterina S. Konopleva [**A**,**C**,**D**,**E**,**H**], Olga K. Klishko [**B**,**G**] and Ilya V. Vikhrev [**F**]).

Based on our novel multi-locus phylogeny (Fig. 1) and morphological data, we found that the Russian Unionidae fauna includes 16 native species from 11 genera and 4 tribes: *Anodonta*, *Pseudanodonta* (Anodontini), *Amuranodonta*, *Beringiana*, *Buldowskia*, *Cristaria*, *Sinanodonta* (Cristariini), *Middendorffinaia*, *Nodularia*, *Unio* (Unionini), and *Lanceolaria* (Lanceolariini) (Table 1 and Figs. 2–5). Additionally, the non-native species *Sinanodonta woodiana* was introduced from China. General information on each genus is given in Taxonomic Account. A modern taxonomic concept for every valid biological species in Russia is established in the Supplementary Note. The genus *Anemina* in its current understanding includes three highly divergent subclades (Fig. 1) that are here considered separate genera: *Anemina* s. str. (occurs in China, South Korea, and Japan but not in Russia), *Amuranodonta* (Amur Basin in Russia and China), and *Buldowskia* (Russian Far East, Korea, and Japan). Typical habitats of the Russian Unionidae are illustrated in Supplementary Figs. 1 and 2.

**Table 1:** Checklist of freshwater mussels (Unionidae) in Russia (full synonymy for each species is presented in Supplementary Note).

Tribe	Genus	Species	Type locality	Distribution range	Number of synonyms introduced since 1970s
Anodontini Rafinesque, 1820	*Anodonta* Lamarck, 1799	*A. anatina* (Linnaeus, 1758)	Europe, fresh water	European Russia, Urals and Siberia up to the Lena River basin; Kazakhstan; countries of Northern, Eastern and Western Europe^42^; Selenga River basin in Mongolia^113^; introduced in a warm channel of the Kola Nuclear Power Plant, Kola Peninsula^68^	6
		*A. cygnea* (Linnaeus, 1758)	Europe, mouths of rivers	European Russia (water bodies of the Baltic, Black, Azov, and Caspian Sea drainages); countries of Northern, Eastern and Western Europe^42^	0
	*Pseudanodonta* Bourguignat, 1876	*P. complanata* (Rossmässler, 1835)	Germany, Elbe River	European Russia (water bodies of the Baltic, Black, Azov, and Caspian Sea drainages); countries of Northern, Eastern and Western Europe^42^	1
Cristariini Lopes-Lima *et al*., 2017	*Amuranodonta* Moskvicheva, 1973	*A. kijaensis* Moskvicheva, 1973	Russia, Khabarovsk Region, Kiya River basin, near Polyotnoye Settlement, Zarechnoye Lake	Amur Basin and Lake Arey in Transbaikalia; Ussuri River in northeastern China^47^; putative endemic lineage to the Amur Basin in Russia and China	6
	*Beringiana* Starobogatov in Zatravkin, 1983	*B. beringiana* (Middendorff, 1851)	USA, Aleutian Islands, Unalashka Island, Kenai Lake	Widespread from Kievka River (northeast of Vladivostok) to Kamchatka and from Indigirka River to Chukotka, Kurile Archipelago and Sakhalin; Japan; Alaska, Western Pacific Region, and Canada in North America^44,49^	16
	*Buldowskia* Moskvicheva, 1973	*B. suifunica* (Lindholm, 1925)	Russia, Primorye Region, Razdolnaya River near Ussuriysk city	Razdolnaya River basin and coastal rivers southwest of Vladivostok; tentative local endemic lineage to the Russian Far East, but can be found in North Korea and northeastern China	7
		*B. shadini* (Moskvicheva, 1973)	Russia, Primorye Region, Khanka Lake basin, Mandzhurka (Novo-Troitskaya) River	Amur Basin in Russia, northeastern China and Mongolia (Lake Buir) to South Korea^44,50^	4
	*Cristaria* Schumacher, 1817	*C. plicata* (Leach, 1814)	“A Bohemian river” [erroneous; probably China]	Amur Basin in Russia, Mongolia and northeastern China; one record from Tym’ River, central Sakhalin; Korea, Japan, eastern China (Yangtze Basin), and northern Vietnam;^34,35,44,51,53,70^ few records from the Mekong Basin (probably a non-native population)^114,115^	0
	*Sinanodonta* Modell, 1945	*S. lauta* (Martens, 1877)	Japan, Tokyo, Ueno Park, Shinobazu Pond	Native to coastal rivers southwest of Vladivostok, Japan and Korea;^44,50^ introduced in the Yenisei River, Eastern Siberia^10^	2
		*S. schrenkii* (Lea, 1870)	Amur River	Amur and Razdolnaya basins in Russia; Halhin River in Mongolia (a tributary of Lake Buir, Amur Basin), and South Korea^44,50^	6
		*S. woodiana (Lea, 1834)	China	Native to the Yangtze Basin^37^; introduced in the Yenisei River, Eastern Siberia^10^, many European countries^116^, Uzbekistan^37^, and Myanmar^117^	N/A
Lanceolariini Froufe *et al*., 2017	*Lanceolaria* Conrad, 1853	*L. grayii* (Griffith & Pidgeon, 1833)	Not indicated	Khanka Lake, Ussuri Basin and Lower Amur in Russia; Yangtze Basin in China^44,53^	4
Unionini Rafinesque, 1820	*Middendorffinaia* Moskvicheva & Starobogatov, 1973	*M. mongolica* (Middendorff, 1851)	Russia, Primorye Region, downstream of Gladkaya River (determined by the neotype)	Amur and Razdolnaya basins, coastal rivers of the Japan Sea drainage west of Nakhodka (Partizanskaya and Artemovka rivers)^54^ and southwest of Vladivostok; Onon and Argun basins in Mongolia^113^; putative endemic lineage to the Russian Far East and Mongolia, but can be found in North Korea and northeastern China	8
	*Nodularia* Conrad, 1853	*N. douglasiae* (Griffith & Pidgeon, 1833)	Not indicated	Amur and Razdolnaya basins, coastal rivers of the Okhotsk Sea drainage basin up to the Ola River just north of Magadan^55,58^, northwestern Sakhalin^71^; Lake Buir (Amur Basin) in Mongolia and northeastern China^118^, eastern China (Yangtze Basin), Korea, Japan, and northern Vietnam^39,53,87^	9
	*Unio* Retzius, 1788	*U. pictorum* (Linnaeus, 1758)	European rivers	European Russia, western Urals, countries of Northern, Eastern and Western Europe^42^; introduced in Lake Kenon, Amur Basin, Transbaikalia^9^	1
		*U. tumidus* Retzius, 1788	European rivers	European Russia, western Urals; Ural River in Russia and Kazakhstan; Irtysh Basin in Western Siberia^59^ and Kazakhstan; historical records of recent shells from Ob’ River in Western Siberia^5^; countries of Northern, Eastern and Western Europe^42^; introduced in Lake Kenon^9^ and the Upper Amur Basin in Transbaikalia	0
		*U. crassus* Retzius, 1788	European rivers	European Russia (water bodies of Baltic, Black, Azov, and Caspian Sea drainage basins), western Urals; Ural River in Russia and Kazakhstan; countries of Northern, Eastern and Western Europe^42^	0

*Non-native species. N/A – not available.

### Biogeographic patterns

**Figure 6:**
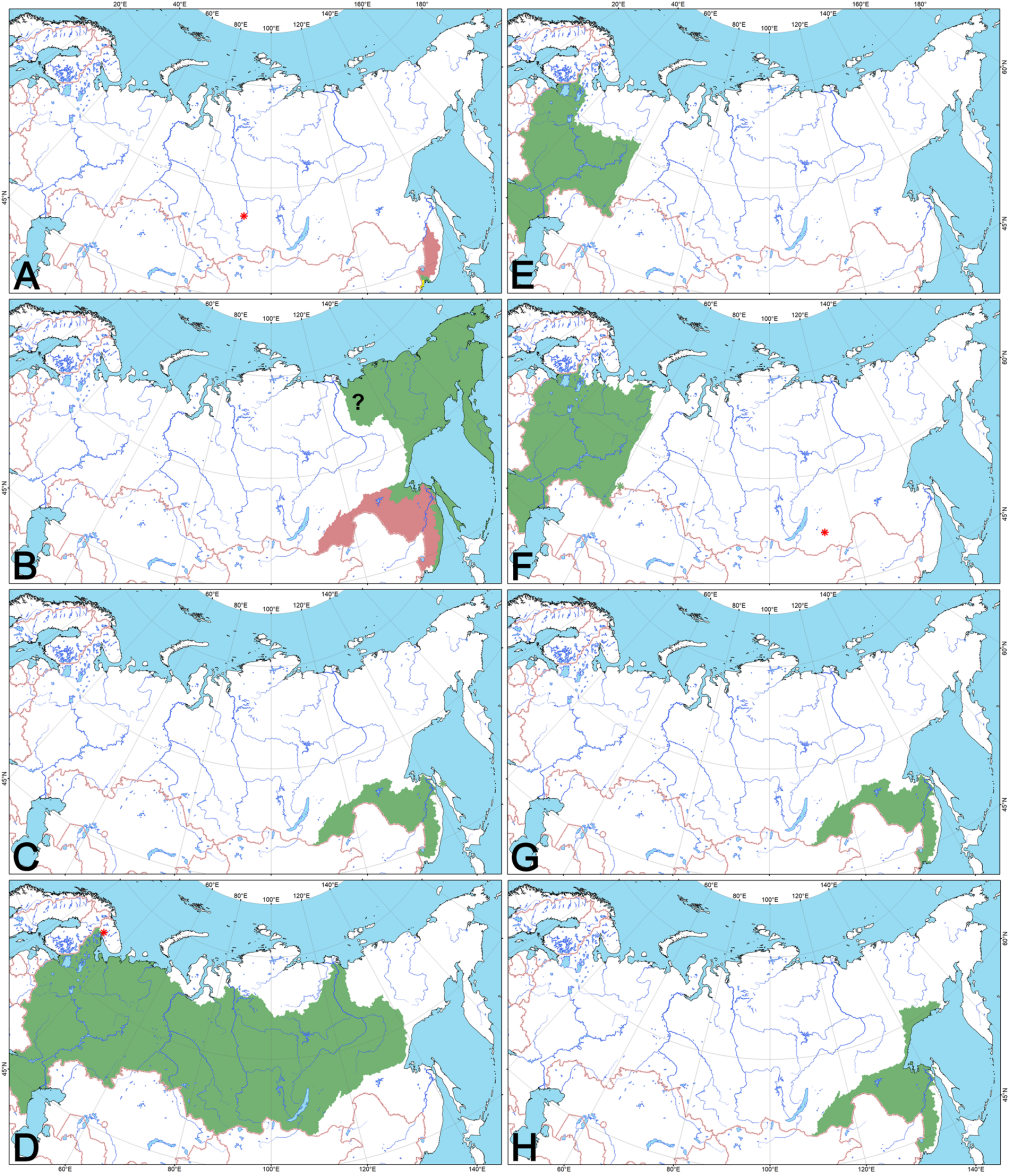
Distribution of freshwater mussel species in Russia. (**A**) *Buldowskia suifunica* (green and yellow fillings), *Sinanodonta lauta* (yellow filling), and *Lanceolaria grayii* (red filling); non-native populations of *Sinanodonta woodiana* and *S. lauta* in Yenisei River (red asterisk)^10^. (**B**) *Beringiana beringiana* (green filling; the question mark indicates the Yana River, certain records from which are absent) and *Sinanodonta schrenkii* (red filling). (**C**) *Amuranodonta kijaensis*, *Buldowskia shadini*, and *Cristaria plicata* (green filling); a native population of *Cristaria plicata* in Sakhalin (green asterisk). (**D**) *Anodonta anatina* (green filling), and its non-native population in a warm channel of the Kola Nuclear Power Plant, Kola Peninsula^68^ (red asterisk). (**E**) *Anodonta cygnea*, *Pseudanodonta complanata*, and *Unio crassus* (green filling). (**F**) *Unio pictorum* and *U. tumidus* (green filling); a local native population of *U. tumidus* in the Irtysh Basin^59^ (green asterisk), and non-native populations of *U. pictorum* and *U. tumidus* in Lake Kenon, Amur Basin^9^ (red asterisk). (**G**) *Middendorffinaia mongolica* (green filling). (**H**) *Nodularia douglasiae* (green filling). The topographic base of the maps was compiled with Natural Earth Free Vector and Raster Map Data (www.naturalearthdata.com), and the HydroSHEDS database (www.hydrosheds.org)^119,120^. (Maps: Mikhail Y. Gofarov).

**Figure 7:**
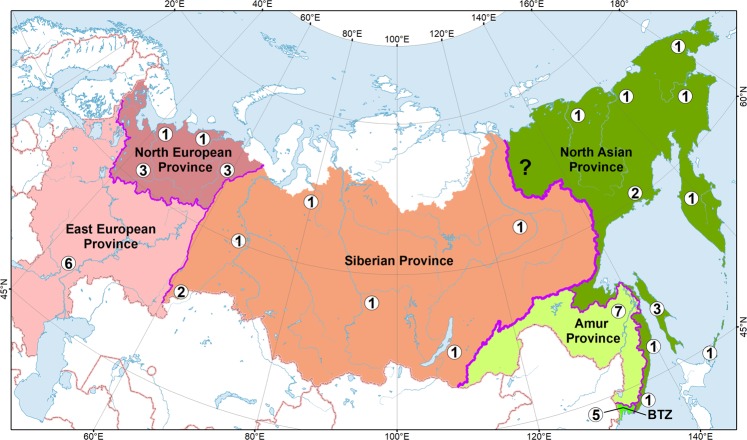
Biogeographic division of Russia based on the distribution patterns in freshwater mussels (Unionidae). The numbers in white circles indicate the Unionidae species richness in corresponding freshwater systems. The question mark indicates the Yana River, certain records from which are absent. The thick pink line indicates the boundary between the Western Palearctic (in red colors) and Eastern Palearctic (in green colors) subregions. The narrower pink lines indicate boundaries between biogeographic provinces. BTZ = Amur–Korean–Japanese Biogeographic Transition Zone. This biogeographic scheme is based on the results of PCA analysis (Supplementary Fig. 3) of the comprehensive presence-absence dataset (Dataset 1). The map was developed using ESRI ArcGIS 10 software (www.esri.com/arcgis). The topographic base of the map was compiled with Natural Earth Free Vector and Raster Map Data (www.naturalearthdata.com), and the HydroSHEDS database (www.hydrosheds.org)^119,120^. (Map: Mikhail Y. Gofarov).

Two completely different unionid faunas are recorded in Russia (Figs. 6 and 7, Dataset 1, and Supplementary Fig. 3). The first faunal group includes six species from three European genera (*Anodonta*, *Pseudanodonta*, and *Unio*) occurring throughout European Russia east to Siberia up to the Lena River basin. The second group covers the Siberian rivers east of the Lena Basin, coastal basins of the Far East, and the huge Amur Basin. This faunal group contains 10 species from eight East Asian genera (*Amuranodonta*, *Beringiana*, *Buldowskia*, *Cristaria*, *Lanceolaria*, *Middendorffinaia*, *Nodularia*, and *Sinanodonta*).

### Conservation assessment

Most species in the Russian fauna represent common taxa with broad distributions, and they are considered as Least Concern (LC): *Amuranodonta kijaensis*, *Anodonta anatina*, *A. cygnea*, *Beringiana beringiana*, *Buldowskia shadini*, *Cristaria plicata*, *Lanceolaria grayii*, *Middendorffinaia mongolica*, *Nodularia douglasiae*, *Pseudanodonta complanata*, *Sinanodonta schrenkii*, *Unio pictorum*, *U. tumidus*, and *U. crassus* (Dataset 2). However, *Buldowskia suifunica* and *Sinanodonta lauta* have restricted ranges and are assessed as Vulnerable (VU) and Endangered (EN), respectively (Dataset 2).

## Taxonomic Account

Family Unionidae Rafinesque, 1820

Subfamily Unioninae Rafinesque, 1820

**Tribe Anodontini Rafinesque, 1820**

**Genus*Anodonta*Lamarck, 1799**

=*Colletopterum* Bourguignat, 1880 (type species: *Anodonta letourneuxi* Bourguignat, 1870 = *Anodonta anatina*; subsequent designation by Simpson, 1900)^25^.

=*Piscinaliana* Bourguignat, 1881 (type species: *Anodonta piscinalis* Nilsson, 1823 = *Anodonta anatina*; subsequent designation by Locard (1890); unavailable name, primary junior homonym of *Piscinaliana* Paladilhe, 1866, Gastropoda, Valvatidae)^25^.

Type species: *Mytilus cygneus* Linnaeus, 1758 (monotypy).

Diagnosis. *Anodonta* is very similar to *Pseudanodonta* but can be distinguished by a broadly rounded anterior margin, a shorter hinge length from the umbo to the ligament’s posterior limit^45^, and shorter and less closely spaced papillae of the inhalant siphon^46^.

Distribution. Two species of this genus are recorded from Russia (Fig. 2A–D and Table 1). *Anodonta anatina* inhabits European Russia, Urals, and Siberia eastwards to the Lena River basin (Fig. 6D), while the range of *A. cygnea* is restricted to water bodies of the Baltic, Black, Azov, and Caspian Sea drainage basins (Fig. 6E).

Comments. Phylogenetically, this genus-level clade appears to be paraphyletic because it contains *Pseudanodonta complanata* lineage (Fig. 1). The taxonomy of these two genera has not been discussed due to insufficient molecular data on *Anodonta* species from North America.

**Genus*Pseudanodonta*Bourguignat, 1876**

Type species: *Anodonta complanata* Rossmässler, 1835 (subsequent designation by Westerlund, 1902)^25^.

Diagnosis. See above.

Distribution. *Pseudanodonta complanata* (Fig. 2E) inhabits rivers of the Baltic, Black, Azov, and Caspian Sea drainage basins (Table 1 and Fig. 6E).

Comments. This genus may represent a junior synonym of *Anodonta*.

**Tribe Cristariini Lopes-Lima*et al****.***, 2017**

**Genus*Amuranodonta*Moskvicheva, 1973**

=*Amurbuldowskia* Bogatov & Starobogatov, 1996 (type species: *Buldowskia* (*Amuranodonta*) *boloniensis* Zatravkin & Bogatov, 1987; original designation)^22,25^.

Type species: *Amuranodonta kijaensis* Moskvicheva, 1973 (original designation).

Diagnosis. This genus is most similar to *Buldowskia*, *Anemina* s. str., and *Beringiana* but can easily be distinguished from these taxa by an elongated shell with a characteristic elongated, triangular posterior margin (vs ovate or rounded shell with rather rounded posterior margin).

Distribution. This genus (Fig. 3G,H) seems to represent a lineage endemic to the Amur Basin (Fig. 6C and Table 1). It is also known to occur in the Chinese part of the Ussuri River^47^.

Comments. This monotypic genus represents a lineage that is phylogenetically distant from the *Buldowskia* and *Anemina* s. str. clades (mean *COI* p-distance = 15.0% and 14.6%, respectively) (Fig. 1). Our first reviser action on the precedence of simultaneous synonyms: *Amuranodonta kijaensis* over *A. parva* (see Supplementary Note for explanation).

**Genus*Beringiana*Starobogatov in Zatravkin, 1983**

=*Kunashiria* Starobogatov in Zatravkin, 1983 (type species: *Anodonta japonica* Clessin, 1874; original designation)^25,48^.

=*Arsenievinaia* Zatravkin & Bogatov, 1987 (type species: *Amuranodonta sihotealinica* Zatravkin & Starobogatov, 1984; original designation)^20,25^.

Type species: *Anodonta cellensis* var. *beringiana* Middendorff, 1851 (original designation).

Diagnosis. This genus can be distinguished from *Amuranodonta* by a less elongated, inequilateral shell with a rounded posterior margin and from *Buldowskia* by a comparatively weakly pronounced umbo. Both *Cristaria* and *Sinanodonta* have much higher, elliptical shells, although several morphological varieties of *B. beringiana* from small lakes have short, rounded shells with broad posterior margin (Fig. 4H).

Distribution. *Beringiana beringiana* occurs in Russia, being widespread in rivers and lakes east of the Lena Basin, coastal rivers of the Japan and Okhotsk Sea drainage basins northeast of Vladivostok (from the Kievka River), and on the North Pacific Islands (Kuriles and Sakhalin) (Fig. 6B and Table 1). This is a conchologically variable species, with the largest number of synonyms introduced for its morphotypes from different water bodies of the Russian Far East (Fig. 4F–H and Supplementary Note). Outside Russia, this species is known from Alaska, Western Pacific Region, and Canada in North America, and from Japan^44,49^.

Comments. Williams *et al*.^49^ assumed that *Beringiana* is a junior synonym of *Sinanodonta*, but this genus represents a distant genus-level phylogenetic lineage and the two are here treated as distinct taxa (Fig. 1). *Kunashiria* is a synonym of *Beringiana*, because its type species, *B. japonica*, belongs to the latter genus^44^.

**Genus*Buldowskia*Moskvicheva, 1973**

Type species: *Anodonta arcaeformis* var. *suifunica* Lindholm, 1925 (original designation).

Diagnosis. This genus can be distinguished from *Amuranodonta* by an ovate shell with a rounded posterior margin, and from *Beringiana* by a comparatively pronounced umbo, while in some lacustrine populations of *Buldowskia shadini* the umbo is not pronounced (Fig. 3E). *Buldowskia* and *Anemina* s. str. are almost indistinguishable morphologically.

Distribution. Two *Buldowskia* species are recorded in Russia (Fig. 3A–F and Table 1). This genus is distributed in the Amur and Razdolnaya River basins, and in the coastal rivers southwest of Vladivostok (Fig. 6A,C).

Comments. This genus is phylogenetically distant from the *Amuranodonta* and *Anemina* s. str. clades (mean *COI* p-distance = 15.0% and 16.7%, respectively) (Fig. 1). In addition to *Buldowskia suifunica* and *B. shadini*, it contains two lineages endemic to South Korea, i.e. *B. flavotincta* (Martens, 1905) and *B. iwakawai* (Suzuki, 1939), and a species new to science from Japan^44,50^. Our first reviser action on the precedence of simultaneous synonyms: *Buldowskia shadini* over *B. fuscoviridis* and *B. buldowskii* (see Supplementary Note for explanation).

**Genus*Cristaria*Schumacher, 1817**

Type species: *Cristaria tuberculata* Schumacher, 1817 (monotypy).

Diagnosis. *Cristaria* species can be distinguished from those of *Sinanodonta* by the presence of reduced lateral teeth, which *Sinanodonta* species lack, the absence of pseudocardinal teeth, and a comparatively thick shell. Additionally, *Cristaria* species have a well-developed dorso-posterior keel and a more angulate, higher shell with clear angle between dorsal and posterior margin, although *Sinanodonta schrenkii* often has an angulate shell with clear angle between dorsal and posterior margin.

Distribution. *Cristaria plicata* was recorded throughout the Amur Basin, and in the Tym’ River, central Sakhalin^51^ (Figs. 4E, 6C and Table 1). There are several records of subfossil shells of this species from the Pleistocene deposits in the Tym’ Valley^6,52^.

**Genus*Sinanodonta*Modell, 1945**

=*Cristariopsis* Moskvicheva, 1973 (type species: *Sinanodonta* (*Cristariopsis*) *crassitesta* Moskvicheva, 1973; original designation)^19,25^.

=*Ellipsanodon* Bogatov & Starobogatov, 1996 (type species: *Sinanodonta* (*Ellipsanodon*) *ovata* Bogatov & Starobogatov, 1996; original designation)^21,25^.

Type species: *Symphynota magnifica* Lea, 1834 (by typification of a replaced name)^25^.

Diagnosis. This genus can be distinguished from *Cristaria* by the lack of lateral teeth and a comparatively thin, rather fragile shell. Usually, *Sinanodonta* taxa have more ovate shells with a rather rounded angle between dorsal and posterior margin and a weakly developed or lacking dorso-posterior keel. However, *Sinanodonta schrenkii* often has an angulate shell with clear angle between dorsal and posterior margin.

Distribution. Two native *Sinanodonta* species are recorded in Russia (Fig. 2F–H and Table 1). This genus is distributed in the Amur and Razdolnaya basins, and in the coastal rivers southwest of Vladivostok (Fig. 6A,B). The non-native species *Sinanodonta woodiana* was recorded from the Yenisei River, in which it was found in a thermally polluted river channel in sympatry with an introduced population of *S. lauta*^10^ (Fig. 6A).

**Tribe Lanceolariini Froufe*et al****.***, 2017**

**Genus*Lanceolaria*Conrad, 1853**

=*Cylindrica* Simpson, 1900 (type species: *Nodularia cylindrica* Simpson, 1900; original designation; unavailable name, primary homonym of *Cylindrica* Clessin, 1882, Gastropoda, Hydrobiidae)^25^.

=*Pericylindrica* Tomlin, 1930 (replacement name for *Cylindrica* Simpson, 1900)^25^.

=*Prolancealaria* Moskvicheva, 1973 (type species: *Unio grayii* Griffith & Pidgeon, 1833; original designation)^7,25^.

Type species: *Unio grayanus* Lea, 1834 (monotypy).

Diagnosis. This genus can easily be distinguished from the other Russian Unionidae by its unique lanceolate shell shape.

Distribution. *Lanceolaria grayii* inhabits Lake Khanka, Ussuri Basin, and Lower Amur (Figs. 4D, 6A and Table 1), representing the most northern enclave for this remarkable lineage of the Yangtze Basin fauna^44,53^.

**Tribe Unionini Rafinesque, 1820**

**Genus*Middendorffinaia*Moskvicheva & Starobogatov, 1973**

=*Suifununio* Moskvicheva & Starobogatov, 1973 (type species: *Middendorffinaia* (*Suifununio*) *suifunensis* Moskvicheva & Starobogatov, 1973; original designation)^18,25^.

=*Pseudopotomida* Moskvicheva & Starobogatov, 1973 (type species: *Middendorffinaia* (*Pseudopotomida*) *shadini* Moskvicheva & Starobogatov, 1973; original designation)^18,25^.

Type species: *Unio mongolicus* Middendorff, 1851 (original designation).

Diagnosis. This monotypic genus is externally similar to *Nodularia*, from which it can be distinguished by a higher, shorter shell, a strongly convex or even angulate hinge plate, more convex dorsal margin, and fine umbonal sculpture with small regular tubercles and narrow ridges in young shells. However, umbonal sculpture is often weakly developed or absent.

Distribution. *Middendorffinaia mongolica* is distributed in the Amur and Razdolnaya river basins, and in coastal rivers west of Nakhodka^54^ and southwest of Vladivostok (Figs. 5A–D, 6G and Table 1). The record from a coastal river of the Okhotsk Sea drainage basin^40,55^ is *Nodularia douglasiae* (Fig. 5F).

Comments. Middendorff (p. 277)^12^ described his *Unio mongolicus* based on a single specimen, which must be considered the holotype (by monotypy). The type locality was stated as follows: “Aus einem Gebirgsbache ohnfern Gorbitza in Daurien” [Russia, Transbaikalia, a mountain spring near Gorbitsa village (53.1027°N, 119.2169°E)]. The holotype was lost a long time ago, at least before 1973^18,20,24,25^. The two figures of Middendorff^12^ show outside of the left valve and dorsal side of the shell. A relatively high shell with convex dorsal margin and umbo situated near the anterior margin indicates that *Unio mongolicus* sensu Middendorff is a distinct species, not a synonym of *Nodularia douglasiae*.

Zhadin^5^ assumed that *Unio mongolicus* sensu Middendorff is a rare member of the Margaritiferidae, and placed it in the genus *Margaritana* Schumacher, 1817. However, in his later work this species was called *Unio douglasiae* var. *mongolicus* with a question mark^6^. Moskvicheva and Starobogatov^18^ identified several specimens from the Ussuri Basin as prospective representatives of *Unio mongolicus*, but their specimens belong to *Nodularia douglasiae* (Fig. 5G). A new genus, *Middendorffinaia*, was established, with *Unio mongolicus* Middendorff as its type species^18^. This genus included three subgenera: *Middendorffinaia* s. str., *Pseudopotomida*, and *Suifununio*. Taxa placed within *Middendorffinaia* s. str., with exception of *Unio mongolicus* sensu Middendorff, belong to *Nodularia douglasiae*. In contrast, the *Pseudopotomida* and *Suifununio* species represent conchological varieties of *Unio mongolicus* sensu Middendorff (Supplementary Note). Two more such varieties are described as separate nominal species^7,24^.

Graf^26^ placed *Middendorffinaia* s. str. taxa as synonyms of *Unio crassus mongolicus* Middendorff, and *Pseudopotomida* and *Suifununio* taxa as synonyms of *Inversidens pantoensis* (Neumayr, 1899). This point of view highlighted differences between *Unio mongolicus* sensu Middendorff (with *Pseudopotomida* and *Suifununio* taxa) and *U. mongolicus* sensu Moskvicheva and Starobogatov (with their additional *Middendorffinaia* s. str. taxa belonging to *Nodularia douglasiae*). However, *Unio mongolicus* Middendorff with its varieties (*Pseudopotomida* and *Suifununio* spp.) is phylogenetically and morphologically distant from both the European *Unio* and East Asian *Inversidens*^56,57^.

Klishko *et al*.^40^ followed the concept of *Unio mongolicus* sensu Moskvicheva and Starobogatov^18^ and pictured a *Nodularia douglasiae* shell collected near Gorbitsa village as the prospective topotype of this taxon. It was stated that the holotype dimensions in Middendorff’s protologue does not correspond to the proportions of the shell pictured in his book (Pl. 27, Figs. 7–8 ^12^), and that this original holotype picture was “digitally corrected according to the measurements of Middendorff”^40^. However, this statement is not entirely true, because the shell height vs shell length ratio is 0.47 and 0.46 by the original image and by Middendorff’s measurements^12^, respectively. This difference is too small and seems to reflect rather slightly inaccurate original measurements than the incorrect holotype picture of *Unio mongolicus* sensu Middendorff.

To retain the original concept of *Unio mongolicus* sensu Middendorff as a taxon distinct from *Nodularia douglasiae*, and to secure the stability of nomenclature, we designate the sequenced specimen RMBH biv229_5 labelled “Russia, Primorye Region, downstream of Gladkaya River (42.7065°N, 130.9084°E), 26.x.2016, Bolotov and Vikhrev leg.” as the neotype of this species (Fig. 5A). The reference sequences accession numbers for the neotype are as follows: MH974549 for *COI*, MK574414 for *16S rRNA*, and MK574555 for *28S rRNA*. The shell measurements are as follows: shell length 71.7 mm, height 37.6 mm, width 27.3 mm. The neotype is designated in accordance with the conditions specified in Art. 75 of ICZN, because the name-bearing type specimen was lost, and the authors consider that a name-bearing type is necessary to define the nominal taxon objectively and to avoid further speculations on this issue. We designated a specimen from the Gladkaya River as the neotype, because in this sequenced sample (three genes), we found a specimen that is nearly identical externally to the lost Middendorff’s holotype. The *COI* sequence of the neotype is very similar to that obtained from a specimen collected from the Shilka River, relatively close to the Middendorff’s type locality (uncorrected *p*-distance = 0.70%). A sequenced sample from the original type locality is not available.

**Genus*Nodularia*Conrad, 1853**

=*Amurunio* Zatravkin & Bogatov, 1987 (type species: *Nodularia lebedevi* Zatravkin & Starobogatov, 1984; original designation)^20,25^.

=*Magadaninaia* Martynov & Chernyshev, 1992 (type species: *Nodularia* (*Magadaninaia*) *extremalis* Martynov & Chernyshev, 1992; original designation)^25,58^.

Type species: *Unio douglasiae* Griffith & Pidgeon, 1833 (monotypy).

Diagnosis. This genus is externally similar to *Middendorffinaia*, but can be distinguished from it by a narrower, elongated shell, an almost straight hinge plate, comparatively straight or slightly convex dorsal margin, and umbonal sculpture with W-shaped, broad ridges in young shells. However, umbonal sculpture is often weakly developed or absent.

Distribution. *Nodularia douglasiae* is widespread in the Amur and Razdolnaya basins, in several coastal rivers of the Okhotsk Sea drainage basin up to the Ola River just north of the city of Magadan, and in northwestern Sakhalin (Figs. 5E–H, 6H and Table 1). This species has a plethora of taxonomic names introduced for its conchological varieties from different parts of the Russian Far East (Supplementary Note).

**Genus*Unio*Retzius, 1788**

=*Tumidiana* Servain, 1882 (type species: *Unio tumidus* Retzius, 1788; subsequent designation by Kantor & Sysoev, 2005)^25^.

=*Crassiana* Servain, 1882 (type species: *Unio crassus* Retzius, 1788; subsequent designation by Graf, 2010)^25^.

Type species: *Mya pictorum* Linnaeus, 1758 (subsequent designation by Turton, 1831^25^).

Diagnosis. There are no conchologically similar genera in European Russia and the Urals but introduced populations in the Upper Amur Basin^9^ can be mistaken with *Nodularia* and *Middendorffinaia*. *Nodularia* has a more elongated, comparatively cylindrical shell. *Middendorffinaia* differs from *Unio* by a strongly convex hinge plate and more developed pseudocardinal teeth.

Distribution. Three *Unio* species were recorded from Russia (Figs. 4A–C, 6E,F, and Table 1). This genus is widely distributed in European Russia and Western Urals, with an isolated native population of *Unio tumidus* in the Irtysh Basin in Western Siberia^59^ and Kazakhstan (Table 1). There were a few occasional records of *Unio* from the Ob’-Irtysh Basin since the middle of the 19th century^5,60–62^. Non-native populations of *Unio pictorum* and *U. tumidus* are known to occur in the Upper Amur Basin (Lake Kenon) in Transbaikalia^9^ (Fig. 6F).

## Discussion

### Taxonomic richness of the Unionidae fauna in Russia

Our results support the conclusion that the Russian Unionidae fauna is rather species-poor^5,6,26^, with only 16 native species belonging to 11 genera of a single subfamily, the Unioninae. Most freshwater mussels in Russia belong to the tribe Cristariini^41^, which includes five genera (*Amuranodonta*, *Beringiana*, *Buldowskia*, *Cristaria*, and *Sinanodonta*) and seven native species inhabiting the Far East. The tribe Unionini^63^ contains five species in three genera (*Middendorffinaia*, *Nodularia*, and *Unio*), the Anodontini^41^ includes three species in two genera (*Anodonta* and *Pseudanodonta*), and the Lanceolariini^41^ holds one *Lanceolaria* species.

The species richness of unionid mussels in Russia represented by previous morphology-based taxonomic schemes^23,25,64^ has been dramatically overestimated. Nearly 100 taxa of freshwater mussels, including 70 species-group names and 14 genus-group names were described in Russia as new to science since the introduction of the so-called comparatory method in the early 1970s^25,26,65^ (Table 1 and Supplementary Note). This method is based on an assumption that the contour of the shell valve frontal section is taxon-specific and, as such, can be used as a single diagnostic feature to distinguish species, genera, and even family-group bivalve taxa^23,25^. Furthermore, a variety of old synonyms for several species and genera were resurrected as valid names using minute differences in the curvature of the shell frontal section^25,64,66^. However, the shell convexity is strongly influenced by habitat parameters and climatic factors and cannot be used as a diagnostic character^3,5,9,26–28,31,35,38^. A growing body of research critically reassessing the comparatory method in and outside of Russia has discredited its usage for taxonomy, and the last “comparatory” species, *Middendorffinaia alimovi* (=*M. mongolica*), was described in 2012^24^.

According to our results, only six unionid taxa described in Russia during the “comparatory” period are valid, i.e. four genera (*Amuranodonta*, *Beringiana*, *Buldowskia*, and *Middendorffinaia*) and two species (*Amuranodonta kijaensis* and *Buldowskia shadini*). A plethora of other names reflecting ecophenotypic shell variability within unionid species was synonymized by recent reviewers^9,26,34,35,38–40^ and in this study. In summary, each biological species in Russia has 4.4 ± 1.1 (mean ± s.e.m.; *N* = 16) “comparatory” synonyms introduced by Starobogatov’s school (Table 1). This mean rate of synonymy for the national fauna of Unionidae is close to that for the freshwater pond snails, the Lymnaeidae, in which four morphological taxa appear to represent a single valid biological species^67^. However, this value is higher for the fauna of the Russian Far East, with 6.2 ± 1.5 “comparatory” names per biological species (*N* = 10). The highest synonymy load is characteristic for conchologically variable species with broad ranges, i.e. *Beringiana beringiana* (16 species-group and 2 genus-group names), *Nodularia douglasiae* (9 species-group and 2 genus-group names), and *Middendorffinaia mongolica* (8 species-group and 3 genus-group names). In contrast, *Anodonta cygnea*, *Cristaria plicata*, *Unio tumidus*, and *U. crassus* have no new “comparatory” names (Table 1), while each of these biological species was also divided into several morpho-taxa named using available historical synonyms^23,25,64^.

### Biogeography of the Unionidae in Russia

Based on the results of our PCA analysis of Unionidae species ranges (Supplementary Fig. 3 and Dataset 1), the country area can be delineated into the northern parts of two subregions of the Palearctic Region, i.e. Western Palearctic and Eastern Palearctic (East Asian) subregions (Fig. 7), which are briefly described below.

(1) Western Palearctic Subregion covers most of the country from its western boundary to the Lena River Basin in Eastern Siberia. Outside Russia, this subregion encompasses countries of Europe and Central Asia westward to the Middle East and North Africa. In Russia, this area is inhabited by six native species: *Anodonta anatina*, *A. cygnea*, *Pseudanodonta complanata*, *Unio tumidus*, *U. pictorum*, and *U. crassus*.

(1.1) North European Province covers water bodies of the Arctic Ocean drainage (e.g. large basins of the Pechora, Northern Dvina, and Onega rivers), with three species: *Anodonta anatina*, *Unio tumidus* and *U. pictorum*. Several small and medium-sized rivers in the northern part of this province are inhabited only by *Anodonta anatina* (e.g. Kem, Keret, Mudyuga, and Indiga rivers), but this pattern is likely caused by environmental conditions rather than historical biogeographic events. A non-native population of *Anodonta anatina* was established in a warm water channel of the Kola Nuclear Power Plant^68^.

(1.2) East European Province covers water bodies of the Azov, Black, Caspian, and Baltic Sea drainage basins (e.g. huge basins of the Volga and Don rivers) and is inhabited by all six species known from the subregion.

(1.3) Siberian Province covers Siberia eastwards to the Lena River (e.g. Ob’, Irtysh, Taz, Yenisei, and Lena rivers). *Anodonta anatina* primarily inhabits water bodies in this area, while local populations of *Unio tumidus* were discovered in the Irtysh Basin in Western Siberia^59^ and Kazakhstan. The latter population can be considered native, because a few historical records of *Unio* are known from the southern part of the Ob’-Irtysh Basin^5,60–62^. *Sinanodonta lauta* and *S. woodiana* were introduced to the Yenisei River^10^. *Unio tumidus* and *U. pictorum* were introduced to the Upper Amur Basin in Transbaikalia^9^. Based on the COI gene sequences, the non-native *Unio* populations in the Upper Amur Basin may have been originated from rivers of the Black Sea drainage, e.g. Dnieper or Danube (Supplementary Table 1 and Supplementary Note).

(2) Eastern Palearctic (East Asian) Subregion covers the Amur Basin, rivers east of the Lena Basin, coastal rivers of the Okhotsk and Japan Sea drainage basins, and the North Pacific Islands (Sakhalin and Kuriles). Beyond Russia, this subregion extends throughout Mongolia, Korea, continental China, Japan, Taiwan, Hainan Island south to central Vietnam^44,69,70^. In Russia, this subregion is inhabited by 10 native species: *Amuranodonta kijaensis*, *Beringiana beringiana*, *Buldowskia suifunica*, *B. shadini*, *Cristaria plicata*, *Lanceolaria grayii*, *Middendorffinaia mongolica*, *Nodularia douglasiae*, *Sinanodonta lauta*, and *S. schrenkii*.

(2.1) North Asian Province covers rivers of the Kolyma Highlands (Kolyma and Indigirka rivers), Koryak Region, Chukotka, Kamchatka, North Pacific Islands (Sakhalin and Kurile Archipelago), coastal rivers of the Okhotsk and Japan Sea drainage basins northeast of Nakhodka (from the Kievka River). This severe area with mountainous landscapes and cold climate is primarily inhabited by *Beringiana beringiana*, but there are a few records of *Nodularia douglasiae* in several coastal rivers of the Okhotsk Sea^39,55^, and *N. douglasiae* and *Cristaria plicata* from Sakhalin^51,71^. Based on the phylogeographic patterns of freshwater fishes^72,73^, the Yana River basin could also be placed within this province, although its Unionidae fauna is unknown^74^ and needs a special research effort.

(2.2) Amur Province covering the Amur Basin and small rivers surrounding its mouth is the most species-rich freshwater system in Russia, with seven native species. Most species have vast distribution ranges throughout East Asia to South Korea (*Buldowskia shadini* and *Sinanodonta schrenkii*), Yangtze Basin in eastern China (*Lanceolaria grayii*) or even northern Vietnam (*Cristaria plicata* and *Nodularia douglasiae*)^44^, while *Amuranodonta kijaensis* and *Middendorffinaia mongolica* seem to be endemic lineages to this province^44^, partly spreading to the adjoining transition zone (see below).

(2.3) Amur–Korean–Japanese Biogeographic Transition Zone (BTZ) with five native species covers the Razdolnaya River basin, and smaller coastal rivers of the Japan Sea drainage basin west of Nakhodka (Partizanskaya and Artemovka rivers^54^) and southwest of Vladivostok to the boundary with North Korea and China. Its fauna represents a mix of Amur, Korean, Japanese, and Chinese elements, i.e. *Middendorffinaia mongolica* (Amur), *Nodularia douglasiae* (eastern China, Korea, Japan, and northern Vietnam), *Sinanodonta schrenkii* (Amur and Korea), and *S. lauta* (Korea and Japan)^44^. *Buldowskia suifunica* seems to have a narrow range restricted to the BTZ, but it can also inhabit North Korea and northeastern China (e.g. the nearest Tumen Basin). The Amur freshwater pearl mussel *Margaritifera dahurica* (Margaritiferidae) and Japanese mussel leech *Batracobdella kasmiana* (Glossiphoniidae) are known from this area^29,75^ representing two more examples of such a faunal intermixing in other groups of aquatic invertebrates supporting the delineation of the BTZ^76^.

In summary, no unionid species endemic to Russia has been identified, except for *Buldowskia suifunica*. However, this species inhabits the Razdolnaya Basin, a section of which is located in China, and may occur there. *Amuranodonta kijaensis* can be considered a putative single-basin endemic to the Amur Basin in Russia and China, while *Middendorffinaia mongolica* appears to be a lineage endemic to the Amur and Razdolnaya basins and few more coastal rivers. Besides these three species, generalist taxa with a broad distribution crossing a variety of drainage divides predominate in the country’s fauna. Furthermore, the fauna of the Eastern Palearctic Subregion in Russia is strongly influenced by Japanese (*Beringiana beringiana* and *Sinanodonta lauta*)^43,44^, Korean (*Buldowskia shadini* and *Sinanodonta schrenkii*)^44,50^, and Yangtze (*Cristaria plicata*, *Lanceolaria grayii*, and *Nodularia douglasiae*)^43,44,53^ lineages. This biogeographic pattern strongly differs from that in the Yangtze, Mekong and Irrawaddy basins, in which the proportion of single-basin and even intra-basin endemic lineages is much higher, with only a few widespread species^53,69,77–81^. In its turn, the Unionidae fauna of the Western Palearctic Subregion in Russia, including its Siberian Province, seems to be completely allochthonous and was likely originated from glacial refugia in southern basins of the Baltic Sea, and drainages of the Caspian, Black and Azov seas^82,83^. Our results are consistent with biogeographic patterns discovered in several freshwater fishes, i.e. rapid post-glacial dispersal events from refugia in the Ponto-Caspian Region to the Volga Basin^84,85^ and Siberia up to the Lena Basin^85^.

*Anodonta anatina* and *Beringiana beringiana* seem to be the most cold-tolerant species among the Unionidae, the ranges of which cross the Arctic Circle (66.56°N) and reach the Arctic Ocean coast via several freshwater basins (Fig. 6B,D). In contrast, *Sinanodonta lauta* and *Buldowskia suifunica* appear to be rather thermophilic species restricted to the extreme south of the Russian Far East up to 44°N, while *Lanceolaria grayii* inhabits the Ussuri Basin and an adjoining section of the Amur River up to 50°N, but does not spread throughout the Amur Basin (Fig. 6A).

Our updated biogeographic division of the Northern Palearctic based on unionid mussel fauna is largely congruent with that of Graf and Cummings^86^. However, these authors separated four subregions: Europe, Central Asia, Amur-Beringia with northern China and Korea, and Japan-Sakhalin including Kurile Archipelago^86^, corresponding to our Western Palearctic (Europe + Central Asia) and Eastern Palearctic (Amur-Beringia + Japan-Sakhalin) subregions. Our novel results support the hypothesis of Moskvicheva^7^ on significant faunal differences between the Amur Basin and coastal rivers in the southern edge of the Primorye Region (our Amur–Korean–Japanese BTZ). The close relationship between the Unionidae faunas of the latter area and Korea predicted by this author^7^ was also supported by our research. Moskvicheva^7^ delineated several biogeographic provinces within the Amur Basin (e.g. Ussuri, Khabarovsk, Argun-Zeya, and Sunggari [Songhua] provinces) based on their intra-basin endemic species, but all these taxa were found to be morphological varieties of broadly distributed lineages. Based on newly obtained results, Moskvicheva’s provinces within the Amur Basin should be joined into one province. There are some faunal differences between various parts of the Amur River system (e.g. freshwater mussels appear to be lacking in the headwaters and middle reaches of the Amgun River^7^), but this seems to be caused by recent environmental conditions rather than historical biogeographic events. Our biogeographic division also agrees with that of Zhadin^5^. In contrast, the direct comparison of our scheme with that of Starobogatov^8^ is impossible, because this author combined all groups of Mollusca in his global biogeographic zoning.

### Conservation priorities

Based on the IUCN criteria, only *Buldowskia suifunica* and *Sinanodonta lauta* are assessed here as Vulnerable (VU) and Endangered (EN), respectively, while the other species having much broader ranges are considered Least Concern (LC). However, we recommend including five taxa to a new edition of the Red Data Book of Russia as rare species (Status 3) inhabiting a limited area (*Buldowskia suifunica* and *Sinanodonta lauta*) or sporadically distributed over an extensive area (*Amuranodonta kijaensis*, *Lanceolaria grayii*, and *Middendorffinaia mongolica*).

### Directions for future studies

This study clarifies the taxonomy of the Russian Unionidae and opens ways for further biological and ecological investigations of valid species that were hampered for more than 40 years by the comparatory systematics with multiple conchological morphs erroneously erected to the species rank. While host fishes and life cycles of the six European species are rather well described outside Russia^42^, those of taxa from East Asia, especially endemic species to the Russian Far East and Korea such as *Amuranodonta kijaensis*, *Buldowskia shadini*, *B. suifunica*, *Middendorffinaia mongolica*, and *Sinanodonta schrenkii* need special research efforts. The population structure and dynamics, growth patterns, and maximum age of these East Asian taxa are virtually unknown.

Several species with broad ranges such as *Anodonta anatina*, *A. cygnea*, *Beringiana beringiana*, *Cristaria plicata*, *Middendorffinaia mongolica*, *Nodularia douglasiae*, *Pseudanodonta complanata*, *Sinanodonta schrenkii*, *Unio pictorum*, *U. tumidus*, and *U. crassus*, appear to be appropriate models for phylogeographic studies with a supplement of molecular data from adjacent countries such as Kazakhstan, China, Mongolia, Korea, Japan, and others. A few available works on this issue have revealed putative colonization and refugial patterns for several European and Chinese species^87–91^ that are of great importance to reconstruct the evolutionary history of freshwater fauna in the Palearctic Region. Widespread species can also be used as models for broad-scale studies of intraspecific shell variability^92,93^. It is likely that environment-induced shifts in the shell shape can be traced in species with extremely high levels of conchological variability such as *Anodonta anatina*, *Beringiana beringiana*, *Buldowskia suifunica*, *B. shadini*, *Middendorffinaia mongolica*, and *Nodularia douglasiae*. Furthermore, the shell convexity that was used to delineate comparatory taxa actually reflects shifts in summer temperatures and can be applied as a sensitive and low-cost indicator of climate changes^3,94^.

Reliable fossil records are essential to reconstruct robust fossil-calibrated phylogenies using multi-locus and mitogenomic approaches^77,78,81,95^. Paleontologists described numerous fossil species of freshwater mussels from Russia and adjacent countries^96–100^. However, a critical taxonomic revision of all these taxa is urgently needed to clarify their status, prospective phylogenetic placement, and validity. Multiple fossil species recovered from the Pleistocene deposits^98^ should be compared with recent representatives of the corresponding genera, as many of these nominal taxa may be synonyms of terminal species or their stem lineages^33,101,102^.

Finally, the Unionidae faunas of several large freshwater basins in the Russian Far East and Siberia, e.g. the Anabara River (Yakutia), Yana River (Kolyma Highlands), Penzhina River (Koryak Region), Uda River (Khabarovsk Region), and Amguema River (Chukotka Peninsula), remain unknown. However, these water bodies are surrounded by relatively well-studied freshwater systems and will hardly deliver any species new to science. In contrast, the neighboring Chinese provinces (Heilongjiang, Jilin, Liaoning, and Inner Mongolia) and North Korea seem to be crucial areas to further understanding the taxonomy and distributional patterns of freshwater mussels in Northeast Asia. The freshwater basins in these areas may harbor additional populations of near threatened species (e.g. *Buldowskia suifunica* and *Middendorffinaia mongolica*) and probably a few still undescribed endemic lineages. Now we know almost nothing about the freshwater mussel fauna in these regions^43^, and such an extensive knowledge gap in freshwater malacology must be a focus of international collaborative research efforts.

## Methods

### Data sampling

In this study, we studied freshwater mussel specimens collected throughout Russia, i.e. from European Russia, Western Siberia, Eastern Siberia, Russian Far East, Sakhalin Island, and Kurile Archipelago. Available lots were studied in the following collections:

- RMBH – Russian Museum of Biodiversity Hotspots, Federal Center for Integrated Arctic Research of the Russian Academy of Sciences, Arkhangelsk, Russia;
- ZISP – Zoological Institute of the Russian Academy of Sciences, Saint Petersburg, Russia;
- INREC – Institute of Natural Resources, Ecology and Cryology, Siberian Branch of the Russian Academy of Sciences, Chita, Russia;
- NCSM – North Carolina Museum of Natural Sciences, Raleigh, North Carolina, United States of America.

### Molecular analyses

*COI*, *16S rRNA* (female mitochondrial DNA) and *28S rRNA* partial gene sequences were generated from 232 freshwater mussel specimens using a standard approach following our previous works^69,77,78^. Additional sequences were obtained from NCBI GenBank. *Margaritifera* species and representatives of Gonideinae and Ambleminae subfamilies were used as outgroup. The data set is presented in Supplementary Table 1.

### Morphological investigation

For comparative studies, we analyzed the shell shape, structure of pseudo-cardinal and lateral teeth, muscle attachment scars, and the sculpture and position of umbo^78,80^. The type series of nominal taxa under discussion and other lots of freshwater mussels from museum collections, original descriptions and figures from appropriate scientific literature, and available images from the MUSSELp database^103^ were used for morphological investigations.

### Phylogenetic analyses

The sequence alignments of the *COI*, *16S rRNA* and *28S rRNA* gene fragments were processed and joined as described in our previous works^69,77,78^. The combined data set (total length of 1878 bp) was collapsed from 363 available haplotypes into a set of 199 unique haplotypes using an online FASTA sequence toolbox (FaBox v1.41)^104^. Five partitions (3 codons of *COI* + *28S rRNA* + *16S rRNA*) were used for phylogenetic analysis. Maximum likelihood phylogenetic searches were performed through web-server for IQ-TREE (W-IQ-TREE) with an automatic identification of the best-fit substitution model for each partition^105,106^ (Supplementary Table 2). An ultrafast bootstrap (UFBoot) algorithm with 7,000 replicates was implemented for estimation of the internal branches probability^107^. Bayesian analyses were performed in MrBayes v. 3.2.6^108^. The HKY evolutionary model was applied for each partition. We used four runs, each with three heated (temperature = 0.1) and one cold Markov chain, using 50,000,000 generations with sampling every 1000th generation. All calculations were carried out at the San Diego Supercomputer Center through the CIPRES Science Gateway^109^. The first 25% of trees were discarded as burn-in. A convergence of the MCMC chains to a stationary distribution was checked through Tracer v. 1.7.1^110^.

### Biogeographic analysis and range mapping

We compiled a comprehensive presence-absence dataset on freshwater mussels (Unionidae) from freshwater basins of Russia (Dataset 1). To delineate the primary biogeographic units, we applied a PCA analysis algorithm implemented in PAST v. 3.04^111^ using this dataset. Component 1 and component 2 accounted for 43.5% and 20.2% of the total variance, respectively (Supplementary Fig. 3). The distribution maps for each species were created using ESRI ArcGIS 10 software (www.esri.com/arcgis).

### Conservation status assessment

Conservation status assessment for each species was based on the Guidelines for application of IUCN Red List criteria at regional and national levels v. 4^112^. The extent of occurrence (EOO) values were obtained from our distribution maps with ESRI ArcGIS 10 (rounded to the nearest thousand).

### Nomenclatural acts

The electronic edition of this article conforms to the requirements of the amended International Code of Zoological Nomenclature (ICZN), and hence the new combinations contained herein are available under that Code from the electronic edition of this article. This published work and the nomenclatural acts it contains have been registered in ZooBank (http://zoobank.org), the online registration system for the ICZN. The LSID for this publication is: urn:lsid:zoobank.org:pub:8BE71D2E-A2EE-4E30-AB17-70EA31F4D168. The electronic edition of this paper was published in a journal with an ISSN and has been archived and is available from PubMed Central.

### Data availability

The sequences used in this study are available from GenBank. Accession numbers for each specimen are presented in Supplementary Table 1. The shell vouchers, whole specimens, and tissue snips are available in the corresponding museum collections, i.e. RMBH, ZISP, INREC, and NCSM.

## Supplementary information

Supplementary information.

Dataset 1

Dataset 2
