# Supplementary material for: Integrative taxonomy, biogeography and conservation of freshwater mussels (Unionidae) in Russia
Source: Sci Rep. 2020 Feb 20;10:3072. doi: 10.1038/s41598-020-59867-7 (PMC7033218; doi:10.1038/s41598-020-59867-7)
Supplement: Supplementary file 1 — Supplementary information. [file 41598_2020_59867_MOESM1_ESM.pdf]

SUPPLEMENTARY INFORMATION

Integrative taxonomy, biogeography and conservation of freshwater mussels (Unionidae) in Russia

Ivan N. Bolotov*, Alexander V. Kondakov, Ekaterina S. Konopleva, Ilya V. Vikhrev, Olga V. Aksenova, Andrey S. Aksenov, Yulia V. Bespalaya, Alexey V. Borovskoy, Petr P. Danilov, Gennady A. Dvoryankin, Mikhail Y. Gofarov, Mikhail B. Kabakov, Olga K. Klishko, Yulia S. Kolosova, Artem A. Lyubas, Alexander P. Novoselov, Dmitry M. Palatov, Grigory N. Savvinov, Nikolay M. Solomonov, Vitaly M. Spitsyn, Svetlana E. Sokolova, Alena A. Tomilova, Elsa Froufe, Arthur E. Bogan, Manuel Lopes-Lima, Alexander A. Makhrov & Maxim V. Vinarski

*Corresponding author: inepras@yandex.ru

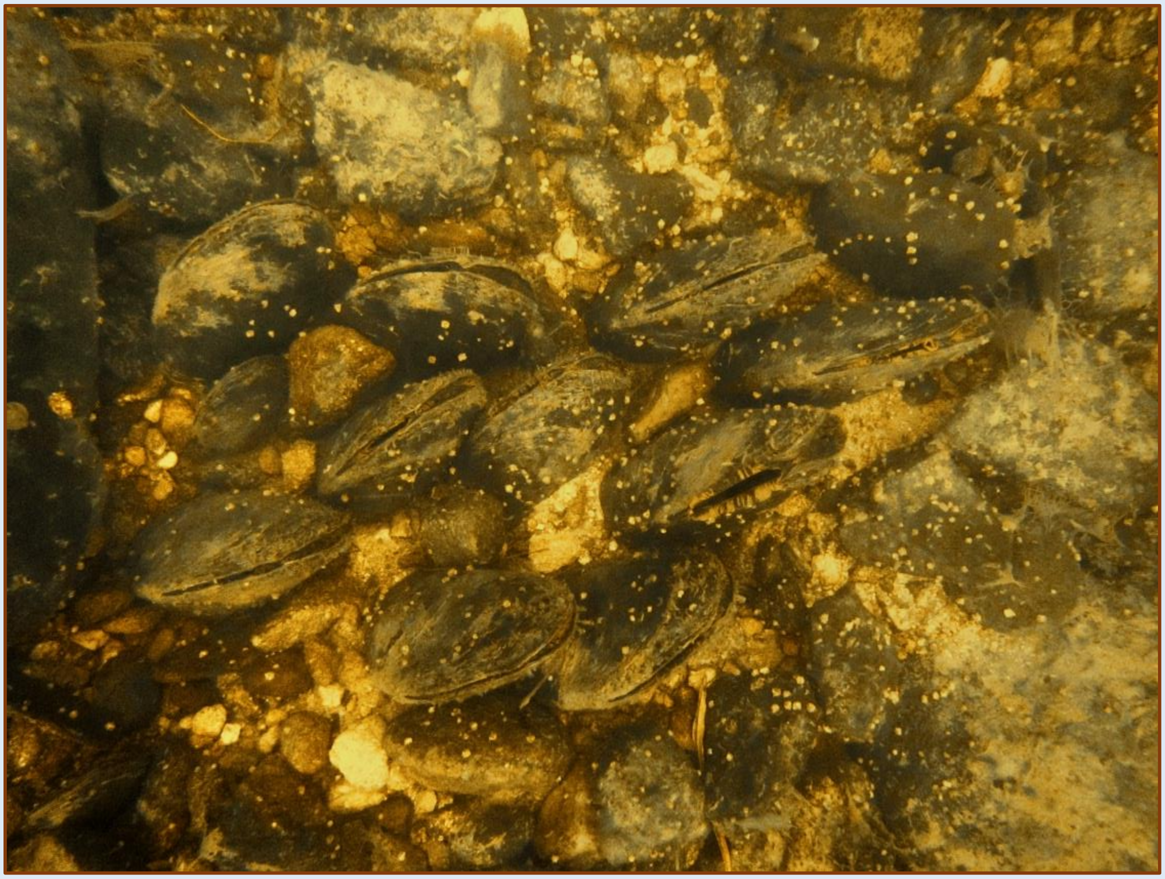

Freshwater mussel bed with *Unio tumidus*, Ust'ya River, Northern Dvina Basin, White Sea drainage (Photo: Olga V. Aksenova)

Contents

Supplementary Figures

Supplementary Figure 1. Habitats of freshwater mussels (Unionidae) in European Russia and Siberia..3

Supplementary Figure 2. Habitats of freshwater mussels (Unionidae) in the Russian Far East and Pacific Ocean islands…………………………..4

Supplementary Figure 3. Scatter plot of principal component analysis (PCA) based on the presence-absence dataset on freshwater mussels (Unionidae) throughout freshwater basins of Russia………………………5

Supplementary Tables

Supplementary Table 1. List of sequences used in this study, including the species, the location and NCBI’s GenBank accession numbers………....6

Supplementary Table 2. Models of sequence evolution for each partition based on Bayesian information criterion (BIC) of W-IQ-TREE……………23

Supplementary Note

Supplementary Note. Taxonomic review of the Unionidae species in Russia…………………………………………………………………………...24

Supplementary References

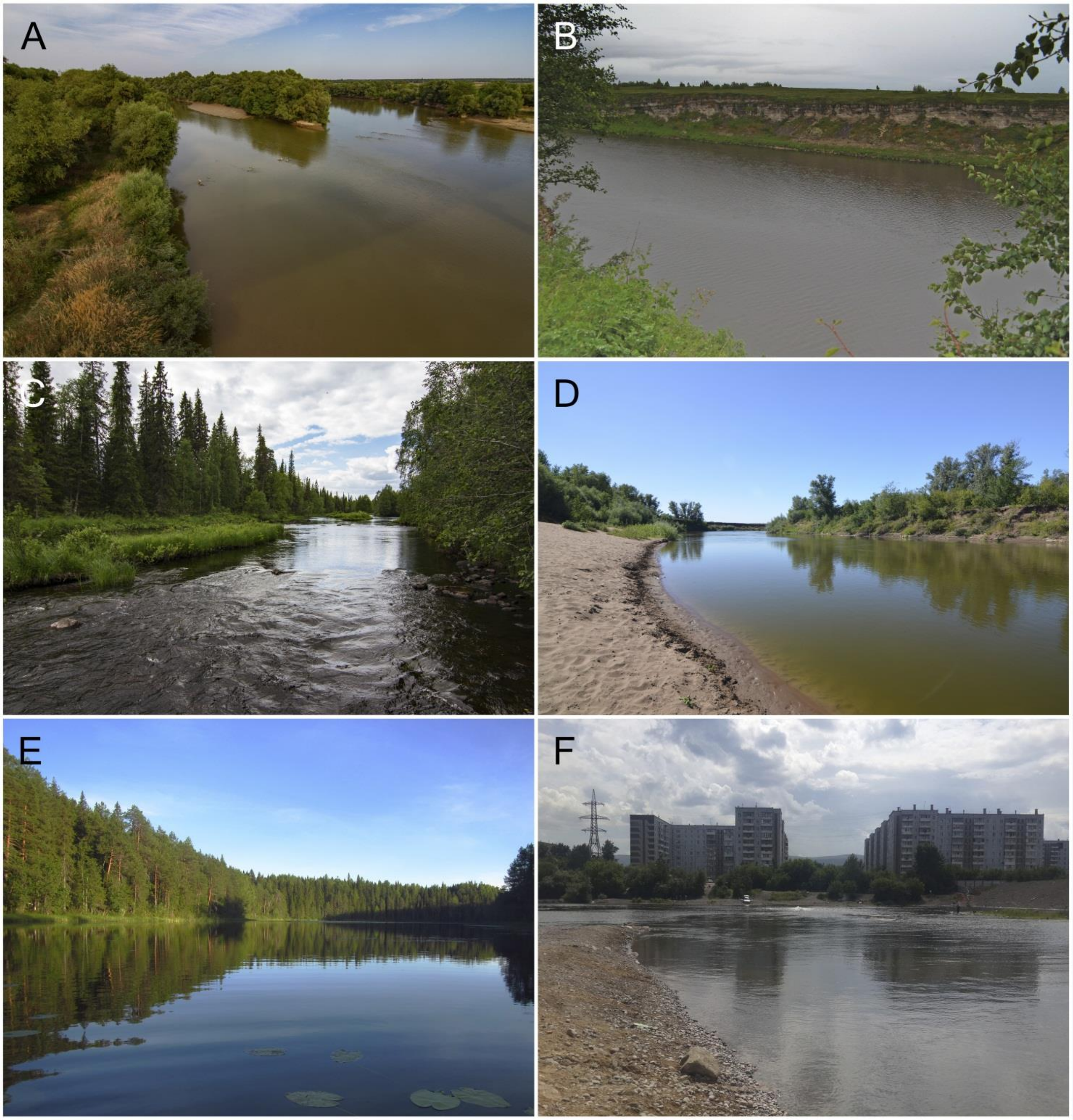

Supplementary Figure 1. Habitats of freshwater mussels (Unionidae) in European Russia and Siberia. (**A**) Kuban River, Azov Sea drainage (*Anodonta anatina*). (**B**) Indiga River, Barents Sea drainage (*A. anatina*). (**C**) Elet' River, Keret' River basin, White Sea drainage (*A. anatina*). (**D**) Samara River, Volga Basin, Caspian Sea drainage (*Unio crassus*, *U. pictorum*, and *U. tumidus*). (**E**) Lake Udelnoe, Northern Dvina Basin, White Sea drainage (*A. anatina*). (**F**) Krasnoyarsk, Yenisei River, Kara Sea drainage (non-native populations of *Sinanodonta lauta* and *S. woodiana*). (Photos: Ilya V. Vikhrev [A-C], Artem A. Lubas [D], Olga V. Aksenova [E], and Aleksander V. Kondakov [F])

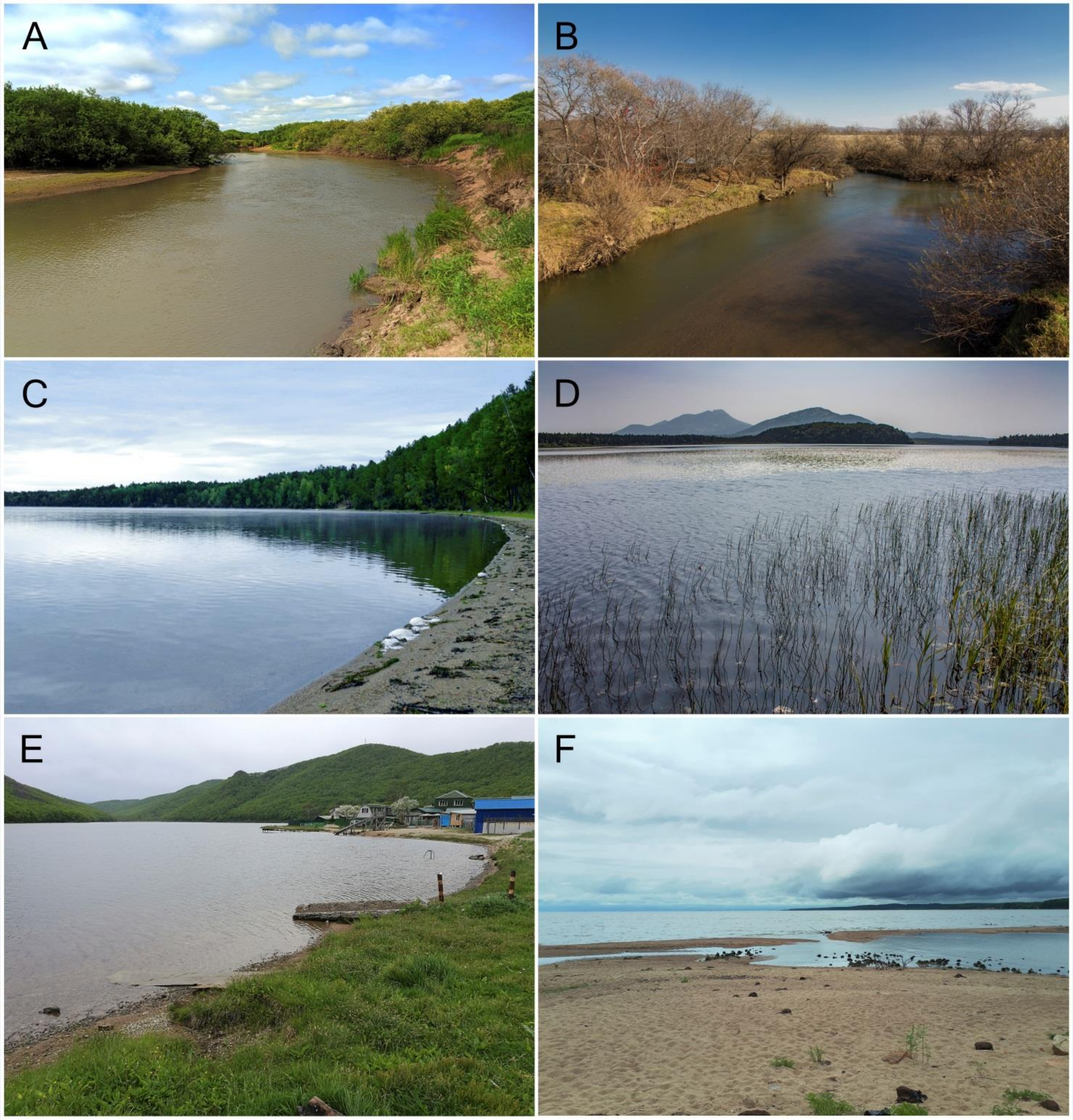

Supplementary Figure 2. Habitats of freshwater mussels (Unionidae) in the Russian Far East and Pacific Ocean islands. (**A**) Melgunovka River, Khanka Lake basin, Okhotsk Sea drainage (*Buldowskia shadini*, *Cristaria plicata*, *Nodularia douglasiae*, and *Sinanodonta schrenkii*). (**B**) Gladkaya River, Japan Sea drainage (*Buldowskia suifunica*, *Middendorffinaia mongolica*, and *Sinanodonta lauta*). (**C**) Arey Lake (*Amuranodonta kijaensis*). (**D**) Serebryannoye Lake, Kunashir Island, Pacific Ocean drainage (*Beringiana berigiana*). (**E**) Vaskovskoye Lake, Japan Sea drainage (*Beringiana berigiana*). (**F**) Khanka Lake, Okhotsk Sea drainage (*Cristaria plicata*, *Lanceolaria grayii*, *Nodularia douglasiae*, and *Sinanodonta schrenkii*). (Photos: Arthur E. Bogan [A], Ilya V. Vikhrev [B, D, E], Olga K. Klishko [C], and Aleksandr V. Kondakov [F])

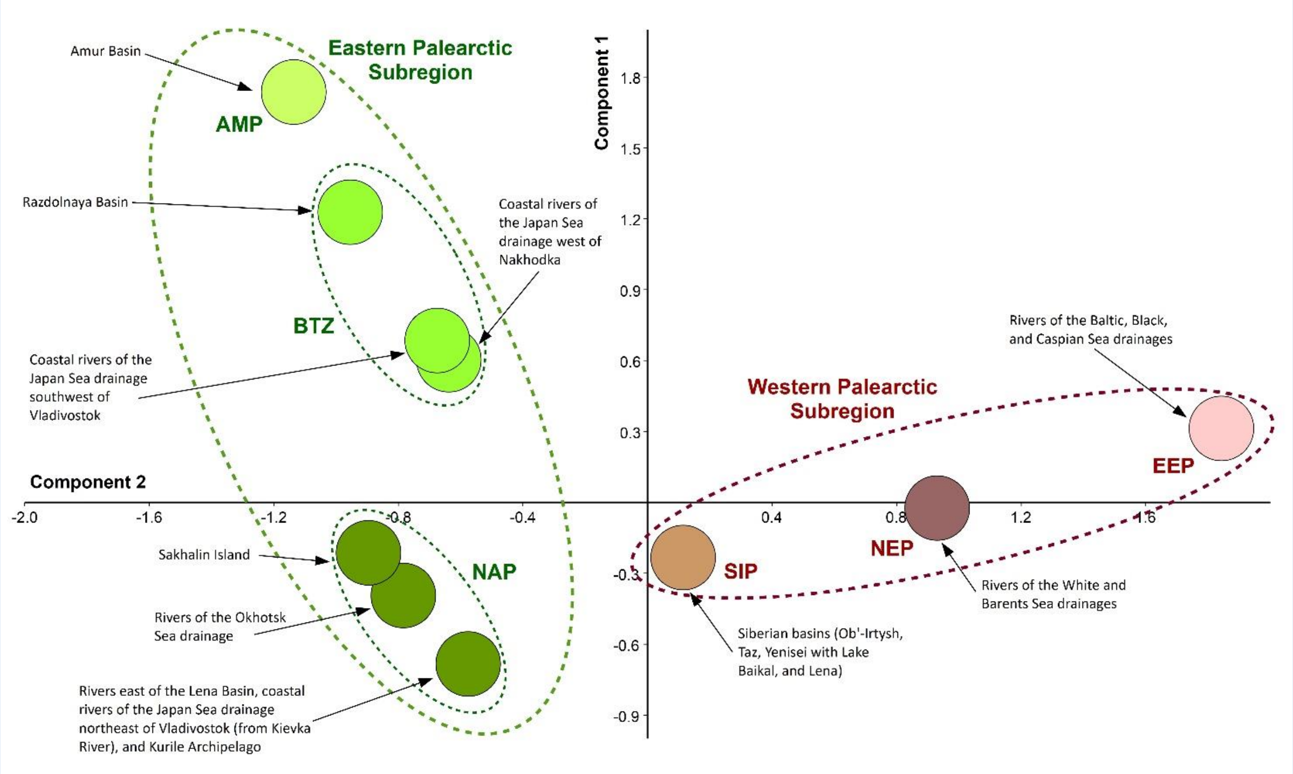

Supplementary Figure 3. Scatter plot of principal component analysis (PCA) based on the presence-absence dataset on freshwater mussels (Unionidae) throughout freshwater basins of Russia. NEP – Northern European Province, EEP – Eastern European Province, SIP – Siberian Province, NAP – North Asian Province, AMP – Amur Province, and BTZ – Amur–Korean–Japanese Biogeographic Transition Zone. The primary data on freshwater mussel distribution is presented in Dataset 1. Component 1 and component 2 accounted for 43.5% and 20.2% of the total variance, respectively.

Supplementary Table 1. List of sequences used in this study, including the species, the location and NCBI's GenBank accession numbers

Taxa	Locality	Haplotype Code	Voucher Number	*COI*	*16S rRNA*	*28S rRNA*
IN-GROUP TAXA						
**UNIONIDAE Rafinesque, 1820**						
**Unioninae Rafinesque, 1820**						
**Anodontini Rafinesque, 1820**						
***Anodonta* Lamarck, 1799**						
Anodonta anatina (Linnaeus, 1758)	Russia: European part, Nenets Autonomous Okrug, Indiga River (67.5012°N, 49.3144°E), Barents Sea drainage	AnoAna1	biv156_1	KY328448	KY328512	KY328576
A. anatina (Linnaeus, 1758)	Russia: European part, Nenets Autonomous Okrug, Indiga River (67.5012°N, 49.3144°E), Barents Sea drainage	AnoAna1	biv156_2	KY328449	KY328513	KY328577
A. anatina (Linnaeus, 1758)	Russia: European part, Nenets Autonomous Okrug, Indiga River (67.5012°N, 49.3144°E), Barents Sea drainage	AnoAna1	biv156_3	KY328450	KY328514	KY328578
A. anatina (Linnaeus, 1758)	Russia: European part, Republic of Kalmykia, Lake Lagansky Ilmen (45.3865°N, 47.3427°E), Volga Basin, Caspian Sea drainage	AnoAna2	biv157_1	KY328453	KY328517	KY328581
A. anatina (Linnaeus, 1758)	Russia: European part, Republic of Kalmykia, Lake Lagansky Ilmen (45.3865°N, 47.3427°E), Volga Basin, Caspian Sea drainage	AnoAna2	biv157_2	KY328454	KY328518	KY328582
A. anatina (Linnaeus, 1758)	Russia: Eastern Siberia, Republic of Buryatia, Lake Kiron (55.9550°N, 110.7073°E), Lake Baikal Basin, Kara Sea drainage	AnoAna2	biv158_1	KY328455	KY328519	KY328583
A. anatina (Linnaeus, 1758)	Russia: Eastern Siberia, Republic of Buryatia, Lake Kiron (55.9550°N, 110.7073°E), Lake Baikal Basin, Kara Sea drainage	AnoAna2	biv158_2	KY328456	KY328520	KY328584
A. anatina (Linnaeus, 1758)	Russia: Eastern Siberia, Republic of Buryatia, Lake Kiron (55.9550°N, 110.7073°E), Lake Baikal Basin, Kara Sea drainage	AnoAna2	biv158_3	KY328457	KY328521	KY328585
A. anatina (Linnaeus, 1758)	Russia: European part, Vologda Region, Yug River (60.4670°N, 46.5041°E), Northern Dvina Basin, White Sea drainage	AnoAna3	biv162	KY328458	KY328522	KY328586
A. anatina (Linnaeus, 1758)	Russia: European part, Arkhangelsk Region, Onega River (63.8296°N, 38.4573°E), White Sea drainage	AnoAna2	biv164_1	KY328459	KY328523	KY328587
A. anatina (Linnaeus, 1758)	Russia: European part, Arkhangelsk Region, Lake Udelnoye (63.5080°N, 41.6216°E), Northern Dvina Basin, White Sea drainage	AnoAna1	biv168_1	KY328460	KY328524	KY328588
A. anatina (Linnaeus, 1758)	Russia: Arkhangelsk Region, Lake Udelnoye (63.5080°N, 41.6216°E), Northern Dvina Basin, White Sea drainage	AnoAna1	biv168_2	KY328461	KY328525	KY328589
A. anatina (Linnaeus, 1758)	Russia: Arkhangelsk Region, Lake Udelnoye (63.5080°N, 41.6216°E), Northern Dvina Basin, White Sea drainage	AnoAna1	biv168_3	KY328462	KY328526	KY328590
A. anatina (Linnaeus, 1758)	Bulgaria: Golyama Kamchia River (43.0584°N, 26.9534°E), Kamchia Basin, Black Sea drainage	AnoAna4	biv170_1	KY328463	KY328527	KY328591
A. anatina (Linnaeus,	Bulgaria: Golyama Kamchia River	AnoAna4	biv170_2	KY328464	KY328528	KY328592

Taxa	Locality	Haplotype Code	Voucher Number	*COI*	*16S rRNA*	*28S rRNA*
1758)	(43.0584°N, 26.9534°E), Kamchia Basin, Black Sea drainage					
A. anatina (Linnaeus, 1758)	Bulgaria: Golyama Kamchia River (43.0584°N, 26.9534°E), Kamchia Basin, Black Sea drainage	AnoAna5	biv170_3	KY328465	KY328529	KY328593
A. anatina (Linnaeus, 1758)	Russia: Western Siberia, Tyumen Region, Taz River (63.5768°N, 83.8644°E), Kara Sea drainage	AnoAna2	biv173_1	KY328466	KY328530	KY328594
A. anatina (Linnaeus, 1758)	Russia: Western Siberia, Tyumen Region, Taz River (63.5768°N, 83.8644°E), Kara Sea drainage	AnoAna2	biv173_2	KY328467	KY328531	KY328595
A. anatina (Linnaeus, 1758)	Russia: Western Siberia, Tyumen Region, Taz River (63.5768°N, 83.8644°E), Kara Sea drainage	AnoAna2	biv173_3	KY328468	KY328532	KY328596
A. anatina (Linnaeus, 1758)	Russia: European part, Astrakhan Region, Volga River (45.7669°N, 47.8862°E), Caspian Sea drainage	AnoAna5	biv174_1	KY328469	KY328533	KY328597
A. anatina (Linnaeus, 1758)	Russia: Astrakhan Region, Volga River (45.7669°N, 47.8862°E), Caspian Sea drainage	AnoAna2	biv174_2	KY328470	KY328534	KY328598
A. anatina (Linnaeus, 1758)	Russia: European part, Republic of Karelia, Keret River basin (66.1592°N, 32.6471°E), White Sea drainage	AnoAna1	biv189_1	KY328471	KY328535	KY328599
A. anatina (Linnaeus, 1758)	Russia: European part, Republic of Karelia, Keret River basin (66.1592°N, 32.6471°E), White Sea drainage	AnoAna6	biv189_3	KY328472	KY328536	KY328600
A. anatina (Linnaeus, 1758)	Russia: European part, Republic of Karelia, Keret River basin (66.1592°N, 32.6471°E), White Sea drainage	AnoAna1	biv189_8	KY328473	KY328537	KY328601
A. anatina (Linnaeus, 1758)	Russia: Eastern Siberia, Republic of Buryatia, Lake Gusinoye (51.2590°N, 106.4982°E), Yenisei Basin, Kara Sea drainage	AnoAna2	biv190_2	KY328474	KY328538	KY328602
A. anatina (Linnaeus, 1758)	Russia: Eastern Siberia, Republic of Buryatia, Lake Gusinoye (51.2590°N, 106.4982°E), Yenisei Basin, Kara Sea drainage	AnoAna2	biv190_3	KY328475	KY328539	KY328603
A. anatina (Linnaeus, 1758)	Russia: Eastern Siberia, Republic of Buryatia, Lake Gusinoye (51.2590°N, 106.4982°E), Yenisei Basin, Kara Sea drainage	AnoAna2	biv190_4	KY328476	KY328540	KY328604
A. anatina (Linnaeus, 1758)	Russia: European part, Krasnodar Region, Kurchanskii Liman (45.2481°N, 37.4975°E), Azov Sea drainage	AnoAna7	biv192_1	KY328479	KY328543	KY328607
A. anatina (Linnaeus, 1758)	Russia: European part, Moscow Region, Medvezhii Lakes (55.8650°N, 38.0010°E), Volga Basin, Caspian Sea drainage	AnoAna8	biv194_4	KY328480	KY328544	KY328608
A. anatina (Linnaeus, 1758)	Russia: European part, Moscow Region, Medvezhii Lakes (55.8650°N, 38.0010°E), Volga Basin, Caspian Sea drainage	AnoAna2	biv194_5	KY328481	KY328545	KY328609
A. anatina (Linnaeus, 1758)	Russia: European part, Moscow Region, Medvezhii Lakes (55.8650°N, 38.0010°E), Volga Basin, Caspian Sea drainage	AnoAna9	biv194_7	KY328482	KY328546	KY328610
A. anatina (Linnaeus, 1758)	Russia: Western Siberia, Novosibirsk Region, Berd River (54.3206°N, 84.3938°E), Ob' Basin, Kara Sea drainage	AnoAna2	biv198_1	KY328483	KY328547	KY328611
A. anatina (Linnaeus, 1758)	Russia: Western Siberia, Novosibirsk Region, Berd River (54.3206°N, 84.3938°E), Ob' Basin, Kara Sea drainage	AnoAna2	biv198_2	KY328484	KY328548	KY328612

Taxa	Locality	Haplotype Code	Voucher Number	*COI*	*16S rRNA*	*28S rRNA*
A. anatina (Linnaeus, 1758)	Russia: Western Siberia, Novosibirsk Region, Berd River (54.3206°N, 84.3938°E), Ob' Basin, Kara Sea drainage	AnoAna2	biv198_3	KY328485	KY328549	KY328613
A. anatina (Linnaeus, 1758)	Russia: Eastern Siberia, Yakutia, Lake Syrdah (62.5689°N, 130.8861°E), Lena Basin, Laptev Sea drainage	AnoAna2	biv199_1	KY328488	KY328552	KY328616
A. anatina (Linnaeus, 1758)	Russia: Eastern Siberia, Yakutia, Lake Syrdah (62.5689°N, 130.8861°E), Lena Basin, Laptev Sea drainage	AnoAna2	biv199_2	KY328489	KY328553	KY328617
A. anatina (Linnaeus, 1758)	Russia: Eastern Siberia, Yakutia, Lake Syrdah (62.5689°N, 130.8861°E), Lena Basin, Laptev Sea drainage	AnoAna2	biv199_3	KY328490	KY328554	KY328618
A. anatina (Linnaeus, 1758)	Russia: European part, Nenets Autonomous Okrug, Lake Chekhiva, Pechora Basin (68.1305°N, 53.5269°E), Barents Sea drainage	AnoAna1	biv210_1	KY328498	KY328562	KY328626
A. anatina (Linnaeus, 1758)	Russia: European part, Nenets Autonomous Okrug, Pechora River (68.1103°N, 53.0733°E), Barents Sea drainage	AnoAna1	biv210_3	KY328499	KY328563	KY328627
A. anatina (Linnaeus, 1758)	Russia: European part, Nenets Autonomous Okrug, Sula River (67.0291°N, 51.1275°E), Pechora drainage, Barents Sea basin	AnoAna1	biv206_1	KY328493	KY328557	KY328621
A. anatina (Linnaeus, 1758)	Russia: European part, Volgograd Region, Pyatidesyatninskoye Lake (49.2421°N, 43.9255°E), Don basin, Azov Sea drainage	AnoAna10	biv208_1	KY328495	KY328559	KY328623
A. anatina (Linnaeus, 1758)	Russia: European part, Volgograd Region, Pyatidesyatninskoye Lake (49.2421°N, 43.9255°E), Don basin, Azov Sea drainage	AnoAna7	biv208_3	KY328496	KY328560	KY328624
A. anatina (Linnaeus, 1758)	Russia: European part, Moscow Region, Rezvan' Lake (54.8451°N, 37.9849°E), Oka basin, Volga Basin, Caspian Sea drainage	AnoAna2	biv209	KY328497	KY328561	KY328625
A. anatina (Linnaeus, 1758)	Russia: European part, Kursk Region, Seym River (51.7699°N, 36.2186°E), Dnieper Basin, Black Sea drainage	AnoAna11	biv277_2	MK574149	MK574286	MK574425
A. anatina (Linnaeus, 1758)	Russia: European part, Kursk Region, Seym River (51.7699°N, 36.2186°E), Dnieper Basin, Black Sea drainage	AnoAna12	biv277_3	MK574150	MK574287	MK574426
A. anatina (Linnaeus, 1758)	Russia: European part, Kursk Region, Seym River (51.7699°N, 36.2186°E), Dnieper Basin, Black Sea drainage	AnoAna1	biv277_4	MK574151	MK574288	MK574427
A. anatina (Linnaeus, 1758)	Russia: European part, Saratov Region, Khopyor River (51.5630°N, 43.1530°E), Don Basin, Azov Sea drainage	AnoAna7	biv282_2	MK574152	MK574289	MK574428
A. anatina (Linnaeus, 1758)	Russia: European part, Saratov Region, Khopyor River (51.5630°N, 43.1530°E), Don Basin, Azov Sea drainage	AnoAna13	biv282_3	MK574153	MK574290	MK574429
A. anatina (Linnaeus, 1758)	Russia: European part, Saratov Region, Khopyor River (51.5630°N, 43.1530°E), Don Basin, Azov Sea drainage	AnoAna14	biv282_4	MK574154	MK574291	MK574430
A. anatina (Linnaeus, 1758)	Russia: European part, Volgograd Region, Volga River (48.4244°N, 44.9181°E), Caspian Sea drainage	AnoAna2	biv306_2	MK574155	MK574292	MK574431
A. anatina (Linnaeus, 1758)	Russia: European part, Rostov Region, Elbuzd River (46.8762°N, 39.6995°E), Kagalnik Basin, Azov Sea drainage	AnoAna15	biv307_2	MK574156	MK574293	MK574432
A. anatina (Linnaeus, 1758)	Russia: European part, Rostov Region, Elbuzd River (46.8762°N, 39.6995°E), Kagalnik Basin, Azov Sea drainage	AnoAna16	biv307_3	MK574157	MK574294	MK574433

Taxa	Locality	Haplotype Code	Voucher Nomber	*COI*	*16S rRNA*	*28S rRNA*
A. anatina (Linnaeus, 1758)	Russia: European part, Arkhangelsk Region, Viled River (61.2785°N, 48.6681°E), Northern Dvina Basin, White Sea drainage	AnoAna17	biv616S	MK574158	n/a	n/a
A. anatina (Linnaeus, 1758)	Russia: European part, Arkhangelsk Region, Northern Dvina River (64.1414°N, 41.7653°E), White Sea drainage	AnoAna1	biv669_2	KY328502	KY328566	KY328630
A. anatina (Linnaeus, 1758)	Russia: European part, Arkhangelsk Region, Northern Dvina River (64.1414°N, 41.7653°E), White Sea drainage	AnoAna1	biv669_12	KY328503	KY328567	KY328631
A. anatina (Linnaeus, 1758)	Russia: European part, Arkhangelsk Region, Northern Dvina River (64.1414°N, 41.7653°E), White Sea drainage	AnoAna2	biv669_23	KY328504	KY328568	KY328632
A. anatina (Linnaeus, 1758)	Russia: European part, Arkhangelsk Region, Lake Pachozero (65.0007°N, 41.2693°E), Kuloi Basin, Barents Sea drainage	AnoAna18	biv670_1	KY328505	KY328569	KY328633
A. anatina (Linnaeus, 1758)	Russia: European part, Arkhangelsk Region, Lake Pachozero (65.0007°N, 41.2693°E), Kuloi Basin, Barents Sea drainage	AnoAna1	biv670_4	KY328506	KY328570	KY328634
A. anatina (Linnaeus, 1758)	Russia: European part, Arkhangelsk Region, Lake Pachozero (65.0007°N, 41.2693°E), Kuloi Basin, Barents Sea drainage	AnoAna1	biv670_7	KY328507	KY328571	KY328635
A. anatina (Linnaeus, 1758)	Russia: European part, Arkhangelsk Region, Tsiglomenka River (64.4561°N, 40.5813°E), Northern Dvina Basin, White Sea drainage	AnoAna19	1HH	KY328509	KY328573	KY328637
A. anatina (Linnaeus, 1758)	Russia: European part, Arkhangelsk Region, Tsiglomenka River (64.4561°N, 40.5813°E), Northern Dvina Basin, White Sea drainage	AnoAna1	2HH	KY328510	KY328574	KY328638
A. anatina (Linnaeus, 1758)	Russia: European part, Arkhangelsk Region, Tsiglomenka River (64.4561°N, 40.5813°E), Northern Dvina Basin, White Sea drainage	AnoAna1	4HH	KY328511	KY328575	KY328639
A. anatina (Linnaeus, 1758)	Russia: European part, Arkhangelsk Region, Onega Peninsula, Lopshenga River (64.9542°N, 37.5186°E), White Sea drainage	AnoAna1	biv552_1	MK574159	MK574295	MK574434
A. anatina (Linnaeus, 1758)	Russia: European part, Arkhangelsk Region, Onega Peninsula, Solenoye Lake (64.8767°N, 38.9809°E), White Sea drainage	AnoAna20	biv553_1	MK574160	MK574296	MK574435
A. anatina (Linnaeus, 1758)	Russia: European part, Arkhangelsk Region, Onega Peninsula, Solenoye Lake (64.5774°N, 37.9460°E), White Sea drainage	AnoAna1	biv553_2	MK574161	MK574297	MK574436
A. anatina (Linnaeus, 1758)	Russia: European part, Republic of Adygea, Kuban River (44.9497°N, 39.3544°E), Azov Sea drainage	AnoAna21	biv554_1	MK574162	MK574298	MK574437
A. anatina (Linnaeus, 1758)	Russia: European part, Republic of Adygea, Kuban River (44.9497°N, 39.3544°E), Azov Sea drainage	AnoAna22	biv554_2	MK574163	n/a	n/a
A. anatina (Linnaeus, 1758)	Russia: European part, Republic of Adygea, Kuban River (44.9497°N, 39.3544°E), Azov Sea drainage	AnoAna23	biv554_3	MK574164	MK574299	MK574438
A. anatina (Linnaeus, 1758)	Russia: European part, Krasnodar Region, Kirpili River (45.3539°N, 39.8053°E), Azov Sea drainage	AnoAna24	biv557_1	MK574165	MK574300	MK574439
A. anatina (Linnaeus, 1758)	Russia: European part, Krasnodar Region, Kirpili River (45.3539°N, 39.8053°E), Azov Sea drainage	AnoAna25	biv557_2	MK574166	n/a	n/a
A. anatina (Linnaeus,	Russia: European part, Krasnodar	AnoAna25	biv557_3	MK574167	n/a	n/a

Taxa	Locality	Haplotype Code	Voucher Number	*COI*	*16S rRNA*	*28S rRNA*
1758)	Region, Kirpili River (45.3539°N, 39.8053°E), Azov Sea drainage					
A. anatina (Linnaeus, 1758)	Russia: European part, Krasnodar Region, Kirpili River (45.3539°N, 39.8053°E), Azov Sea drainage	AnoAna25	biv558	MK574168	n/a	n/a
A. anatina (Linnaeus, 1758)	Russia: European part, Rostov Region, Staryi Donets Lake (48.3406°N, 40.1096°E), Don Basin, Azov Sea drainage	AnoAna7	biv560_1	MK574169	MK574301	MK574440
A. anatina (Linnaeus, 1758)	Russia: European part, Rostov Region, Staryi Donets Lake (48.3406°N, 40.1096°E), Don Basin, Azov Sea drainage	AnoAna22	biv560_2	MK574170	n/a	n/a
A. anatina (Linnaeus, 1758)	Russia: European part, Rostov Region, Staryi Donets Lake (48.3406°N, 40.1096°E), Don Basin, Azov Sea drainage	AnoAna26	biv560_3	MK574171	n/a	n/a
A. anatina (Linnaeus, 1758)	Russia: European part, Rostov Region, Sokolovskoye Reservoir (47.8438°N, 39.9030°E), Don Basin, Azov Sea drainage	AnoAna27	biv563	MK574172	n/a	n/a
A. anatina (Linnaeus, 1758)	Russia: European part, Rostov Region, Aksai River (47.3582°N, 40.0632°E), Don Basin, Azov Sea drainage	AnoAna28	biv565_1	MK574173	MK574302	MK574441
A. anatina (Linnaeus, 1758)	Russia: European part, Rostov Region, Aksai River (47.3582°N, 40.0632°E), Don Basin, Azov Sea drainage	AnoAna29	biv565_2	MK574174	n/a	n/a
A. anatina (Linnaeus, 1758)	Russia: European part, Rostov Region, Aksai River (47.3582°N, 40.0632°E), Don Basin, Azov Sea drainage	AnoAna30	biv565_3	MK574175	n/a	n/a
A. anatina (Linnaeus, 1758)	Russia: European part, Krasnodar Region, Kuban River (45.2655°N, 37.3708°E), Azov Sea drainage	AnoAna31	biv569_1	MK574176	MK574303	MK574442
A. anatina (Linnaeus, 1758)	Russia: European part, Krasnodar Region, Kuban River (45.2655°N, 37.3708°E), Azov Sea drainage	AnoAna32	biv569_2	MK574177	n/a	n/a
A. anatina (Linnaeus, 1758)	Russia: European part, Krasnodar Region, Kuban River (45.2655°N, 37.3708°E), Azov Sea drainage	AnoAna33	biv569_3	MK574178	n/a	n/a
A. anatina (Linnaeus, 1758)	Russia: European part, Ryazan Region, Oka River (54.6603°N, 39.8371°E), Volga Basin, Caspian Sea drainage	AnoAna34	biv571_1	MK574179	MK574304	MK574443
A. anatina (Linnaeus, 1758)	Russia: European part, Ryazan Region, Oka River (54.6603°N, 39.8371°E), Volga Basin, Caspian Sea drainage	AnoAna17	biv572_3	MK574180	n/a	n/a
A. anatina (Linnaeus, 1758)	Russia: European part, Ryazan Region, Oka River (54.6603°N, 39.8371°E), Volga Basin, Caspian Sea drainage	AnoAna35	biv573_4	MK574181	n/a	n/a
A. anatina (Linnaeus, 1758)	Kazakhstan: Ural River (50.7131°N, 51.3808 °E), Caspian Sea drainage	AnoAna36	biv593_1	MK603925	n/a	n/a
A. anatina (Linnaeus, 1758)	Kazakhstan: Ural River (50.7131°N, 51.3808 °E), Caspian Sea drainage	AnoAna35	biv593_2	MK603926	n/a	n/a
A. anatina (Linnaeus, 1758)	Kazakhstan: Ural River (50.7131°N, 51.3808 °E), Caspian Sea drainage	AnoAna35	biv593_3	MK603927	n/a	n/a
A. anatina (Linnaeus, 1758)	Kazakhstan: Ural River (50.7131°N, 51.3808 °E), Caspian Sea drainage	AnoAna35	biv593_4	MK603928	n/a	n/a
A. anatina (Linnaeus, 1758)	Kazakhstan: Ural River (50.7131°N, 51.3808 °E), Caspian Sea drainage	AnoAna35	biv593_5	MK603929	n/a	n/a
A. anatina (Linnaeus, 1758)	Kazakhstan: Ural River (50.7131°N, 51.3808 °E), Caspian Sea drainage	AnoAna35	biv593_6	MK603930	n/a	n/a
A. anatina (Linnaeus, 1758)	Ukraine: Vorskla River, Dnieper Basin, Black Sea drainage	AnoAna37	BIV0199	MH062759	n/a	n/a
A. anatina (Linnaeus, 1758)	Ukraine: Murafa River, Dniester Basin, Black Sea drainage	AnoAna38	BIV0201	MH062760	n/a	n/a

Taxa	Locality	Haplotype Code	Voucher Number	*COI*	*16S rRNA*	*28S rRNA*
A. anatina (Linnaeus, 1758)	Ukraine: Revna River, Dnieper Basin, Black Sea drainage	AnoAna38	BIV0202	MH062761	n/a	n/a
A. anatina (Linnaeus, 1758)	Ukraine: Pond in Dinotzky	AnoAna39	BIV0204	MH062762	n/a	n/a
A. anatina (Linnaeus, 1758)	Ukraine: Suchoy Tashlyk River, Southern Bug Basin, Black Sea drainage	AnoAna39	BIV0205	MH062763	n/a	n/a
A. anatina (Linnaeus, 1758)	Ukraine: Revna River, Dnieper Basin, Black Sea drainage	AnoAna38	BIV0206	MH062764	n/a	n/a
A. anatina (Linnaeus, 1758)	Russia: Eastern Siberia, Transbaikalia, Lake Tasey (52.2921°N, 113.0898°E), Lena Basin, Laptev Sea drainage	AnoAna40	BIV0256	MH062765	n/a	n/a
A. anatina (Linnaeus, 1758)	Russia: Eastern Siberia, Republic of Buryatia, Lake Shchuchye (51.4176°N, 106.5433°E), Yenisei Basin, Kara Sea drainage	AnoAna40	BIV3378	MH062769	n/a	n/a
A. anatina (Linnaeus, 1758)	Russia: Eastern Siberia, Republic of Buryatia, Lake Shchuchye (51.4176°N, 106.5433°E), Yenisei Basin, Kara Sea drainage	AnoAna40	BIV3379	MH062770	n/a	n/a
A. anatina (Linnaeus, 1758)	Russia: Eastern Siberia, Republic of Buryatia, Lake Shchuchye (51.4176°N, 106.5433°E), Yenisei Basin, Kara Sea drainage	AnoAna40	BIV3381	MH062771	n/a	n/a
A. anatina (Linnaeus, 1758)	Russia: Eastern Siberia, Republic of Buryatia, Lake Torma (51.5600°N, 107.0090°E)	AnoAna40	BIV3382	MH062772	n/a	n/a
A. anatina (Linnaeus, 1758)	Russia: Eastern Siberia, Republic of Buryatia, Lake Torma (51.5600°N, 107.0090°E)	AnoAna40	BIV3383	MH062773	n/a	n/a
A. anatina (Linnaeus, 1758)	Russia: Eastern Siberia, Republic of Buryatia, Lake Torma (51.5600°N, 107.0090°E)	AnoAna40	BIV3384	MH062774	n/a	n/a
A. anatina (Linnaeus, 1758)	Russia: Eastern Siberia, Selenga River (51.8473 °N, 107.5583°E), Yenisei Basin, Kara Sea drainage	AnoAna40	BIV3387	MH062776	n/a	n/a
A. anatina (Linnaeus, 1758)	Russia: Eastern Siberia, Selenga River (51.8473 °N, 107.5583°E), Yenisei Basin, Kara Sea drainage	AnoAna40	BIV3388	MH062777	n/a	n/a
A. anatina (Linnaeus, 1758)	Russia: Eastern Siberia, Republic of Buryatia, Lake Bolshoye Eravnoye (52.5972°N, 111.5001°E), Lena Basin, Laptev Sea drainage	AnoAna41	BIV3392	MH062778	n/a	n/a
A. anatina (Linnaeus, 1758)	Russia: Eastern Siberia, Republic of Buryatia, Lake Bolshoye Eravnoye (52.5972°N, 111.5001°E), Lena Basin, Laptev Sea drainage	AnoAna40	BIV3393	MH062779	n/a	n/a
A. anatina (Linnaeus, 1758)	Russia: Eastern Siberia, Republic of Buryatia, Lake Bolshoye Eravnoye (52.5972°N, 111.5001°E), Lena Basin, Laptev Sea drainage	AnoAna42	BIV3394	MH062780	n/a	n/a
A. anatina (Linnaeus, 1758)	Russia: Eastern Siberia, Lake Baikal (52.1555°N, 106.2637°E), Yenisei Basin, Kara Sea drainage	AnoAna40	BIV3401	MH062784	n/a	n/a
A. anatina (Linnaeus, 1758)	Russia: Eastern Siberia, Lake Baikal (52.1555°N, 106.2637°E), Yenisei Basin, Kara Sea drainage	AnoAna43	BIV3402	MH062785	n/a	n/a
A. anatina (Linnaeus, 1758)	Russia: Eastern Siberia, Lake Baikal (52.1555°N, 106.2637°E), Yenisei Basin, Kara Sea drainage	AnoAna40	BIV3405	MH062786	n/a	n/a
A. cygnea (Linnaeus, 1758)	Russia: European part, Moscow Region, Medvezhii Lakes (55.8650°N, 38.0010°E), Volga Basin, Caspian Sea drainage	AnoCyg1	biv194_1	MK034153	MK574305	MK574444
A. cygnea (Linnaeus, 1758)	Russia: European part, Moscow Region, Medvezhii Lakes (55.8650°N, 38.0010°E), Volga Basin, Caspian Sea	AnoCyg1	biv194_2	MK034154	MK574306	MK574445

Taxa	Locality	Haplotype Code	Voucher Number	*COI*	*16S rRNA*	*28S rRNA*
	drainage					
A. cygnea (Linnaeus, 1758)	Russia: European part, Kaliningrad Region, Lebedinoye Lake (54.7938°N, 20.5286°E), Baltic Sea drainage	AnoCyg1	biv207_1	MK034159	MK574311	MK574450
A. cygnea (Linnaeus, 1758)	Russia: European part, Kaliningrad Region, Lebedinoye Lake (54.7938°N, 20.5286°E), Baltic Sea drainage	AnoCyg1	biv207_2	MK574184	MK574312	MK574451
A. cygnea (Linnaeus, 1758)	Russia: European part, Kaliningrad Region, Lebedinoye Lake (54.7938°N, 20.5286°E), Baltic Sea drainage	AnoCyg1	biv207_3	MK034160	MK574313	MK574452
A. cygnea (Linnaeus, 1758)	Russia: European part, Moscow Region, Lake Glubokoye (55.7519°N, 36.5102°E), Volga Basin, Caspian Sea drainage	AnoCyg2	biv204_1	MK034157	MK574307	MK574446
A. cygnea (Linnaeus, 1758)	Russia: European part, Moscow Region, Lake Glubokoye (55.7519°N, 36.5102°E), Volga Basin, Caspian Sea drainage	AnoCyg2	biv204_3	MK034158	MK574308	MK574447
A. cygnea (Linnaeus, 1758)	Russia: European part, Moscow Region, Lake Glubokoye (55.7519°N, 36.5102°E), Volga Basin, Caspian Sea drainage	AnoCyg2	biv204_6	MK574182	MK574309	MK574448
A. cygnea (Linnaeus, 1758)	Russia: European part, Moscow Region, Lake Glubokoye (55.7519°N, 36.5102°E), Volga Basin, Caspian Sea drainage	AnoCyg2	biv204_7	MK574183	MK574310	MK574449
***Pseudanodonta* Bourguignat, 1876**						
Pseudanodonta complanata (Rossmässler, 1835)	Russia: European part, Saratov Region, Khopyor River (51.4878°N, 42.5931°E), Don Basin, Azov Sea drainage	PseCom1	biv195_1	MK034155	MK574314	MK574453
P. complanata (Rossmässler, 1835)	Russia: European part, Saratov Region, Khopyor River (51.4878°N, 42.5931°E), Don Basin, Azov Sea drainage	PseCom2	biv195_2	MK034156	MK574315	MK574454
P. complanata (Rossmässler, 1835)	Russia: European part, Saratov Region, Khopyor River (51.4878°N, 42.5931°E), Don Basin, Azov Sea drainage	PseCom3	biv195_3	MK574185	MK574316	MK574455
P. complanata (Rossmässler, 1835)	Russia: European part, Ryazan Region, Oka River (54.4620°N, 40.5771°E), Volga Basin, Caspian Sea drainage	PseCom4	biv574_1	MK574186	MK574317	MK574456
P. complanata (Rossmässler, 1835)	Russia: European part, Ryazan Region, Oka River (54.4620°N, 40.5771°E), Volga Basin, Caspian Sea drainage	PseCom4	biv574_3	MK574188	MK574319	MK574458
P. complanata (Rossmässler, 1835)	Russia: European part, Ryazan Region, Oka River (54.4620°N, 40.5771°E), Volga Basin, Caspian Sea drainage	PseCom5	biv574_2	MK574187	MK574318	MK574457
**Cristariini Lopes-Lima et al., 2017**						
***Amuranodonta* Moskvicheva, 1973**						
Amuranodonta kijaensis Moskvicheva, 1973	Russia: Eastern Siberia, Transbaikalia, Arey Lake (50.9820°N, 111.2378°E)	AneKij1	BIV0228	MK574204	MK574334	MK574473
A. kijaensis Moskvicheva, 1973	Russia: Eastern Siberia, Transbaikalia, Arey Lake (50.9820°N, 111.2378°E)	AneKij2	BIV0229	MK574205	MK574335	MK574474
***Anemina* Haas, 1969**						
Anemina arcaeformis (Heude, 1877)*	China	AneArc1	n/a	NC_026674	NC_026674	MG595457*
A. arcaeformis (Heude, 1877)	China: Jiangxi, Qinglan Lake	AneArc2	16_NCU_ XPWU_SU024	MG462934	n/a	MG595461

Taxa	Locality	Haplotype Code	Voucher Number	*COI*	*16S rRNA*	*28S rRNA*
A. arcaeformis (Heude, 1877)	China: Jiangxi, Niutoushan of Poyang Lake	AneArc3	16_NCU_ XPWU_SU026	MG462936	n/a	MG595463
Beringiana **Starobogatov in Zatravkin, 1983**						
Beringiana beringiana (Middendorff, 1851)	Russia: Far East, Kamchatka Region, Bolshaya River (approx. 52.769°N, 156.192°E), Okhotsk Sea drainage	BerBer1	biv169	MK034152	MK574336	MK574475
B. beringiana (Middendorff, 1851)	Russia: Eastern Siberia, Yakutia, a small lake near airport (68.5584°N, 146.2399°E), Indigirka Basin, East Siberian Sea drainage	BerBer1	biv302_1	MK574214	MK574345	MK574484
B. beringiana (Middendorff, 1851)	Russia: Eastern Siberia, Yakutia, a small lake near airport (68.5584°N, 146.2399°E), Indigirka Basin, East Siberian Sea drainage	BerBer1	biv302_10	MK574215	MK574346	MK574485
B. beringiana (Middendorff, 1851)	Russia: Eastern Siberia, Yakutia, a small lake near airport (68.5584°N, 146.2399°E), Indigirka Basin, East Siberian Sea drainage	BerBer1	biv302_11	MK574216	MK574347	MK574486
B. beringiana (Middendorff, 1851)	USA: Alaska, Anchorage, Waldron Lake (61.1788°N, 149.8522°W)	BerBer1	biv_283_1	MK574218	MK574348	MK574487
B. beringiana (Middendorff, 1851)	USA: Alaska, Anchorage, Waldron Lake (61.1788°N, 149.8522°W)	BerBer1	biv_283_11	MK574219	MK574349	MK574488
B. beringiana (Middendorff, 1851)	USA: Alaska, Anchorage, Waldron Lake (61.1788°N, 149.8522°W)	BerBer1	biv_283_12	MK574220	MK574350	MK574489
B. beringiana (Middendorff, 1851)	USA: Alaska, Equmen Lake (60.5276°N, 150.4387°W)	BerBer1	biv_284_7	MK574223	MK574353	MK574492
B. beringiana (Middendorff, 1851)	USA: Alaska, Birch Lake (64.3046°N, 146.6659°W)	BerBer1	biv_286_1	MK574224	MK574354	MK574493
B. beringiana (Middendorff, 1851)	USA: Alaska, Birch Lake (64.3046°N, 146.6659°W)	BerBer1	biv_286_4	MK574226	MK574355	MK574494
B. beringiana (Middendorff, 1851)	Russia: Far East, Kurile Archipelago, Kunashir Island (44.0463°N, 145.7399°E)	BerBer2	biv_186_2	MK574206	MK574337	MK574476
B. beringiana (Middendorff, 1851)	Russia: Far East, Kurile Archipelago, Kunashir Island (44.0463°N, 145.7399°E)	BerBer2	biv_186_4	MK574207	MK574338	MK574477
B. beringiana (Middendorff, 1851)	Russia: Far East, Primorye Region, Avakumovka River (43.7578°N, 135.1865°E), Japan Sea drainage	BerBer3	biv272_6	MK574208	MK574339	MK574478
B. beringiana (Middendorff, 1851)	Russia: Far East, Primorye Region, Avakumovka River (43.7578°N, 135.1865°E), Japan Sea drainage	BerBer3	biv272_9	MK574209	MK574340	MK574479
B. beringiana (Middendorff, 1851)	Russia: Far East, Primorye Region, Vaskovskoye Lake (44.3468°N, 135.8235°E), Japan Sea drainage	BerBer3	biv273_2	MK574210	MK574341	MK574480
B. beringiana (Middendorff, 1851)	Russia: Far East, Primorye Region, Vaskovskoye Lake (44.3468°N, 135.8235°E), Japan Sea drainage	BerBer3	biv273_8	MK574211	MK574342	MK574481
B. beringiana (Middendorff, 1851)	Russia: Far East, Primorye Region, Yaponskoye Lake (45.0422°N, 136.6692°E), Japan Sea drainage	BerBer4	biv274_3	MK574212	MK574343	MK574482
B. beringiana (Middendorff, 1851)	Russia: Far East, Primorye Region, Yaponskoye Lake (45.0422°N, 136.6692°E), Japan Sea drainage	BerBer4	biv274_6	MK574213	MK574344	MK574483
B. beringiana (Middendorff, 1851)	Russia: Far East, Sakhalin Island	BerBer5	BIV1526	MK574217	n/a	n/a
B. beringiana (Middendorff, 1851)	USA: Alaska, Equmen Lake (60.5276°N, 150.4387°W)	BerBer6	biv_284_2	MK574221	MK574351	MK574490
B. beringiana (Middendorff, 1851)	USA: Alaska, Equmen Lake (60.5276°N, 150.4387°W)	BerBer7	biv_284_3	MK574222	MK574352	MK574491
B. beringiana (Middendorff, 1851)	USA: Alaska, Birch Lake (64.3046°N, 146.6659°W)	BerBer8	biv_286_2	MK574225	n/a	n/a
Buldowskia **Moskvicheva, 1973**						
Buldowskia suifunica (Lindholm, 1925)	Russia: Far East, Primorye Region, Gladkaya River (42.7065°N,	BulSu1	Biv225_5	MK574189	MK574320	MK574459

Taxa	Locality	Haplotype Code	Voucher Number	*COI*	*16S rRNA*	*28S rRNA*
	130.9084°E), Japan Sea drainage					
B. suifunica (Lindholm, 1925)	Russia: Far East, Primorye Region, Gladkaya River (42.7065°N, 130.9084°E), Japan Sea drainage	BulSu2	Biv225_11	MK574190	MK574321	MK574460
B. suifunica (Lindholm, 1925)	Russia: Far East, Primorye Region, Gladkaya River (42.7065°N, 130.9084°E), Japan Sea drainage	BulSu3	Biv225_14	MK574191	MK574322	MK574461
B. suifunica (Lindholm, 1925)	Russia: Far East, Primorye Region, Gladkaya River (42.7065°N, 130.9084°E), Japan Sea drainage	BulSu3	Biv225_17	MK574192	MK574323	MK574462
B. suifunica (Lindholm, 1925)	Russia: Far East, Primorye Region, Soldatskoye Lake (43.7747°N, 131.9406°E), Razdolnaya Basin, Japan Sea drainage	BulSu4	biv227_3	MK574193	MK574324	MK574463
B. suifunica (Lindholm, 1925)	Russia: Far East, Primorye Region, Soldatskoye Lake (43.7747°N, 131.9406°E), Razdolnaya Basin, Japan Sea drainage	BulSu5	biv227_10	MK574194	MK574325	MK574464
B. shadini (Moskvicheva, 1973)	Russia: Far East, Primorye Region, Blagodatnoye Lake (44.3260°N, 132.0717°E), Ussuri Basin, Okhotsk Sea drainage	BulSha1	biv228_5	MK574195	MK574326	MK574465
B. shadini (Moskvicheva, 1973)	Russia: Far East, Primorye Region, Blagodatnoye Lake (44.3260°N, 132.0717°E), Ussuri Basin, Okhotsk Sea drainage	BulSha1	biv228_7	MK574196	MK574327	MK574466
B. shadini (Moskvicheva, 1973)	Russia: Far East, Primorye Region, Blagodatnoye Lake (44.3260°N, 132.0717°E), Ussuri Basin, Okhotsk Sea drainage	BulSha2	biv228_16	MK574197	MK574328	MK574467
B. shadini (Moskvicheva, 1973)	Russia: Far East, Primorye Region, Melgunovka River (44.5939°N, 132.1818°E), Khanka Lake basin, Okhotsk Sea drainage	BulSha3	biv_497_1	MK574198	MK574329	MK574468
B. shadini (Moskvicheva, 1973)	Russia: Far East, Primorye Region, Melgunovka River (44.5939°N, 132.1818°E), Khanka Lake basin, Okhotsk Sea drainage	BulSha3	biv_497_3	MK574200	MK574330	MK574469
B. shadini (Moskvicheva, 1973)	Russia: Far East, Primorye Region, Melgunovka River (44.5939°N, 132.1818°E), Khanka Lake basin, Okhotsk Sea drainage	BulSha4	biv_497_2	MK574199	n/a	n/a
B. shadini (Moskvicheva, 1973)	Russia: Eastern Siberia, Transbaikalia, Onon River (50.5381°N, 115.1216°E), Amur Basin, Okhotsk Sea drainage	BulSha5	biv319	MK574201	MK574331	MK574470
B. shadini (Moskvicheva, 1973)	Russia: Eastern Siberia, Transbaikalia, Onon River (50.5381°N, 115.1216°E), Amur Basin, Okhotsk Sea drainage	BulSha5	biv319_2	MK574202	MK574332	MK574471
B. shadini (Moskvicheva, 1973)	Russia: Eastern Siberia, Transbaikalia, Onon River (50.5381°N, 115.1216°E), Amur Basin, Okhotsk Sea drainage	BulSha5	biv319_3	MK574203	MK574333	MK574472
***Cristaria* Schumacher, 1817**						
Cristaria plicata (Leach, 1814)	Vietnam	CriPli1	90_1	KY561634	MK574375	KY561666
C. plicata (Leach, 1814)	Vietnam	CriPli1	90_2	MK574239	MK574376	MK574516
C. plicata (Leach, 1814)	Vietnam	CriPli1	90_3	MK574240	MK574377	MK574517
C. plicata (Leach, 1814)	Russia: Far East, Primorye Region, Amur River (140.7014°N, 53.1319°E), Okhotsk Sea drainage	CriPli2	biv133_2	MK574241	n/a	MK574518
C. plicata (Leach, 1814)	Russia: Far East, Primorye Region, Khanka Lake (44.7075°N, 132.0739°E), Okhotsk Sea drainage	CriPli3	biv276_2	MK574242	MK574378	MK574519

Taxa	Locality	Haplotype Code	Voucher Number	*COI*	*16S rRNA*	*28S rRNA*
C. plicata (Leach, 1814)	Russia: Far East, Primorye Region, Khanka Lake (44.7075°N, 132.0739°E), Okhotsk Sea drainage	CriPli4	biv495_23	MK574243	MK574379	MK574520
C. plicata (Leach, 1814)	Russia: Far East, Primorye Region, Khanka Lake (44.7075°N, 132.0739°E), Okhotsk Sea drainage	CriPli5	biv495_27	MK574244	MK574380	MK574521
C. plicata (Leach, 1814)	Russia: Far East, Primorye Region, Khanka Lake (44.7075°N, 132.0739°E), Okhotsk Sea drainage	CriPli6	biv495_28	MK574245	MK574381	MK574522
C. plicata (Leach, 1814)	Russia: Far East, Primorye Region, Luchegorsky Reservoir (46.4679°N, 134.3288°E), Ussuri Basin, Okhotsk Sea drainage	CriPli7	Biv1530	KT348507	n/a	n/a
C. plicata (Leach, 1814)	Russia: Far East, Primorye Region, Luchegorsky Reservoir (46.4679°N, 134.3288°E), Ussuri Basin, Okhotsk Sea drainage	CriPli8	Biv1531	KT348508	n/a	n/a
C. plicata (Leach, 1814)	Russia: Far East, Primorye Region, Khanka Lake (44.7423°N, 132.0787°E), Okhotsk Sea drainage	CriPli9	Biv1537a	KU297678	n/a	n/a
C. plicata (Leach, 1814)	China: Lower Yangtze	CriPli10	C.P CH1	EU698893	n/a	n/a
C. plicata (Leach, 1814)	China: Lower Yangtze	CriPli11	C.P CH8	EU698897	n/a	n/a
C. plicata (Leach, 1814)	China	CriPli12	AS13MT02	JF700153	n/a	n/a
C. plicata (Leach, 1814)	China: Zhejiang	CriPli13	n/a	FJ986302	n/a	n/a
C. plicata (Leach, 1814)	Russia: Eastern Siberia, Transbaikalia, Onon River (50.5192°N, 115.1342°E), Amur Basin, Okhotsk Sea drainage	CriPli14	BIV0246	KT362704	MK574382	MK574523
C. plicata (Leach, 1814)	Russia: Eastern Siberia, Transbaikalia, Onon River (50.5192°N, 115.1342°E), Amur Basin, Okhotsk Sea drainage	CriPli15	BIV0247	KT362705	MK574383	MK574524
***Sinanodonta* Modell, 1945**						
Sinanodonta lauta (Martens, 1877)	Russia: Eastern Siberia, Krasnoyarsk, Yenisei River (55.9892°N, 92.8700°E) (non-native population)	SinLau1	biv191_1	KY561633	KY561648	KY561665
S. lauta (Martens, 1877)	Russia: Eastern Siberia, Krasnoyarsk, Yenisei River (55.9892°N, 92.8700°E) (non-native population)	SinLau1	biv191_4	KY978737	MK574356	MK574495
S. lauta (Martens, 1877)	Russia: Eastern Siberia, Krasnoyarsk, Yenisei River (55.9892°N, 92.8700°E) (non-native population)	SinLau1	biv191_6	KY978739	MK574357	MK574496
S. lauta (Martens, 1877)	Russia: Eastern Siberia, Krasnoyarsk, Yenisei River (55.9892°N, 92.8700°E) (non-native population)	SinLau1	biv191_7	KY978740	MK574358	MK574497
S. lauta (Martens, 1877)	Russia: Eastern Siberia, Krasnoyarsk, Yenisei River (55.9892°N, 92.8700°E) (non-native population)	SinLau1	biv191_9	KY978741	MK574359	MK574498
S. lauta (Martens, 1877)	Russia: Far East, Primorye Region, Gladkaya River (42.7065°N, 130.9084°E), Japan Sea drainage	SinLau2	biv225_1	KY978742	MK574360	MK574499
S. lauta (Martens, 1877)	Russia: Far East, Primorye Region, Gladkaya River (42.7065°N, 130.9084°E), Japan Sea drainage	SinLau2	biv225_2	KY978743	MK574361	MK574500
S. lauta (Martens, 1877)	South Korea	SinLau3	isolate C82	GQ451870	n/a	n/a
S. lauta (Martens, 1877)	South Korea	SinLau4	isolate C8	GQ451869	n/a	n/a
S. lauta (Martens, 1877)	Japan	SinLau5	n/a	AB055627	n/a	n/a
S. schrenkii (Lea, 1870)	Russia: Far East, Primorye Region, Razdolnaya River, Japan Sea drainage	SinSki1	voucher 348	KU853266	n/a	n/a
S. schrenkii (Lea, 1870)	Russia: Far East, Primorye Region, Amur River, Okhotsk Sea drainage	SinSki2	voucher 349	KU853267	n/a	n/a
S. schrenkii (Lea, 1870)	Russia: Far East, Primorye Region, Khanka Lake, Okhotsk Sea drainage	SinSki2	voucher 384	KU853268	n/a	n/a

Taxa	Locality	Haplotype Code	Voucher Number	*COI*	*16S rRNA*	*28S rRNA*
S. schrenkii (Lea, 1870)	Russia: Far East, Primorye Region, Khanka Lake, Okhotsk Sea drainage	SinSki1	voucher 395	KU853269	n/a	n/a
S. schrenkii (Lea, 1870)	Russia: Far East, Primorye Region, Melgunovka River (44.5939°N, 132.1818°E), Khanka Lake basin, Okhotsk Sea drainage	SinSki3	biv496_1	MK574232	MK574362	MK574503
S. schrenkii (Lea, 1870)	Russia: Far East, Primorye Region, Melgunovka River (44.5939°N, 132.1818°E), Khanka Lake basin, Okhotsk Sea drainage	SinSki4	biv496_2	MK574233	MK574363	MK574504
S. schrenkii (Lea, 1870)	Russia: Far East, Primorye Region, Melgunovka River (44.5939°N, 132.1818°E), Khanka Lake basin, Okhotsk Sea drainage	SinSki1	biv496_3	MK574234	n/a	n/a
S. schrenkii (Lea, 1870)	Russia: Far East, Primorye Region, Khanka Lake (44.8255°N, 132.0456°E), Ussuri Basin, Okhotsk Sea drainage	SinSki5	biv501_1	MK574235	MK574364	MK574505
S. schrenkii (Lea, 1870)	Russia: Eastern Siberia, Transbaikalia, Ingoda River (51.9570°N, 113.5991°E), Amur Basin, Okhotsk Sea drainage	SinSki6	BIV0248	MK574236	MK574365	MK574506
S. schrenkii (Lea, 1870)	Russia: Eastern Siberia, Transbaikalia, Ingoda River (51.9570°N, 113.5991°E), Amur Basin, Okhotsk Sea drainage	SinSki7	BIV0249	MK574237	MK574366	MK574507
S. schrenkii (Lea, 1870)	Russia: Far East, Primorye Region, Ilistaya River, Khanka Lake basin, Okhotsk Sea drainage	SinSki2	BIV1528	MK574238	n/a	n/a
S. woodiana (Lea, 1834)	Myanmar: Irrawaddy River basin, a floodplain lake near Bhamo (24.2830°N, 97.2586°E) (non-native population)	SinWod1	biv 269	MF497809	MK574367	MK574508
S. woodiana (Lea, 1834)	Myanmar: Irrawaddy River basin, a floodplain lake near Bhamo (24.2830°N, 97.2586°E) (non-native population)	SinWod1	biv269_15	MF497807	MK574368	MK574509
S. woodiana (Lea, 1834)	Myanmar: Irrawaddy River basin, a floodplain lake near Bhamo (24.2830°N, 97.2586°E) (non-native population)	SinWod1	biv269_16	MF497808	MK574369	MK574510
S. woodiana (Lea, 1834)	Russia: Eastern Siberia, Krasnoyarsk Region, Yenisei River (55.9892°N, 92.8700°E) (non-native population)	SinWod1	biv 191_2	KY978735	MK574370	MK574511
S. woodiana (Lea, 1834)	Russia: Eastern Siberia, Krasnoyarsk Region, Yenisei River (55.9892°N, 92.8700°E) (non-native population)	SinWod1	biv 191_3	KY978736	MK574371	MK574512
S. woodiana (Lea, 1834)	Russia: Eastern Siberia, Krasnoyarsk Region, Yenisei River (55.9892°N, 92.8700°E) (non-native population)	SinWod1	biv 191_5	KY978738	MK574372	MK574513
S. woodiana (Lea, 1834)	Uzbekistan: Khorezm Region, Amu Darya River (41.5358°N, 60.8864°E), Aral Sea drainage (non-native population)	SinWod2	biv279_1	MG581711	MK574373	MK574514
S. woodiana (Lea, 1834)	Uzbekistan: Khorezm Region, Amu Darya River (41.5358°N, 60.8864°E), Aral Sea drainage (non-native population)	SinWod2	biv279_2	MG581712	MK574374	MK574515
S. woodiana (Lea, 1834)	Ukraine (non-native population)	SinWod3	isolate PB23	JQ253894	JQ253870	n/a
S. woodiana (Lea, 1834)	Ukraine (non-native population)	SinWod3	isolate PB22	JQ253893	JQ253869	n/a
S. woodiana (Lea, 1834)	China	SinWod4	isolate 117ANWE1	KJ434482	n/a	n/a
S. woodiana (Lea, 1834)	China	SinWod5	isolate 119ANWE2	KJ434483	n/a	n/a
S. woodiana (Lea, 1834)	China	SinWod4	isolate 121ANWE3	KJ434484	n/a	n/a

Taxa	Locality	Haplotype Code	Voucher Number	*COI*	*16S rRNA*	*28S rRNA*
S. woodiana (Lea, 1834)	China	SinWod4	isolate 177ANWE4	KJ434485	n/a	n/a
S. woodiana (Lea, 1834)	China	SinWod6	isolate 179ANWE5	KJ434486	n/a	n/a
S. woodiana (Lea, 1834)	Poland (non-native population)	SinWod7	n/a	AF468683	n/a	n/a
S. woodiana (Lea, 1834)	China	SinWod8	2014-phc-si-wo-001	KM272949	n/a	n/a
S. woodiana (Lea, 1834)	Poland (non-native population)	SinWod9	isolate AW19	HQ283344	GU584011	n/a
S. woodiana (Lea, 1834)	Poland (non-native population)	SinWod9	isolate AW22	HQ283345	n/a	n/a
S. woodiana (Lea, 1834)	Poland (non-native population)	SinWod9	isolate AW124	HQ283346	n/a	n/a
S. woodiana (Lea, 1834)	Poland (non-native population)	SinWod9	isolate AW123	HQ283347	n/a	n/a
S. woodiana (Lea, 1834)	Poland (non-native population)	SinWod9	isolate AW125	HQ283348	GU584010	n/a
S. woodiana (Lea, 1834)	Poland (non-native population)	SinWod10	2	EF440349	n/a	n/a
S. woodiana (Lea, 1834)	Poland (non-native population)	SinWod11	354	KJ125078	n/a	n/a
S. woodiana (Lea, 1834)	Hungary (non-native population)	SinWod9	375	KJ125079	n/a	n/a
S. woodiana (Lea, 1834)	Italy: Lake Maggiore (non-native population)	SinWod12	M21	KF731775	n/a	n/a
S. woodiana (Lea, 1834)	Italy: Po River (non-native population)	SinWod12	P15	KF731776	n/a	n/a
S. woodiana (Lea, 1834)	Italy: Po River (non-native population)	SinWod12	P32	KF731777	n/a	n/a
**Lanceolariini Froufe et al., 2017**						
***Lanceolaria* Conrad, 1853**						
Lanceolaria sp.	Vietnam	LanSp	NCSM 84945	KX822650	n/a	KX822607
L. grayii (Griffith & Pidgeon, 1833)	Russia: Far East, Primorye Region, Komissarovka River (44.8255°N, 132.0456°E), Khanka Lake basin, Okhotsk Sea drainage	LanGra1	biv502_1	MK574246	MK574384	MK574525
L grayii (Griffith & Pidgeon, 1833)	Russia: Far East, Primorye Region, Komissarovka River (44.8255°N, 132.0456°E), Khanka Lake basin, Okhotsk Sea drainage	LanGra1	biv502_2	MK574247	MK574385	MK574526
L. grayii (Griffith & Pidgeon, 1833)	Russia: Far East, Primorye Region, Komissarovka River (44.8255°N, 132.0456°E), Khanka Lake basin, Okhotsk Sea drainage	LanGra1	biv502_3	MK574248	MK574386	MK574527
L. grayii (Griffith & Pidgeon, 1833)	China: Poyang Lake, Yangtze Basin	LanGra2	NCUBM	MG933721	n/a	n/a
L. grayii (Griffith & Pidgeon, 1833)	China: Poyang Lake, Yangtze Basin	LanGra3	154LG	KJ434525	n/a	n/a
**Unionini Rafinesque, 1820**						
***Middendorffinaia* Moskvicheva & Starobogatov, 1973**						
Middendorffinaia mongolica (Middendorff, 1851)	Russia: Far East, Primorye Region, Gladkaya River (42.7065°N, 130.9084°E), Japan Sea drainage	MidMon1	biv229_2	MH974548	MK574413	MK574554
M. mongolica (Middendorff, 1851)	Russia: Far East, Primorye Region, Gladkaya River (42.7065°N, 130.9084°E), Japan Sea drainage	MidMon1	Neotype: biv229_5	MH974549	MK574414	MK574555
M. mongolica (Middendorff, 1851)	Russia: Far East, Primorye Region, Gladkaya River (42.7065°N, 130.9084°E), Japan Sea drainage	MidMon1	biv229_7	MH974550	MK574415	MK574556
M. mongolica (Middendorff, 1851)	Russia: Far East, Primorye Region, Komarovka River (43.6392°N, 132.1614°E), Razdolnaya Basin, Japan Sea drainage	MidMon2	99_3	MH974551	MK574416	MK574557
M. mongolica (Middendorff, 1851)	Russia: Far East, Primorye Region, Komarovka River (43.6392°N, 132.1614°E), Razdolnaya Basin, Japan	MidMon2	99_7	MK574277	MK574417	MK574558

Taxa	Locality	Haplotype Code	Voucher Number	*COI*	*16S rRNA*	*28S rRNA*
	Sea drainage					
M. mongolica (Middendorff, 1851)	Russia: Far East, Primorye Region, Komarovka River (43.6392°N, 132.1614°E), Razdolnaya Basin, Japan Sea drainage	MidMon3	99_8	MK574278	n/a	n/a
M. mongolica (Middendorff, 1851)	Russia: Far East, Primorye Region, Komarovka River (43.6392°N, 132.1614°E), Razdolnaya Basin, Japan Sea drainage	MidMon2	99_11	MK574279	MK574418	MK574559
M. mongolica (Middendorff, 1851)	Russia: Eastern Siberia, Transbaikalia, Shilka River (53.1048°N, 119.2200°E), Amur Basin, Okhotsk Sea drainage	MidMon4	BIV1870	MH974547	n/a	n/a
***Nodularia* Conrad, 1853**						
Nodularia douglasiae (Griffin & Pidgeon, 1833)	Russia: Far East, Amur River (49.6142°N, 128.6361°E), Okhotsk Sea drainage	NodDou1	BIV0238	MF975692	n/a	n/a
N. douglasiae (Griffin & Pidgeon, 1833)	Russia: Eastern Siberia, Transbaikalia, Onon River (49.5212°N, 112.5704°E), Amur Basin, Okhotsk Sea drainage	NodDou2	BIV0244	MF975693	MK574423	MK574564
N. douglasiae (Griffin & Pidgeon, 1833)	Russia: Eastern Siberia, Transbaikalia, Onon River (49.5212°N, 112.5704°E), Amur Basin, Okhotsk Sea drainage	NodDou3	BIV0245	MF975694	MK574424	MK574565
N. douglasiae (Griffin & Pidgeon, 1833)	Russia: Far East, Amur River (47,9754°N, 132,6801°E), Okhotsk Sea drainage	NodDou4	BIV1491	MF975695	n/a	n/a
N. douglasiae (Griffin & Pidgeon, 1833)	Russia: Far East, Amur River (47,9754°N, 132,6801°E), Okhotsk Sea drainage	NodDou5	BIV1496	MF975696	n/a	n/a
N. douglasiae (Griffin & Pidgeon, 1833)	Russia: Far East, Ussuri River, Amur Basin, Okhotsk Sea drainage	NodDou6	BIV1532	MF975697	n/a	n/a
N. douglasiae (Griffin & Pidgeon, 1833)	Russia: Far East, Ussuri River, Amur Basin, Okhotsk Sea drainage	NodDou7	BIV1533	MF975698	n/a	n/a
N. douglasiae (Griffin & Pidgeon, 1833)	China	NodDou8	HJM2	KT984764	n/a	n/a
N. douglasiae (Griffin & Pidgeon, 1833)	Japan	NodDou9	BIV0007	MF975688	n/a	n/a
N. douglasiae (Griffin & Pidgeon, 1833)	Japan	NodDou10	BIV0008	MF975689	n/a	n/a
N. douglasiae (Griffin & Pidgeon, 1833)	South Korea: Gion-Yosui	NodDou11	isolate C3	GQ451862	GQ451849	n/a
N. douglasiae (Griffin & Pidgeon, 1833)	South Korea: Gion-Yosui	NodDou12	isolate C35	GQ451863	n/a	n/a
N. douglasiae (Griffin & Pidgeon, 1833)	Russia: Far East, Khabarovsk Region, downstream of the Amur River (53.1195°N, 140.6665°E), Okhotsk Sea drainage	NodDou13	biv134_13	MK574280	MK574419	MK574560
N. douglasiae (Griffin & Pidgeon, 1833)	Russia: Far East, Primorye Region, Soldatskoye Lake (43.7747°N, 131.9406°E), Razdolnaya Basin, Japan Sea drainage	NodDou14	biv227_12	MK574281	MK574420	MK574561
N. douglasiae (Griffin & Pidgeon, 1833)	Russia: Far East, Primorye Region, Komissarovka River (44.8490°N, 132.0509°E), Khanka Lake Basin, Okhotsk Sea drainage	NodDou15	biv232_2	MK574282	n/a	n/a
N. douglasiae (Griffin & Pidgeon, 1833)	Russia: Far East, Primorye Region, Komissarovka River (44.8490°N, 132.0509°E), Khanka Lake Basin, Okhotsk Sea drainage	NodDou15	biv232_3	MK574283	n/a	n/a
N. douglasiae (Griffin & Pidgeon, 1833)	Russia: Far East, Primorye Region, Khanka Lake (44.8255°N, 132.0456°E), Okhotsk Sea drainage	NodDou13	biv503_1	MK574284	MK574421	MK574562
N. douglasiae (Griffin & Pidgeon, 1833)	Russia: Far East, Primorye Region, Khanka Lake (44.8255°N,	NodDou13	biv503_3	MK574285	MK574422	MK574563

Taxa	Locality	Haplotype Code	Voucher Nomber	*COI*	*16S rRNA*	*28S rRNA*
	132.0456°E), Okhotsk Sea drainage					
***Unio* Philipsson in Retzius, 1788**						
Unio pictorum (Linnaeus, 1758)	Ukraine: Shostka River, Dnieper Basin, Black Sea drainage	UniPic1	Biv172	KY930353	n/a	n/a
U. pictorum (Linnaeus, 1758)	Ukraine: Southern Bug, Black Sea drainage	UniPic2	Biv173	KY930354	n/a	n/a
U. pictorum (Linnaeus, 1758)	Ukraine: Snov River, Dnieper Basin, Black Sea drainage	UniPic1	Biv176	KY930355	n/a	n/a
U. pictorum (Linnaeus, 1758)	Ukraine: Southern Bug, Black Sea drainage	UniPic3	Biv177	KY930356	n/a	n/a
U. pictorum (Linnaeus, 1758)	Russia: Eastern Siberia, Transbaikalia, Kenon Lake (52.0386°N, 113.3806°E) (non-native population)	UniPic1	Biv263	KY930361	n/a	n/a
U. pictorum (Linnaeus, 1758)	Russia: Eastern Siberia, Transbaikalia, Kenon Lake (52.0386°N, 113.3806°E) (non-native population)	UniPic1	Biv269	KY930363	n/a	n/a
U. pictorum (Linnaeus, 1758)	Russia: Eastern Siberia, Transbaikalia, Kenon Lake (52.0386°N, 113.3806°E) (non-native population)	UniPic1	Biv270	KY930364	n/a	n/a
U. pictorum (Linnaeus, 1758)	Austria	UniPic4	18Up	AF156499	n/a	n/a
U. pictorum (Linnaeus, 1758)	United Kingdom	UniPic5	n/a	AF231731	n/a	n/a
U. pictorum (Linnaeus, 1758)	Poland	UniPic6	n/a	AF468684	n/a	n/a
U. pictorum (Linnaeus, 1758)	Sweden	UniPic7	NRM 42649-M064	DQ060175	DQ060163	n/a
U. pictorum (Linnaeus, 1758)	Poland	UniPic8	233	EU548056	n/a	n/a
U. pictorum (Linnaeus, 1758)	Poland	UniPic9	253	EU548057	n/a	n/a
U. pictorum (Linnaeus, 1758)	Poland	UniPic10	147	HM014130	n/a	n/a
U. pictorum (Linnaeus, 1758)	Poland	UniPic10	109	HM014131	n/a	n/a
U. pictorum (Linnaeus, 1758)	Poland	UniPic10	132	HM014132	n/a	n/a
U. pictorum (Linnaeus, 1758)	Poland	UniPic10	149	HM014133	n/a	n/a
U. pictorum (Linnaeus, 1758)	Poland	UniPic10	310	HM014134	n/a	n/a
U. pictorum (Linnaeus, 1758)	Ukraine	UniPic11	PB03	JQ253874	JQ253850	n/a
U. pictorum (Linnaeus, 1758)	Ukraine	UniPic12	PB05	JQ253876	JQ253852	n/a
U. pictorum (Linnaeus, 1758)	Russia: European part, Arkhangelsk Region, Tsiglomenka River (64.4561°N, 40.5813°E), Northern Dvina Basin, White Sea drainage	UniPic13	3HH	MK034162	MK574387	MK574528
U. pictorum (Linnaeus, 1758)	Russia: European part, Saratov Region, Khopyor River (51.5630°N, 43.1530°E), Don Basin, Azov Sea drainage	UniPic14	biv196_7	MK574249	MK574388	MK574529
U. pictorum (Linnaeus, 1758)	Russia: European part, Novgorod Region, Volkhov River (58.5197°N, 31.2776°E), Ladoga Lake basin, Baltic Sea drainage	UniPic15	biv197_1	MK574250	MK574389	MK574530
U. pictorum (Linnaeus, 1758)	Russia: European part, Novgorod Region, Volkhov River (58.5197°N, 31.2776°E), Ladoga Lake basin, Baltic Sea drainage	UniPic16	biv197_2	MK574251	MK574390	MK574531

Taxa	Locality	Haplotype Code	Voucher Number	*COI*	*16S rRNA*	*28S rRNA*
U. pictorum (Linnaeus, 1758)	Russia: European part, Saratov Region, Khopyor River (51.5630°N, 43.1530°E), Don Basin, Azov Sea drainage	UniPic17	biv282_1	MK574252	MK574391	MK574532
U. pictorum (Linnaeus, 1758)	Ukraine: Southern Bug, Black Sea drainage	UniTum1	Biv178	KY930357	n/a	n/a
U. tumidus Retzius, 1788	Ukraine: Vorskla River, Dnieper Basin, Black Sea drainage	UniTum1	Biv180	KY930358	n/a	n/a
U. tumidus Retzius, 1788	United Kingdom	UniTum2	n/a	AF231732	n/a	n/a
U. tumidus Retzius, 1788	Poland	UniTum3	n/a	AY074807	n/a	n/a
U. tumidus Retzius, 1788	Sweden: Bjornsjon Lake	UniTum4	NRM 41764-Mu7	DQ060176	DQ060161	n/a
U. tumidus Retzius, 1788	Poland	UniTum5	n/a	GU230750	n/a	n/a
U. tumidus Retzius, 1788	Ukraine	UniTum6	PB02	JQ253873	JQ253849	n/a
U. tumidus Retzius, 1788	Ukraine	UniTum7	PB06	JQ253877	JQ253853	n/a
U. tumidus Retzius, 1788	Russia: European part, Kursk Region, Seym River (51.7699°N, 36.2186°E), Dnieper Basin, Black Sea drainage	UniTum8	biv278_1	MK574253	MK574392	MK574533
U. tumidus Retzius, 1788	Russia: European part, Kursk Region, Seym River (51.7699°N, 36.2186°E), Dnieper Basin, Black Sea drainage	UniTum8	biv278_2	MK574254	MK574393	MK574534
U. tumidus Retzius, 1788	Russia: European part, Kursk Region, Seym River (51.7699°N, 36.2186°E), Dnieper Basin, Black Sea drainage	UniTum5	biv278_3	MK574255	n/a	n/a
U. tumidus Retzius, 1788	Russia: European part, Voronezh Region, Khopyor River (51.4878°N, 42.5931°E), Don Basin, Azov Sea drainage	UniTum5	biv280_7	MK574256	n/a	n/a
U. tumidus Retzius, 1788	Russia: European part, Perm Region, Iren River (57.4167°N, 56.9496°E), Volga Basin, Caspian Sea drainage	UniTum9	biv304_1	MK574257	MK574394	MK574535
U. tumidus Retzius, 1788	Russia: European part, Perm Region, Iren River (57.4167°N, 56.9496°E), Volga Basin, Caspian Sea drainage	UniTum11	biv304_6	MK574258	n/a	n/a
U. tumidus Retzius, 1788	Russia: European part, Saratov Region, Khopyor River (51.6330°N, 43.2296°E), Don Basin, Azov Sea drainage	UniTum9	biv281_1	MK574259	MK574395	MK574536
U. tumidus Retzius, 1788	Kazakhstan: Ural River (50.7131°N, 51.3808 °E), Caspian Sea drainage	UniTum11	biv594_1	MK603931	n/a	n/a
U. tumidus Retzius, 1788	Kazakhstan: Ural River (50.7131°N, 51.3808 °E), Caspian Sea drainage	UniTum11	biv594_2	MK603932	n/a	n/a
U. tumidus Retzius, 1788	Kazakhstan: Cherny Irtysh River (48.0511°N, 85.0675° E), Ob' Basin, Kara Sea drainage	UniTum11	biv623_2	MK603938	n/a	n/a
U. tumidus Retzius, 1788	Kazakhstan: Cherny Irtysh River (48.0511°N, 85.0675° E), Ob' Basin, Kara Sea drainage	UniTum11	biv623_3	MK603939	n/a	n/a
U. tumidus Retzius, 1788	Russia: European part, Arkhangelsk Region, Lacha Lake (61.3079°N, 38.8429°E), Onega Basin, White Sea drainage	UniTum12	biv568_1	MK574263	MK574399	MK574540
U. tumidus Retzius, 1788	Russia: European part, Arkhangelsk Region, Lacha Lake (61.3079°N, 38.8429°E), Onega Basin, White Sea drainage	UniTum12	biv568_2	MK574264	MK574400	MK574541
U. tumidus Retzius, 1788	Kazakhstan: Cherny Irtysh River (48.0511°N, 85.0675° E), Ob' Basin, Kara Sea drainage	UniTum13	biv623_1	MK603937	n/a	n/a
U. tumidus Retzius, 1788	Russia: Eastern Siberia, Transbaikalia, Ingoda River (52.0072°N, 113.5212°E), Amur Basin, Okhotsk Sea drainage [sequence of a juvenile mussel from gills of *Brachymystax lenok*]	UniTum14	609	MK603940	n/a	n/a

Taxa	Locality	Haplotype Code	Voucher Nomber	*COI*	*16S rRNA*	*28S rRNA*
U. crassus Retzius, 1788	Ukraine: Desna River, Dnieper Basin, Black Sea drainage	UniCra1	Biv184	KY930360	n/a	n/a
U. crassus Retzius, 1788	Sweden	UniCra2	NRM 42648-M099	DQ060174	DQ060162	n/a
U. crassus Retzius, 1788	Ukraine	UniCra3	PB09	JQ253880	JQ253856	n/a
U. crassus Retzius, 1788	Poland	UniCra4	C2	KJ525913	n/a	n/a
U. crassus Retzius, 1788	Poland	UniCra5	C4	KJ525915	n/a	n/a
U. crassus Retzius, 1788	Poland	UniCra6	C6	KJ525917	n/a	n/a
U. crassus Retzius, 1788	Lithuania	UniCra7	C7	KJ525918	n/a	n/a
U. crassus Retzius, 1788	Lithuania	UniCra8	C8	KJ525919	n/a	n/a
U. crassus Retzius, 1788	Lithuania	UniCra9	C10	KJ525921	n/a	n/a
U. crassus Retzius, 1788	Lithuania	UniCra10	C11	KJ525922	n/a	n/a
U. crassus Retzius, 1788	Czech Republic	UniCra11	ACZ1	KJ526773	n/a	n/a
U. crassus Retzius, 1788	Czech Republic	UniCra12	BCZ35	KJ526792	n/a	n/a
U. crassus Retzius, 1788	Russia: European part, Saratov Region, Khopyor River (51.5630°N, 43.1530°E), Don Basin, Azov Sea drainage	UniCra13	biv196_1	MK574265	MK574401	MK574542
U. crassus Retzius, 1788	Russia: European part, Saratov Region, Khopyor River (51.5630°N, 43.1530°E), Don Basin, Azov Sea drainage	UniCra14	biv196_2	MK574266	MK574402	MK574543
U. crassus Retzius, 1788	Russia: European part, Saratov Region, Khopyor River (51.5630°N, 43.1530°E), Don Basin, Azov Sea drainage	UniCra15	biv196_3	MK574267	MK574403	MK574544
U. crassus Retzius, 1788	Russia: European part, Voronezh Region, Khopyor River (51.4878°N, 42.5931°E), Don Basin, Azov Sea drainage	UniCra15	biv280_3	MK574268	MK574404	MK574545
U. crassus Retzius, 1788	Russia: European part, Voronezh Region, Khopyor River (51.4878°N, 42.5931°E), Don Basin, Azov Sea drainage	UniCra15	biv280_4	MK574269	MK574405	MK574546
U. crassus Retzius, 1788	Russia: European part, Voronezh Region, Khopyor River (51.4878°N, 42.5931°E), Don Basin, Azov Sea drainage	UniCra15	biv280_5	MK574270	MK574406	MK574547
U. crassus Retzius, 1788	Russia: European part, Perm Region, Iren River (57.4167°N, 56.9496°E), Volga Basin, Caspian Sea drainage	UniCra16	biv304_5	MK574271	MK574407	MK574548
U. crassus Retzius, 1788	Russia: European part, Perm Region, Iren River (57.4167°N, 56.9496°E), Volga Basin, Caspian Sea drainage	UniCra16	biv304_7	MK574272	MK574408	MK574549
U. crassus Retzius, 1788	Russia: European part, Perm Region, Iren River (57.4167°N, 56.9496°E), Volga Basin, Caspian Sea drainage	UniCra16	biv304_8	MK574273	MK574409	MK574550
U. crassus Retzius, 1788	Russia: European part, Samara Region, Samara River (53.0068°N, 50.7768°E), Volga Basin, Caspian Sea drainage	UniCra13	biv305_1	MK574274	MK574410	MK574551
U. crassus Retzius, 1788	Russia: European part, Samara Region, Samara River (53.0068°N, 50.7768°E), Volga Basin, Caspian Sea drainage	UniCra13	biv305_2	MK574275	MK574411	MK574552
U. crassus Retzius, 1788	Russia: European part, Samara Region, Samara River (53.0068°N, 50.7768°E), Volga Basin, Caspian Sea drainage	UniCra13	biv305_3	MK574276	MK574412	MK574553
U. crassus Retzius, 1788	Kazakhstan: Ural River (50.7131°N, 51.3808 °E), Caspian Sea drainage	UniCra17	biv595_1	MK603933	n/a	n/a

Taxa	Locality	Haplotype Code	Voucher Number	*COI*	*16S rRNA*	*28S rRNA*
U. crassus Retzius, 1788	Kazakhstan: Ural River (50.7131°N, 51.3808 °E), Caspian Sea drainage	UniCra17	biv595_2	MK603934	n/a	n/a
U. crassus Retzius, 1788	Kazakhstan: Ural River (50.7131°N, 51.3808 °E), Caspian Sea drainage	UniCra17	biv595_3	MK603935	n/a	n/a
U. crassus Retzius, 1788	Kazakhstan: Ural River (50.7131°N, 51.3808 °E), Caspian Sea drainage	UniCra17	biv595_4	MK603936	n/a	n/a
OUT-GROUP TAXA						
**GONIDEINAE Ortmann, 1916**						
Lamprotula leaii (Griffith & Pidgeon, 1833)	Vietnam	LamLea	biv 200/1	KY561637	KY561651	KY561669
Potomida littoralis (Cuvier, 1798)	France	PotLit	UA 21016.2	JN243905	n/a	JN243883
Pronodularia japanensis (Lea, 1859)	Japan	ProJap	NCSM 27183	KX822659	KU946322	KX822615
Gonidea angulata (Lea, 1838)	USA	GonAng	202Greeley Bar	DQ272371	n/a	AF400691
Leguminaia wheatleyi (Lea, 1862)	Turkey	LegWhe	n/a	KX822651	n/a	KX822608
Microcondylaea bonellii (A. Ferussac, 1827)	Italy	MicBon	n/a	KX822652	n/a	KX822609
**AMBLEMINAE Rafinesque, 1820**						
Amblema plicata (Say, 1817)	USA	AmbPli	n/a	U56841	n/a	AF305385
Actinonaias ligamentina (Lamarck, 1819)	USA	ActLig	UMMZ 265715	AF156517	n/a	DQ191420
Lampsilis cardium Rafinesque, 1820	USA	LamCar	n/a	AF120653	n/a	AF305386
Villosa iris (Lea, 1829)	USA	VilIri	UMMZ 265713	AF156524	n/a	DQ191422
Elliptio complanata (Lightfoot, 1786)	USA	EllCom	AUM 9711	EU448173	n/a	JF899181
Pleurobema sintoxia (Rafinesque, 1820)	USA	PleSin	UMMZ 265704	AF156509	n/a	DQ191418
Quadrula quadrula (Rafinesque, 1820)	USA	QuaQua	UMMZ 265699	AF156511	n/a	DQ191417
**MARGARITIFERIDAE Henderson, 1929**						
***Margaritifera* (Linnaeus, 1758)**						
Margaritifera dahurica (Middendorff, 1850)	Russia: Far East, Ilistaya River (43.8544°N, 132.4972°E), Khanka Lake Basin, Okhotsk Sea drainage	MargDah	biv92_6	KJ161516	KJ943526	KT343747
M. margaritifera (Linnaeus, 1758)	Russia: European part, Somba River (62.9258°N, 39.4644°E), Onega Basin, Arctic Ocean drainage	MargMar	biv618	KX550089	KX550091	KX550093
M. laevis (Haas, 1910)	Russia: Far East, Kurile Archipelago, Kunashir Island, Sennaya River (43.7489°N, 145.4658°E), Pacific Ocean drainage	MargLae	biv d0036/22	KJ161500	KJ943523	KT343742
M. middendorffi (Rosén, 1926)	Russia: Far East, Kamchatka, Nachilova River (53.0439°N, 156.7096°E), Bolshaya Basin, Okhotsk Sea drainage	MargMid	biv d0099/6	KJ161547	KJ943528	KT343745

*Chimeric sequences.

Supplementary Table 2. Evolutionary models for each partition based on Bayesian information criterion (BIC) of W-IQ-TREE

**Partition**	**Model by W-IQ-TREE**
COI	
1st codon of *COI*	F81
2nd codon of *COI*	TN+G
3rd codon of *COI*	TNe+G
16S rRNA	GTR+I+G
28S rRNA	TIM2+I

Supplementary Note. Taxonomic review of the Unionidae species in Russia

Family Unionidae Rafinesque, 1820

Subfamily Unioninae Rafinesque, 1820

Tribe Anodontini Rafinesque, 1820

Genus *Anodonta* Lamarck, 1799

=*Anodonta* Lamarck, 1799 [type species: *Mytilus cygneus* Linnaeus, 1758; monotypy[1]].

=*Colletopterum* Bourguignat, 1880 [type species: *Anodonta letourneuxi* Bourguignat, 1870 [=*Anodonta anatina*]; subsequent designation by Simpson, 1900[1]].

=*Piscinaliana* Bourguignat, 1881 [type species: *Anodonta piscinalis* Nilsson, 1823 [=*Anodonta anatina*]; subsequent designation by Locard, 1890; unavailable name, primary junior homonym of *Piscinaliana* Paladilhe, 1866, Valvatidae, Gastropoda[1]].

Anodonta anatina (Linnaeus, 1758)

=*Mytilus anatinus* Linnaeus (1758): 706[2]. Type locality: “Europae aquis dulcibus” [fresh waters of Europe].

Type: Holotype LS 258, Conchylia Linnaeana, Linnean Society of London, London, United Kingdom.

National conservation status: Least Concern (LC) (IUCN criteria) (Dataset 2).

Distribution: European Russia, Urals and Siberia up to the Lena River basin; Kazakhstan; countries of Northern, Eastern and Western Europe[3]; Selenga River basin in Mongolia[4]. A local non-native population has been recorded in Lake Imandra, Kola Peninsula [approx. 67.4683°N, 32.4707°E], in which it inhabits a channel artificially heated by the Kola Nuclear Power Plant[5].

Comments: Here, we follow the traditional concept of this variable species[3,6,7]. The correct lists of its synonyms are given by Graf[6] and Klishko et al.[7].

Anodonta cygnea (Linnaeus, 1758)

=*Mytilus cygneus* Linnaeus (1758): 706[2]. Type locality: Europe, “ad ostia fluviorum“ [Europe, mouths of rivers].

Type: Holotype LS 257, Conchylia Linnaeana, Linnean Society of London, London, United Kingdom.

National conservation status: Least Concern (LC) (IUCN criteria) (Dataset 2).

Distribution: European Russia (water bodies of the Baltic, Black, Azov, and Caspian Sea drainage basins); widespread in countries of Northern, Eastern and Western Europe[3].

Comments: Here, we follow the traditional concept of this species[3,6]. The correct list of its synonyms is given by Graf[6].

Genus *Pseudanodonta* Bourguignat, 1876

=*Pseudanodonta* Bourguignat, 1876 [type species: *Anodonta complanata* Rossmässler, 1835; subsequent designation by Westerlund, 1902[1]].

Pseudanodonta complanata (Rossmässler, 1835)

= *Anodonta complanata* Rossmässler (1835)[8]: 112. Type locality (by the lectotype): “Elbe” [Elbe River, Germany].

Type: Lectotype SMF 10652, Naturmuseum Senckenberg, Frankfurt, Germany.

National conservation status: Least Concern (LC) (IUCN criteria) (Dataset 2).

Distribution: European Russia (water bodies of the Baltic, Black, Azov, and Caspian Sea drainage basins); widespread in countries of Northern, Eastern and Western Europe[3].

Comments: Here, we follow the traditional concept of this species[3,6]. The correct list of its synonyms is given by Graf[6].

Tribe Cristariini Lopes-Lima et al., 2017

Genus *Amuranodonta* Moskvicheva, 1973

=*Amuranodonta* Moskvicheva, 1973 [type species: *Amuranodonta kijaensis* Moskvicheva, 1973; original designation[9]].

=*Amurbuldowskia* Bogatov & Starobogatov, 1996 [type species: *Buldowskia* (*Amuranodonta*) *boloniensis* Zatravkin & Bogatov, 1987; original designation[10]].

Amuranodonta kijaensis Moskvicheva, 1973

=*Amuranodonta kijaensis* Moskvicheva (1973): 832[9]. Type locality: Russia, Khabarovsk Region, Kiya River basin, near Polyotnoye Settlement, Zarechnoye Lake (47.8964°N, 135.6069°E).

=*Amuranodonta parva* Moskvicheva (1973): 832[9]. Type locality: Russia, Khabarovsk Region, Amur River near Nikolaevsk-on-Amur town (approximately 53.1333°N, 140.7416°E).

=*Amuranodonta minima* Moskvicheva (1973): 1469[11] [nomen nudum; introduced in a biogeographic account as a species from the Lower Amur below Bogorodskoye settlement (52.3690°N, 140.4201°E)].

=*Buldowskia lomakini* Zatravkin & Bogatov (1987): 86[12]. Type locality: Russia, Khabarovsk Region, Amur River near Susanino village (52.7395°N, 140.1016°E).

=*Buldowskia boloniensis* Zatravkin & Bogatov (1987): 86[12]. Type locality: Russia, Khabarovsk Region, Amur District, Bolon’ Lake (approximately 49.8614°N, 136.4653°E).

=*Amuranodonta inflata* Bogatov & Starobogatov (1996): 975[10]. Type locality: Russia, Primorye Region, a small lake in old riverbed, 0.5 km upstream of Vostretsovo settlement, right bank of the Bol’shaya Ussurka River (45.8927°N, 134.9452°E).

=*Amuranodonta pulchra* Bogatov & Starobogatov (1996): 974[10]. Type locality: Russia, Primorye Region, a small lake in old riverbed, 0.5 km upstream of Vostretsovo settlement, right bank of the Bol’shaya Ussurka River (45.8927°N, 134.9452°E).

=*Anemina ussuriensis* Qian, Fang & He (2015): 32[13]. Type locality: China, Heilongjiang Province, Hulin County, Ussuri River (approximately 45.8671°N, 133.4859°E).

Type: Holotype ZISP no. 1, Zoological Institute of the Russian Academy of Sciences, Saint Petersburg, Russia.

National conservation status: Least Concern (LC) (IUCN criteria) (Dataset 2); Status 3 – Rare species sporadically distributed over an extensive area (Red Data Book of Russia categories).

Distribution: Amur Basin in Russia and China; Lake Arey in Transbaikalia; putative endemic lineage to the Amur Basin. This species has a rather fragmentary distribution due to the preference to specific habitats, i.e. floodplain lakes and pool sites on large rivers.

Comments: The single *Amuranodonta* species having elongated, narrow shell is known from the Amur Basin. Moskvicheva[9] introduced two nominal *Anemina*-like taxa with an elongated shell: *Amuranodonta kijaensis* and *A. parva*. Acting as the First Revisers, we choice *Amuranodonta kijaensis* as the valid name for this species. Six additional nominal taxa having the elongated shell were described from the Amur Basin[9,10,12,13]. These taxa are considered junior synonyms of *Amuranodonta kijaensis*.

Genus *Beringiana* Starobogatov in Zatravkin, 1983

= *Beringiana* Starobogatov in Zatravkin, 1983 [type species: *Anodonta cellensis* var. *beringiana* Middendorff, 1851; original designation[14]].

=*Kunashiria* Starobogatov in Zatravkin, 1983 [type species: *Anodonta japonica* Clessin, 1874; original designation[14]].

=*Arsenievinaia* Zatravkin & Bogatov, 1987 [type species: *Amuranodonta sihotealinica* Zatravkin & Starobogatov, 1984; original designation[12]].

Beringiana beringiana (Middendorff, 1851)

=*Anodonta cellensis* var. *beringiana* Middendorff (1851): 284[15]. Type locality: USA, Aleutian Islands, Unalashka Island, Kenai Lake.

=*Anodonta youkanensis* Lea (1867): 81[16]. Type locality: Head waters of the Yukon, Arctic America.

=*Anodonta beringiana* var. *taranetzi* Zhadin (1938): 133[17]. Type locality: Russia, Sakhalin Island, a lake in the floodplain of Tym' River near Kotik Island.

=*Anodonta iwakawai* Suzuki (1939): 130[18]. Type locality: Japan, Tarukawa-numa, NW of Sapporo.

=*Amuranodonta sihotealinica* Zatravkin & Starobogatov (1984): 1788[19]. Type locality: Russia, Primorye Region, a tributary of Kievka River at 10-12 km from its mouth.

=*Arsenievinaia coptzevi* Zatravkin & Bogatov (1987): 104[12]. Type locality: Russia, Primorye Region, Vas'kovskoye Lake near village Rudnaya Pristan' (44.3449°N, 135.8162°E).

=*Arsenievinaia zimini* Zatravkin & Bogatov (1987): 102[12]. Type locality: Russia, Primorye Region, Zarya Lake, 30 km SW from Valentin village (43.0335°N, 134.1506°E).

=*Arsenievinaia zarjaensis* Bogatov & Zatravkin (1988): 166[20]. Type locality: Russia, Primorye Region, Zarya Lake (43.0335°N, 134.1506°E).

=*Arsenievinaia alimovi* Bogatov & Zatravkin (1988): 165[20]. Type locality: Russia, Primorye Region, Avakumovka River (approx. 43.7578°N, 135.1865°E).

=*Arsenievinaia compressa* Bogatov & Starobogatov (1996): 1332[21]. Type locality: Russia, Primorye Region, Dal'negorsk District, Rudnaya River basin, Lake Zerkalnoye [it is most likely Lake Zerkalnoye in Zerkalnaya River basin, Kavalerovsky District (44.1677°N, 135.6049°E)].

=*Arsenievinaia zatravkini* Bogatov & Starobogatov (1996): 1331[21]. Type locality: Russia, Primorye Region, Dal'negorsk District, Rudnaya River basin, Lake Zerkalnoye [it is most likely Lake Zerkalnoye in Zerkalnaya River basin, Kavalerovsky District (44.1677°N, 135.6049°E)].

=*Beringiana compressa* Sayenko & Bogatov (1998): 1414[22] [unvailable name: a primary junior homonym of *Arsenievinaia compressa* Bogatov & Starobogatov, 1996]. Type locality: Russia, North Kurile Islands, Paramushir Island, Pernatoye Lake (50.0425°N, 155.3956°E).

=*Kunashiria japonica boreosakhalinensis* Labay & Shulga (1999): 77[23]. Type locality: Russia, northwestern Sakhalin, Sladkoye Lake (53.3849°N, 141.9907°E).

=*Anemina* (*Buldowskia*) *lacustris* Labay & Shulga (1999): 78[23]. Type locality: Russia, northwestern Sakhalin, Sladkoye Lake (53.3849°N, 141.9907°E).

=*Anemina* (*Buldowskia*) *adotymensys* Labay & Shulga (1999): 79[23]. Type locality: Russia, central Sakhalin, Tym' River basin, Krivushka Lake near Ado-Tymovo village (51.1375°N, 142.6510°E).

=*Kunashiria iturupica* Bogatov, Sayenko & Starobogatov (1999): 60[24]. Type locality: Russia, South Kurile Islands, Iturup Island, Kuibyshevskoye Lake (45.0744°N, 147.6581°E).

=*Kunashiria sinanodontoides* Bogatov, Sayenko & Starobogatov (1999): 59[24]. Type locality: Russia, South Kurile Islands, Iturup Island, Dobroye Lake (44.7472°N, 147.2336°E).

=*Beringiana kamchatica* Bogatov & Starobogatov (2001): 27[25]. Type locality: Russia, Kamchatka Peninsula, vicinities of Petropavlovsk-Kamchatsky city, Blizhnyaya River, source of Blizhneye Lake (52.9713°N, 158.3268°E).

=*Beringiana derzhavini* Bogatov & Starobogatov (2001): 27[25]. Type locality: Russia, Russia, Kamchatka Peninsula, vicinities of Petropavlovsk-Kamchatsky city, Blizhnyaya River, source of Blizhneye Lake (52.9713°N, 158.3268°E).

=*Beringiana chereshnevi* Bogatov & Starobogatov (2001): 26[25]. Type locality: Russia, Chukotka, Elergytgyn Lake [68.8059°N, 171.2842°E], Khatarka River basin]. Vinarski and Kantor[1] suggested that the Elgygytgyn Lake (67.5°N, 172.1°E) can be the true type locality, but this assumption seems to be incorrect. The type series was collected by Igor A. Chereshnev, an ichthyologist from the Institute of Biological Problems of the North, Far Eastern Branch of the Russian Academy of Sciences. The Elergytgyn Lake is a small lake belonging to the Rytkuchy River basin near the Chaun Research Station of this scientific institute. We were unable to find the location of the Khatarka River on the map, but it may be a local name of the Rytkuchy River.

Types: Holotype ZISP no. 1, Zoological Institute of the Russian Academy of Sciences, Saint Petersburg, Russia.

National conservation status: Least Concern (LC) (IUCN criteria) (Dataset 2).

Distribution: Widespread in the rivers east of the Lena Basin (Kolyma Highlands, Chukotka, and Kamchatka), coastal rivers of the Japan and Okhotsk Sea drainage basins northeast of Vladivostok (from the Kievka River), Kuriles and Sakhalin; Hokkaido[18]; Alaska, Western Pacific Region, and Canada in North America[26]. Not known from the Amur and Razdolnaya basins and from coastal rivers southwest of Vladivostok and west of Nakhodka.

Comments: This variable and widespread species has been described under various names, the oldest available of which is *Beringiana beringiana*. The majority of younger available names were once applied to morphological varieties with minute differences in the shell shape and convexity that were thought to be endemics of certain lakes, streams, and rivers[12,19-25]. We place these names to the synonymy of *Beringiana beringiana* based on conchological similarity, multi-locus molecular data, and geographic evidence. In contrast, *Beringiana georgiensis* Bogatov & Starobogatov, 2001 (type locality: Fort George, Oregon, USA[25]) belongs to another genus and is a junior synonym of *Anodonta oregonensis* Lea, 1838 (based on molecular sequences of the topotypes from the Columbia River basin; our unpublished data).

Genus *Buldowskia* Moskvicheva, 1973

=*Buldowskia* Moskvicheva, 1973 [type species: *Anodonta arcaeformis* var. *suifunica* Lindholm, 1925; original designation[9]].

Buldowskia suifunica (Lindholm, 1925)

=*Anodonta arcaeformis* var. *suifunica* Lindholm (1925): 138[27]. Type locality: "der Umgebung von Nikolsk-Ussuriisk aus einer Bucht des Flusses Suifun" [Russia, Primorye Region, Razdolnaya (=Sujfun) River near Ussuriysk city (approximately 43.7623°N, 131.9600°E)].

=*Anodonta beringiana* var. *sujfunensis* Zhadin (1938): 132[17]. Type locality: Russia, Primorye Region, Razdolnaya (=Sujfun) Basin, Bokhayskoye Lake.

=*Anodonta woodiana* var. *elliptica* Zhadin (1938): 144[17] [identification error: non Heude, 1878].

=*Anodonta arcaeformis* Zhadin (1938): 148[17] [identification error: non Heude, 1877].

=*Anodonta euscaphys* Zhadin (1938): 149[17] [partim (samples from Razdolnaya Basin), identification error: non Heude, 1879]

=*Buldowskia suputinensis* Moskvicheva (1973): 833[9]. Type locality: Russia, Primorye Region, vicinities of Ussuriysk city, Komarovka Basin, Lug Lake.

=*Amuranodonta starobogatovi* Moskvicheva (1973): 831[9]. Type locality: Russia, Primorye Region, vicinities of Ussuriysk city, a small lake on the Krasnoyarskaya mound (approximately 43.7778°N, 132.0100°E).

=*Buldowskia cylindrica* Moskvicheva (1973): 1467[11]. Type locality: Russia, Primorye Region, Batalyanza (currently Knevichanka) River.

=*Anemina* (*Buldowskia*) *zatrawkini* Martynov & Chernyshev (1992): 21[28]. Type locality: Russia, Primorye Region, Razdolnaya Basin, an irrigation channel near Kiparisovo station (43.4710°N, 131.9170°E).

=*Anemina* (*Anemina*) *shadini deflexa* Martynov & Chernyshev (1992): 19[28]. Type locality: Russia, Primorye Region, vicinities of Vladivostok city, an unnamed lake near the Basargin Cape (43.0762°N, 131.9612°E).

=*Buldowskia possietica* Bogatov & Starobogatov (1996): 1329[21]. Type locality: Russia, Primorye Region, Khasan District, an oxbow of the Gladkaya River, 2 km from Gvozdevo settlement (42.7065°N, 130.9084°E).

=*Buldowskia koreana* Bogatov & Starobogatov (1996): 1331[21]. Type locality: Russia, Primorye Region, Khasan District, lake of former bed of the Gladkaya River in 2 km of Gvozdevo settlement (42.7065°N, 130.9084°E).

Type: Holotype ZISP no. 1, Zoological Institute of the Russian Academy of Sciences, Saint Petersburg, Russia.

National conservation status: Vulnerable (VU) B1ab(i,ii,iii,iv) (IUCN criteria) (Dataset 2); Status 3 – Rare species inhabiting a limited area (Red Data Book of Russia categories).

Distribution: Razdolnaya Basin, coastal rivers southwest of Vladivostok; should also be found in northeastern China (at least within Razdolnaya Basin) and North Korea.

Comments: One *Buldowskia* species was recorded from the Razdolnaya Basin and coastal rivers southwest of Vladivostok. *Buldowskia suifunica* seems to be the oldest available name for this species. The other nominal taxa described from these freshwater basins[9,11,17,21,28] are considered synonyms of this species based on the similar shell shape, prononced umbo, multi-locus molecular data, and geographic evidence. Bogatov and Prozorova[29] erroneously listed this species as *Buldowskia flavotincta* (Martens, 1905), but the latter name corresponds to another *Buldowskia* species endemic to South Korea[30] that was not recorded in Russia (our unpublished data).

Buldowskia shadini (Moskvicheva, 1973)

=*Anodonta euscaphys* Zhadin (1938): 149[17] [partim (samples from Lake Khanka and Ussuri Basin), identification error: non Heude, 1879]

=*Sinanodonta* (*Anemina*) *shadini* Moskvicheva (1973): 828[9]. Type locality: Russia, Primorye Region, Khanka Lake basin, Mandzhurka (Novo-Troitskaya) River.

=*Sinanodonta* (*Anemina*) *fuscoviridis* Moskvicheva (1973): 830[9]. Type locality: Russia, Primorye Region, Ulakhe River (a tributary of the Ussuri River) near Koksharovka village (44.5253°N, 134.0544°E).

=*Sinanodonta* (*Anemina*) *buldowskii* Moskvicheva (1973): 829[9]. Type locality: Russia, Primorye Region, Ulakhe River (a tributary of the Ussuri River), 150 m from the northern shore of Lake Khanka.

=*Buldowskia* (*Amurbuldowskia*) *sitaensis* Bogatov & Starobogatov (1996): 973[10]. Type locality: Russia, Khabarovsk Region, inlet of the Sita River near bridge of road Khabarovsk–Vladivostok in vicinity of Knyaze-Volkonskoye settlement (48.4661°N, 135.4478°E).

Type: Holotype ZISP no. 1, Zoological Institute of the Russian Academy of Sciences, Saint Petersburg, Russia.

National conservation status: Least Concern (LC) (IUCN criteria) (Dataset 2).

Distribution: Amur Basin in Russia; Lake Buir (Amur Basin) in Mongolia and northeastern China [ANSP 416357, Academy of Natural Sciences, Philadelphia, USA[31]]; South Korea[30].

Comments: One *Buldowskia* species with ovate shell is known to occur in the Amur Basin. Moskvicheva[9] introduced this species under three different names. Acting as the First Revisers, we choice *Buldowskia shadini* as the valid name for this species. Other nominal *Anemina*-like taxa with ovate shell described from the Amur Basin[9,10] are considered synonyms of this species.

Genus *Cristaria* Schumacher, 1817

= *Cristaria* Schumacher, 1817 [type species: *Cristaria tuberculata* Schumacher, 1817; monotypy[1]].

Cristaria plicata (Leach, 1814)

=*Dipsas plicatus* Leach (1814): 120. Type locality: “A Bohemian river”[32] [erroneous; it is most likely China].

Types: Not traced.

National conservation status: Least Concern (LC) (IUCN criteria) (Dataset 2).

Distribution: Amur Basin in Russia, Mongolia and northeastern China; one record from Tym’ River, central Sakhalin; Korea, Japan, eastern China (Yangtze Basin), and northern Vietnam[33-37]. Records from the Mekong Basin[38,39] may represent a historical human-mediated or natural dispersal event.

Comments: Here, we follow the commonly accepted concept of this species[6,35,36]. The correct list of synonyms is presented by Klishko et al.[35,36].

Genus *Sinanodonta* Modell, 1945

=*Sinanodonta* Modell, 1945 [type species: *Symphynota magnifica* Lea, 1834; typification of a replaced name[1]].

=*Cristariopsis* Moskvicheva, 1973 [type species: *Sinanodonta* (*Cristariopsis*) *crassitesta* Moskvicheva, 1973; original designation[9]].

=*Ellipsanodon* Bogatov & Starobogatov, 1996 [type species: *Sinanodonta* (*Ellipsanodon*) *ovata* Bogatov & Starobogatov, 1996; original designation[21]].

Sinanodonta lauta (Martens, 1877)

=*Anodonta lauta* Martens (1877): 117. Type locality: “Im heiligen See bei Uweno, bei Yeddo”[40] [Japan, Tokyo, Ueno Park, Shinobazu Pond (35.7130°N, 139.7708°E)].

=*Sinanodonta woodiana fukudai* Modell (1945): 96. Type locality: “Zenra Dô, Fuan Gun, Turuppo, Nordkorea”[41] [North Korea].

=*Sinanodonta ovata* Bogatov & Starobogatov (1996): 1328[21]. Type locality: Russia, Primorye Region, Khasan District, channel of Gladkaya River 8 km upstream of the mouth (42.7065°N, 130.9084°E).

=*Sinanodonta manchurica* Bogatov & Starobogatov (1996): 1329[21]. Type locality: Russia, Primorye Region, a lake of former bed of Gladkaya River in 2 km of Gvozdevo settlement (42.7065°N, 130.9084°E).

Types: Syntypes ZMB 28145, Museum für Naturkunde, Berlin, Germany.

National conservation status: Endangered (EN) B1ab(i,ii,iii,iv) (IUCN criteria) (Dataset 2); Status 3 – Rare species inhabiting a limited area (Red Data Book of Russia categories).

Distribution: Native to coastal rivers southwest of Vladivostok; Japan and Korea[30]. A non-native population in the Yenisei River, Eastern Siberia[42].

Comments: This interesting species has a broad range, but in Russia its native populations are known from a few coastal freshwater basins close to the boundary of North Korea. Bespalaya et al.[42] and Kondakov et al.[43] mentioned this species as *Sinanodonta ovata* based on the results of molecular analyses of the topotypes of this nominal taxon from the Gladkaya River. Kondakov et al.[43] assumed that this species may have an older available name among the Japanese nominal taxa. *Sinanodonta lauta* seems to be the oldest name for this species. Two nominal taxa described from the Gladkaya River are considered synonyms of *Sinanodonta lauta*.

Sinanodonta schrenkii (Lea, 1870)

=*Anodonta magnifica* Schrenck (1867): 718[44] [identification error].

=*Margaron (Anodonta) schrenkii* Lea (1870): 75[45] [new name for *Anodonta magnifica* Schrenck]. Type locality: Amur River.

=*Anodonta woodiana* Zhadin (1938): 140[17] [identification error: non Lea, 1834].

=*Sinanodonta schrencki* Moskvicheva (1973): 826[9] [new name for *Anodonta magnifica* Schrenck]. Type locality: Russia, Primorye Region, Ussuri River near the Khor River mouth (47.8139°N, 134.6859°E).

=*Sinanodonta amurensis* Moskvicheva (1973): 826[9]. Type locality: Russia, Khabarovsk Region, vicinity of Khabarovsk city, Malyie Chepchiki channel (48.72°N, 135.50°E).

=*Sinanodonta crassitesta* Moskvicheva (1973): 828[9]. Type locality: Russia, Primorye Region, Razdolnaya (=Sujfun) River basin, downstream of Komarovka (Suputinka) River (approximately 43.7645°N, 131.9625°E).

=*Sinanodonta likharevi* Moskvicheva (1973): 826[9]. Type locality: Russia, Transbaikalia, Shilka River near Sretensk-Zabaikalskiy town (52.2°N, 117.7°E).

=*Sinanodonta* (s. str.) *renzini* Bogatov & Zatravkin (1988): 160[20]. Type locality: Russia, Primorye Region, Razdolnaya River basin, a channel near Kiparisovo station (43.4710°N, 131.9170°E).

=*Sinanodonta* (*Cristariopsis*) *primorjensis* Bogatov & Zatravkin (1988): 162[20]. Type locality: Russia, Primorye Region, Razdolnaya River basin, Lake Soldatskoye (43.7750°N, 131.9408°E).

Type: Holotype (by monotypy) illustrated in Figs. 1-2, Tab. 28 in Schrenck[44,45]. Lea (p. 75)[45] introduced this taxon as follows: “The figure given by Dr. Schrenck in Reisen und Forschungen im Amur-Lande is not my *magnifica*. I therefore propose to call it *Schrenkii*”.

National conservation status: Least Concern (LC) (IUCN criteria) (Dataset 2).

Distribution: Amur and Razdolnaya basins in Russia; Halhin River in Mongolia (a tributary of Lake Buir, Amur Basin) [ANSP 416366, Academy of Natural Sciences, Philadelphia, USA[31]], and South Korea[30].

Comments: One *Sinanodonta* species inhabits the Amur and Razdolnaya basins. Sayenko et al.[46] applied the name *Sinanodonta amurensis* Moskvicheva, 1973 to this species. However, Kondakov et al.[43] showed that *Sinanodonta schrenkii* (Lea, 1870) is the valid oldest name for this taxon. Fisher[47] listed Lea’s name for this species in his “Catalogue et distribution geographique des mollusques terrestres, fluviatiles & marins”. The rest of nominal taxa of *Sinanodonta* described from the Amur and Razdolnaya basins are considered synonyms of this species based on conchological similarity, multi-locus molecular data, and geographic evidence.

Sinanodonta woodiana (Lea, 1834)

=*Symphynota woodiana* Lea (1834): 42 [154][48]. Type locality: China.

Type: Syntype USNM 86380, National Museum of Natural History, Washington, USA.

National conservation status: Non-native species, Not Applicable (NA).

Distribution: A non-native population of this species has been discovered in the Yenisei River, Eastern Siberia[42]. Native to the Yangtze River basin in China but widely introduced in Europe, Central Asia, and Myanmar[42,43,49-51].

Comments: *Sinanodonta woodiana* in its current understanding represents a complex of 6-7 cryptic species[33,49,52]. The taxonomic position of the temperate invasive lineage is unclear. While Kondakov et al.[43] assumed that *Sinanodonta gibba* can be its oldest available name, Dr. Arthur Bogan (pers. comm., 2019) recommend to use *S. woodiana*. This latter concept seems to be correct, and we follow this point of view in the present study.

Tribe Lanceolariini Froufe et al., 2017

Genus *Lanceolaria* Conrad, 1853

=*Lanceolaria* Conrad, 1853 [type species: *Unio grayanus* Lea, 1834; monotypy[1]].

=*Cylindrica* Simpson, 1900 [type species: *Nodularia cylindrica* Simpson, 1900; original designation[53]; unavailable name, primary homonym of *Cylindrica* Clessin, 1882 (Gastropoda: Hydrobiidae)[54]].

=*Pericylindrica* Tomlin, 1930 [new name for *Cylindrica* Simpson, 1900[54]].

=*Prolancealaria* Moskvicheva, 1973 [type species: *Unio grayii* Griffith & Pidgeon, 1833; original designation[11]].

Lanceolaria grayii (Griffith & Pidgeon, 1833)

=*Unio grayii* Griffith & Pidgeon (1833): Pl. 21[55]. Type locality: Not indicated.

=*Unio grayanus* Schrenck (1867): 694[44] [identification error: non Lea, 1834].

=*Unio grayanus* var. *decurvata* Schrenck (1867): 694[44] [identification error: non Rossmässler, 1835].

=*Nodularia cylindracea* Simpson (1900): 84[56] [new name for *Unio grayanus* Schrenck; incorrect original spelling of *Nodularia cylindrica* Simpson]. Type locality: China [incorrect, it is Russia, Primorye Region, Ussuri River[44]].

=*Nodularia cylindrica* Simpson (1900): 807[53] [correct name for *Nodularia cylindracea* Simpson]. Type locality: Amur land [correct type locality for this taxon].

=*Lanceolaria grayana* Zhadin (1938): 103[17] [identification error: non Lea, 1834].

=*Lanceolaria ussuriensis* Moskvicheva (1973): 1465[11] [new name for *Unio grayanus* var. *decurvata* Schrenck]. Type locality: Russia, Primorye Region, Khanka Lake near Troitzkoye village (44.8179°N, 132.0410°E).

=*Lanceolaria chankensis* Moskvicheva (1973): 1466[11]. Type locality: Russia, Primorye Region, Khanka Lake basin, Mel'gunovka River (44.5768°N, 132.0754°E).

=*Lanceolaria maacki* Moskvicheva (1973): 1465[11]. Type locality: Russia, Primorye Region, Ussuri River.

=*Lanceolaria (Pericylindrica) bogatovi* Zatravkin & Starobogatov (1984): 1785[19]. Type locality: Russia, Khabarovsk Region, Amur River, Sakhalyan Island against Amursk city, an inner lake connected with the Amur River (50.2043°N, 136.8978°E).

Type: Not traced but pictured by Griffith and Pidgeon (1833): Pl. 21, Fig. 3[55].

National conservation status: Least Concern (LC) (IUCN criteria) (Dataset 2); Status 3 – Rare species sporadically distributed over an extensive area (Red Data Book of Russia categories).

Distribution: Lake Khanka, Ussuri Basin and Lower Amur River in Russia; Yangtze Basin in China[37].

Comments: The only *Lanceolaria* species occurring in the Khanka Lake, Ussuri, and Lower Amur basins. While several species-group names were introduced for this Russian lineage[11,19,44,53], it appeared to represent a local population of the widespread *Lanceolaria grayii*. Five nominal taxa described from Russia are considered synonyms of this species.

Tribe Unionini Rafinesque, 1820

Genus *Middendorffinaia* Moskvicheva & Starobogatov, 1973

=*Middendorffinaia* Moskvicheva & Starobogatov, 1973 [type species: *Unio mongolicus* Middendorff, 1851; original designation[57]].

=*Suifununio* Moskvicheva & Starobogatov, 1973 [type species: *Middendorffinaia* (*Suifununio*) *suifunensis* Moskvicheva & Starobogatov, 1973; original designation[57]].

=*Pseudopotomida* Moskvicheva & Starobogatov, 1973 [type species: *Middendorffinaia* (*Pseudopotomida*) *shadini* Moskvicheva & Starobogatov, 1973; original designation[57]].

Middendorffinaia mongolica (Middendorff, 1851)

=*Unio mongolicus* Middendorff (1851): 277[15]. Type locality (determined by the neotype): Russia, Primorye Region, downstream of Gladkaya River (42.7065°N, 130.9084°E) [former type locality based on the original description: Russia, Transbaikalia, Shilka River basin, a mountain spring near Gorbitsa village (53.1027°N, 119.2169°E)].

=*Unio continentalis* Zhadin (1938): 100[17] [identification error: non Haas, 1910].

=*Middendorffinaia* (*Suifununio*) *suifunensis* Moskvicheva & Starobogatov (1973): 30[57]. Type locality: Russia, Primorye Region, Razdolnaya River, 1.5 km south of Razdolnaya station (43.5467°N, 131.8920°E).

=*Middendorffinaia (Pseudopotomida) shadini* Moskvicheva & Starobogatov (1973): 31[57]. Type locality: Russia, Primorye Region, Razdolnaya River basin, Rakovka River (approximately 43.8459°N, 131.9591°E).

=*Middendorffinaia (Pseudopotomida) dulkeitiana* Moskvicheva & Starobogatov (1973): 33[57]. Type locality: Russia, Primorye Region, a tributary of Komarovka (=Suputinka) River near Ussuriysk city (approximately 43.7838°N, 131.9620°E).

=*Middendorffinaia (Pseudopotomida) weliczkowskii* Moskvicheva & Starobogatov (1973): 33[57]. Type locality: Russia, Primorye Region, Razdolnaya River basin, Olenevka River (43.7493°N, 131.9673°E).

=*Middendorffinaia (Pseudopotomida) hassanica* Moskvicheva & Starobogatov (1973): 34[57]. Type locality: Russia, Primorye Region, Lake Khasan (42.4386°N, 130.6227°E).

=*Middendorffinaia (Pseudopotomida) martensi* Moskvicheva & Starobogatov (1973): 34[57]. Type locality: Russia, Primorye Region, Tesnaya River between Kraskino and Khasan stations (approximately 42.7288°N, 130.6642°E).

=*Middendorffinaia maihensis* Moskvicheva (1973): 1466[11]. Type locality: Russia, Primorye Region, Artemovka River near Artemovka village [a village bearing such a name does not exist; it may actually be the Shtykovo village in the downstream of Artemovka River: 43.3840°N, 132.3778°E].

=*Middendorffinaia alimovi* Bogatov (2012): 400[58]. Type locality: Russia, Amur Region, Amur River drainage, Upper Zeya Basin, Argi River opposite the Orlinoye Lake (54.4424°N, 129.4831°E).

Type: Neotype RMBH biv229_5 (designated in this study, see Taxonomic Account and and Fig. 5A), Russian Museum of Biodiversity Hotspots, Federal Center for Integrated Arctic Research of the Russian Academy of Sciences, Arkhangelsk, Russia.

National conservation status: Least Concern (LC) (IUCN criteria) (Dataset 2); Status 3 – Rare species sporadically distributed over an extensive area (Red Data Book of Russia categories).

Distribution: Amur and Razdolnaya basins, coastal rivers of the Japan Sea drainage west of Nakhodka (Partizanskaya and Artemovka rivers[59]) and southwest of Vladivostok; Onon and Argun basins in Mongolia[4]; may also be found in the Chinese part of the Amur Basin and in North Korea; putative endemic species to the region having a rather fragmentary distribution due to the preference to specific habitats, i.e. fast flowing rivers with gravel-sandy and sandy bottom. The record from the Kuchtuy River Basin (Okhotsk Region)[60,61] belongs to *Nodularia douglasiae*.

Comments: In this study, we provide an updated concept of this variable species (see Taxonomic Account). Klishko et al.[61] listed *Middendorffinaia ochotica*, *M. arsenjevi* and *M. ussuriensis* as synonyms of *M. mongolica*, but the holotypes of the latter taxa share almost straight hinge plate and undoubtedly belong to *Nodularia douglasiae* (see below).

Genus *Nodularia* Conrad, 1853

=*Nodularia* Conrad, 1853 [type species: *Unio douglasiae* Griffith & Pidgeon, 1833; monotypy[1]].

=*Amurunio* Zatravkin & Bogatov, 1987 [type species: *Nodularia lebedevi* Zatravkin & Starobogatov, 1984; original designation[12]].

=*Magadaninaia* Martynov & Chernyshev, 1992 [type species: *Nodularia* (*Magadaninaia*) *extremalis* Martynov & Chernyshev, 1992; original designation[28]].

Nodularia douglasiae (Griffith & Pidgeon, 1833)

=*Unio douglasiae* Griffith & Pidgeon (1833): Pl. 21[55]. Type locality: Not indicated.

=*Unio pictorum* var. *amurensis* Mousson (1887): 26[62]. Type locality: “Chabarofka, Nicolajewsk” [Russia, Amur River near Khabarovsk city and Nikolaevsk-on-Amur town].

=*Unio middendorffi* Westerlund (1890): 113[63]. Type locality: Nercha River near Nerchinsk (51.9843°N, 116.5425°E).

=*Unio abbreviatus* Westerlund (1897): 132[64]. Type locality: Russia, Amur River near Nikolaevsk-on-Amur town (approximately 53.1341°N, 140.7352°E)].

=*Unio schrencki* Westerlund (1897): 131[64]. Type locality: “Amur-Fluss” [Russia, Amur River].

=*Nodularia douglasiae flavoviridis* Haas (1910): 71[65]. Type locality: “W. Ufer d. Sees Buir-nor, O. Mongolei, Amur-Geb.” [Mongolia, Amur Basin, Buir Lake, western shore (47.7618°N, 117.5094°E)].

=*Nodularia sujfunica* Moskvicheva (1973): 1463[11]. Type locality: Suifun River near Razdolnaya station [Russia, Primorye Region, Razdolnaya River near Razdolnaya station (43.5558°N, 131.9065°E)].

=*Nodularia vladivostokensis* Moskvicheva (1973): 1463[11]. Type locality: Suifun River near Baranovsky station [Russia, Primorye Region, Razdolnaya River near Baranovsky station (43.6579°N, 131.9222°E)].

=*Middendorffinaia ussuriensis* Moskvicheva & Starobogatov (1973): 28[57]. Type locality: Russia, Primorye Region, Arsenievka (=Daubikhe) River near Yakovlevka village (44.4209°N, 133.5028°E).

=*Middendorffinaia arsenjevi* Moskvicheva & Starobogatov (1973): 29[57]. Type locality: Russia, Primorye Region, Ussuri Basin, Pashino River near Dormidontovka village (47.7641°N, 134.9071°E).

=*Nodularia lebedevi* Zatravkin & Starobogatov (1984): 1787[19]. Type locality: Russia, Khabarovsk Region, Amur River, Sakhalyan Island against Amursk city, an inner lake connected with the Amur River (50.2043°N, 136.8978°E).

=*Nodularia* (*Magadaninaia*) *extremalis* Martynov & Chernyshev (1992): 19[28]. Type locality: Russia, Magadan Region, Ol'sky District, in a river near Motykleyka settlement (59.4159°N, 148.7101°E).

=*Nodularia* (*Nodularia*) *moskvichevae* Bogatov & Starobogatov (1992): 134[66]. Type locality: Russia, Primorye Region, 1 km from Razdolnaya station, backwater of Razdolnaya River (43.5558°N, 131.9065°E).

=*Middendorffinaia ochotica* Bogatov (2000): 861[60]. Type locality: Russia, Khabarovsk Region, Kukhtui River north of Okhotsk (approximately 59.4319°N, 143.2515°E).

=*Nodularia sakhalinensis* Bogatov (2001): 75[67]. Type locality: Russia, northern Sakhalin, Langry River near North-Sakhalinsk-I neolithic site (approximately 52.9574°N, 142.2284°E).

Types: Not traced.

National conservation status: Least Concern (LC) (IUCN criteria) (Dataset 2).

Distribution: Amur and Razdolnaya basins[68] and coastal rivers of the Okhotsk Sea drainage basin[28,60,68]; one record from Langry River, northwestern Sakhalin[67]; Lake Buir (Amur Basin) in Mongolia and northeastern China[65], eastern China (Yangtze Basin), South Korea, Japan, and northern Vietnam[37,68,69].

Comments: In this study, we provide an updated concept of this variable species (see Taxonomic Account). *Middendorffinaia ochotica*, *M. arsenjevi* and *M. ussuriensis* were considered new synonyms of *Nodularia douglasiae*.

Genus *Unio* Retzius, 1788

=*Unio* Retzius, 1788 [type species: *Mya pictorum* Linnaeus, 1758 (subsequent designation by Turton, 1831[1]]

=*Tumidiana* Servain, 1882 [type species: *Unio tumidus* Retzius, 1788; subsequent designation by Kantor & Sysoev, 2005[1]]

=*Crassiana* Servain, 1882 [type species: *Unio crassus* Retzius, 1788; subsequent designation by Graf, 2010[1]]

Unio pictorum (Linnaeus, 1758)

=*Mya pictorum* Linnaeus (1758): 671[2]. Type locality: “Europae fluviis” [European rivers].

Types: Syntypes LS 28, Conchylia Linnaeana, Linnean Society of London, London, United Kingdom.

National conservation status: Least Concern (LC) (IUCN criteria) (Dataset 2).

Distribution: European Russia, western Urals, countries of Northern, Eastern and Western Europe[3]; introduced in Lake Kenon, Amur Basin, Transbaikalia[70]. A BLASTn search reveals that the non-native population in Lake Kenon shares a widespread COI haplotype[70] that was recorded from the Dnieper Basin in Ukraine, Danube River in Slovakia, Seine and Somme rivers in France, Greece, and Iran[71,72].

Comments: Here, we follow the traditional concept of this species[6,70,71]. The correct lists of synonyms are given by Graf[6] and Klishko et al.[70].

Unio tumidus Retzius, 1788

=*Unio tumidus* Retzius (1788): 17[73]. Type locality: “fluviis Europae” [European rivers].

Types: Not traced.

National conservation status: Least Concern (LC) (IUCN criteria) (Dataset 2).

Distribution: European Russia, western Urals; Ural River in Russia and Kazakhstan; Irtysh Basin in Western Siberia[74] and Kazakhstan; historical records of recent shells from the southern part of the

Ob’ River basin in Western Siberia[17]; countries of Northern, Eastern and Western Europe[3]; introduced in Lake Kenon[70] and the Upper Amur Basin in Transbaikalia. A BLASTn search reveals that the non-native population in the Upper Amur Basin shares a widespread COI haplotype that was recorded from the Dnieper Basin in Ukraine, Danube River in Slovakia, and Poland[70,71].

Comments: Here, we follow the traditional concept of this species[6,70,71]. The correct lists of synonyms are given by Graf[6] and Klishko et al.[70].

Unio crassus Retzius, 1788

=*Unio crassus* Retzius (1788): 17[73]. Type locality: “fluviis Europae” [European rivers].

Types: Not traced.

National conservation status: Least Concern (LC) (IUCN criteria) (Dataset 2).

Distribution: European Russia (water bodies of Baltic, Black, Azov, and Caspian Sea drainage basins), western Urals; Ural River in Russia and Kazakhstan; countries of Northern, Eastern and Western Europe[3].

Comments: Here, we follow the traditional concept of this species[6,70,71]. The lists of synonyms are given by Graf[6] and Klishko et al.[70].

Supplementary References

1. Vinarski, M. V. & Kantor, Y. I. *Analytical catalogue of fresh and brackish water molluscs of Russia and adjacent countries* (A. N. Severtsov Institute of Ecology and Evolution of RAS, 2016).
2. Linnaeus, C. *Systema Naturae per Regna Tria Naturae, Secundum Classes, Ordines, Genera, Species, cum Characteribus, Differentiis, Synonymis, locis 1 (10th ed.)* (Laurentii Salvii, 1758).
3. Lopes-Lima, M. et al. Conservation status of freshwater mussels in Europe: state of the art and future challenges. *Biological Reviews* **92**, 572–607; DOI:10.1111/brv.12244 (2017).
4. *Mongolian Red Book* (Ministry of Environment and Green Development of Mongolia, 2013).
5. Val’kova, S.A. & Kashulin, N.A. Features of the structure and functioning of benthic communities in conditions of heat pollution. *Proceedings of the Kola Scientific Center of the RAS: Applied Ecology of the North* **16**, 94–103 (2013).
6. Graf, D. L. Palearctic freshwater mussel (Mollusca: Bivalvia: Unionoida) diversity and the Comparatory Method as a species concept. *Proceedings of the Academy of Natural Sciences of Philadelphia* **156**, 71–89; DOI:10.1635/0097-3157(2007)156[71:PFMMBU]2.0.CO;2 (2007).
7. Klishko, O. K., Lopes-Lima, M., Bogan, A. E., Matafonov, D. V. & Froufe, E. Morphological and molecular analyses of Anodontinae species (Bivalvia, Unionidae) of Lake Baikal and Transbaikalia. *PloS ONE* **13**, e0194944; DOI:10.1371/journal.pone.0194944 (2018).
8. Rossmässler, E. A. *Iconographie der Land- und Süsswasser-Mollusken, mit vorzüglicher Berücksichtigung der europäischen noch nicht abgebildeten Arten. Bd. 1* (Arnoldische Buchhandlung, 1835).
9. Moskvicheva, I. M. Molluscs of the subfamily Anodontinae (Bivalvia, Unionida) in the Amur and Marine Territory Basin. *Zoologicheskii Zhurnal* **52**, 822–834 (1973).
10. Bogatov, V.V. & Starobogatov, Y. I. Anodontinae (Bivalvia) in the Amur River basin. *Zoologicheskii Zhurnal* **75**, 972–977 (1996).

11. Moskvicheva, I. M. Unionoidea (Bivalvia) of the Amur and the Marine Territory Basin. *Zoologicheskii Zhurnal* **52**, 1458–1471 (1973).
12. Zatravkin, M. N. & Bogatov, V. V. Large bivalve molluscs in fresh and brackish waters of the Far East of the USSR: Keys (Far Eastern Branch of the USSR Academy of Sciences, 1987).
13. Qian, Z., Fang, Y. & He, J. Description of two new freshwater mussels from China. *Shell Discoveries* **1**, 32–33 (2015).
14. Zatravkin, M. N. Unionoidea in the fauna of the USSR and their role as intermediate hosts and eliminators of trematods. *Molluscs: Systematics, Ecology and Distribution* **7**, 40–44 (1983).
15. Middendorff, A. T. v. Wirbellose Thiere: Annulaten. Echinodermen. Insecten. Krebse. Mollusken. Parasiten. *Reise in den Äussersten Norden und Osten Sibiriens, Zoologie* **2**, 163–508 (1851).
16. Lea, I. Descriptions of five new species of Unionidae and one *Paludina* of the United States. *Proceedings of the Academy of Natural Sciences of Philadelphia* **19**, 81 (1867).
17. Zhadin, V. I. Fam. Unionidae. *Faune de l'URSS, Mollusques* **4**, 1–170 (1938).
18. Suzuki, K. New form of *Anodonta* from Hokkaido and Karahuto. *Venus* **9**, 129–144 (1939).
19. Zatravkin, M. N. & Starobogatov, Y. I. New species of the superfamily Unionoidea (Bivalvia, Unioniformes) from the Soviet Far East. *Zoologicheskii Zhurnal* **63**, 1785–1791 (1984).
20. Bogatov, V.V. & Zatravkin, M. N. New species of the order Unioniformes (Mollusca, Bivalvia) from the south of the Soviet Far East. *Trudy Zoologicheskogo Instituta* **187**, 155–168 (1988).
21. Bogatov, V. V. & Starobogatov, Y. I. Anodontinae (Bivalvia) in eastern and southern Primorye. *Zoologicheskii Zhurnal* **75**, 1326–1335 (1996).
22. Sayenko, E.M. & Bogatov, V.V. A new species of the genus *Beringiana* (Bivalvia, Unionidae). *Zoologicheskii Zhurnal* **77**, 1414–1418 (1998).
23. Labay, V. S. & Shulga, O. P. Two new species and a new subspecies of large Bivalvia (Unionidae) from fresh waters of Sakhalin Island. *Ruthenica* **9**, 77–80 (1999).
24. Bogatov, V. V., Sayenko, E. M. & Starobogatov, Y. I. Anodontine bivalves of the genus *Kunashiria* Starabogatov from the southern Kurile islands, with descriptions of two new species. *Ruthenica* **9**, 57–62 (1999).
25. Bogatov, V. V. & Starobogatov Y. I. Anodontinae (Bivalvia) of the genus *Beringiana*. *Zoologicheskii Zhurnal* **80**, 26–31 (2001).
26. Williams, J. D. et al. A revised list of the freshwater mussels (Mollusca: Bivalvia: Unionida) of the United States and Canada. *Freshwater Mollusk Biology and Conservation* **20**, 33–59 (2017).
27. Lindholm, W. A. *Anodonta arcaeformis* Heude im Süd-Ussuri-Gebiet. *Archiv für Molluskenkunde* **57**, 137–139 (1925).
28. Martynov, A.V. & Chernyshev, A.V. New and rare species of freshwater bivalves from the Soviet Far East. *Zoologicheskii Zhurnal* **71**, 18–23 (1992).
29. Bogatov, V. V., & Prozorova, L. A. Taxonomy and diversity of freshwater bivalve mollusks (Bivalvia) of China (based on analysis of the catalog by He and Zhuang, 2013). *Biology Bulletin* **44**, 922–940; DOI:10.1134/S1062359017080040 (2017).
30. Lee, J. H. *Systematic study of Korean unionids (Bivalvia: Unionidae) based on morphological and molecular data. PhD dissertation* (Kyungpook National University, 2017).
31. Graf, D. L. & Cummings, K. S. The Freshwater Mussels (Unionoida) of the World (and other less consequential bivalves), updated 9 August 2018. MUSSEL Project Web Site, http://www.mussel-project.net/ (2018).
32. Leach, W. E*. The Zoological Miscellany being descriptions of new, or interesting animals. Vol. 1* (Printed by B. McMillan for E. Nodder & Son, 1814).
33. Do, V. T, Tuan, L. Q. & Bogan, A. E. Freshwater mussels (Bivalvia: Unionida) of Vietnam: Diversity, distribution, and conservation status. *Freshwater Mollusk Biology and Conservation* **21**, 1–18 (2018).

34. Prozorova, L. A., Bogatov, V. V. & Sayenko, E. M. New data on the freshwater mollusk fauna of Sakhalin Island. *Flora and fauna of Sakhalin Island: Materials of International Sakhalin Project* **1**, 138–144 (2004).
35. Klishko, O. K., Lopes-Lima, M., Froufe, E. & Bogan, A. E. Are *Cristaria herculea* (Middendorff, 1847) and *Cristaria plicata* (Leach, 1815) (Bivalvia, Unionidae) separate species? *ZooKeys* **438**, 1–15; DOI:10.3897/zookeys.438.7493 (2014).
36. Klishko, O. K., Lopes-Lima, M., Froufe, E., Bogan, A. E. & Abakumova, V. Y. Systematics and distribution of *Cristaria plicata* (Bivalvia, Unionidae) from the Russian Far East. *ZooKeys* **580**, 13–27; DOI:10.3897/zookeys.580.7588 (2016).
37. Wu, R. W. et al. Testing the utility of DNA barcodes and a preliminary phylogenetic framework for Chinese freshwater mussels (Bivalvia: Unionidae) from the middle and lower Yangtze River. *PloS ONE* **13**, e0200956; DOI:10.1371/journal.pone.0200956 (2018).
38. Brandt, R. A. M. The non-marine aquatic mollusca of Thailand. *Archiv für Molluskenkunde* **105**, 1–423 (1974).
39. Nahok, B., Tumpeesuwan, C., Srifa, A. & Tumpeesuwan, S. Freshwater molluscan assemblages in upper part of Choen River Basin, Northeastern Thailand. *Tropical Natural History* **17**, 11–24 (2017).
40. Martens, E. v. Uebersicht über die von den Herren Dr. Fr. Hilgendorf und Dr. W. Dönitz in Japan gesammelten Binnenmollusken. *Sitzungs-Berichte der Gesellschaften naturforschender Freunde zu Berlin* **1877**, 97–123 (1877).
41. Modell, H. Die Anodontinae, Ortm. emend. (Najad., Mollusca). *Jenaische Zeitschrift für Naturwissenschaft herausgegeben von der Medizinisch-naturwissenschaftlichen Gesellschaft zu Jena* **78**, 58–100 (1945).
42. Bespalaya, Y. V. et al. DNA barcoding reveals invasion of two cryptic *Sinanodonta* mussel species (Bivalvia: Unionidae) into the largest Siberian river. *Limnologica* **69**, 94–102; DOI:10.1016/j.limno.2017.11.009 (2018).
43. Kondakov, A. V. et al. DNA analysis of a non-native lineage of *Sinanodonta woodiana* species complex (Bivalvia: Unionidae) from Middle Asia supports the Chinese origin of the European invaders. *Zootaxa* **4462**, 511–522; DOI:10.11646/zootaxa.4462.4.4 (2018).
44. Schrenck, L. v. Mollusken des Amur-Landes und des Nordjapanischen Meers. *Reisen und Forschungen im Amur-Lande in den Jahren 1854-1856* **2**, 260–976 (1867).
45. Lea, I. *A synopsis of the family Unionidae (4th ed., very greatly enlarged and improved)* (Philadelphia, 1870).
46. Sayenko, E. M., Soroka, M. & Kholin, S. K. Comparison of the species *Sinanodonta amurensis* Moskvicheva, 1973 and *Sinanodonta primorjensis* Bogatov et Zatrawkin, 1988 (Bivalvia: Unionidae: Anodontinae) in view of variability of the mitochondrial DNA cox1 gene and conchological features. *Biology Bulletin* **44**, 266–276; DOI:10.1134/S1062359017030086 (2017).
47. Fischer, P. Catalogue et distribution geographique des mollusques terrestres, fluviatiles & marins d'une partie de l'Indo-Chine (Siam, Laos, Cambodge, Conchichine, Annam, Tonkin). *Bulletin (Société d'histoire naturelle d'Autun)* **4**, 87–276 (1891).
48. Lea, I. Observations on the Naïades; and descriptions of new species of that and other families. *Transactions of the American Philosophical Society* **5**, 23–119, pls. 1–19 (1834).
49. Bolotov, I. N. et al. Spreading of the Chinese pond mussel, *Sinanodonta woodiana*, across Wallacea: One or more lineages invade tropical islands and Europe. *Biochemical Systematics and Ecology* **67**, 58–64; DOI:10.1016/j.bse.2016.05.018 (2016).
50. Konečný, A. et al. Modelling the invasion history of *Sinanodonta woodiana* in Europe: Tracking the routes of a sedentary aquatic invader with mobile parasitic larvae. *Evolutionary Applications* **11**, 1975–1989; DOI:10.1111/eva.12700 (2018).
51. Vikhrev, I. V. et al. A tropical biodiversity hotspot under the new threat: Discovery and DNA barcoding of the invasive Chinese pond mussel *Sinanodonta woodiana* in Myanmar. *Tropical Conservation Science* **10**, 1–11; DOI:10.1177/1940082917738151 (2017).

52. Huang, X. C. et al. Towards a global phylogeny of freshwater mussels (Bivalvia: Unionida): Species delimitation of Chinese taxa, mitochondrial phylogenomics, and diversification patterns. *Molecular Phylogenetics and Evolution* **130**, 45–59; DOI:10.1016/j.ympev.2018.09.019 (2019).
53. Simpson, C. T. Synopsis of the naiades, or pearly fresh-water mussels. *Proceedings of the United States National Museum* **22**, 501–1044 (1900).
54. Tomlin, J. R. le B. Some preoccupied generic names – II. *Journal of Molluscan Studies* **19**, 22–24; DOI:10.1093/oxfordjournals.mollus.a064001 (1930).
55. Griffith, E. & Pidgeon, E. *The Mollusca and Radiata, arranged by the Baron Cuvier, with supplementary additions to each order* (Printed for G.B. Whittaker, 1833).
56. Simpson, C. T. New and unfigured Unionidae. *Proceedings of the Academy of Natural Sciences of Philadelphia* **52**, 74–86 (1900).
57. Moskvicheva, I. M. & Starobogatov, Y. I. On the East Asian *Potomida*-like Unionidae (Bivalvia). *Bulletin of Moscow Society of Naturalists, Biological Series* **78**, 21–37 (1973).
58. Bogatov, V. V. Pearl mussels of the subfamily Nodulariinae (Bivalvia, Unionidae) in the Amur River Basin. *Zoologicheskii Zhurnal* **91**, 393–403 (2012).
59. Sayenko, E. M. & Kholin, S. K. New data on rare pearl mussels in the genus *Middendorffinaia* (Bivalvia: Unionidae: Nodulariinae). in *VIII Far Eastern Conference on Nature Reserves Management and Studies. Vol. 2* (ed. Starchenko, V.M.) 32–36 (Far Eastern Branch of the Russian Academy of Sciences, 2007).
60. Bogatov, V. V. The first *Middendorffinaia* (Bivalvia, Unionoidea) in Okhotsk Region. *Zoologicheskii Zhurnal* **79**, 862–862 (2000).
61. Klishko, O. K., Lopes-Lima, M., Froufe, E. & Bogan, A. E. Solution of taxonomic status of *Unio mongolicus* Middendorff, 1851 (Bivalvia: Unionidae) from the type locality in Transbaikalia and history of its taxonomy. *Ruthenica* **29**, 55–70 (2019).
62. Mousson, A. Coquilles terrestres et fluviatiles recueillies par M. Louis Graeser, dans le bassin de l'Amour, et détérminées. *Journal de Conchyliologie* **35**, 10–32 (1887).
63. Westerlund, C. A. Malacozoa Acephala. Fauna der in der Paläarctischen Region (Europa, Kaukasien, Sibirien, Turan, Persien, Kurdistan, Armenien, Mesopotamien, Kleinasien, Syrien, Arabien, Egypten, Tripolis, Tunesien, Algerien und Marocco). *Lebenden Binnenconchylien* **2**, 1–319 (1890).
64. Westerlund, C. A. Beitrage zur Molluskenfauna Russlands. *Annuaire du Musée zoologique de l'Académie des sciences de St. Pétersbourg* **1897**, 117–143 (1897).
65. Haas, F. Die Unioniden. *Systematisches Conchylien-Cabinet von Martini und Chemnitz* **9** (pt. 2, h. 41), 1–16, pls. 1–6 (1910).
66. Bogatov, V. V. & Starobogatov, Y. I. Pearly mussels from southern Primorye. *Zoologicheskii Zhurnal* **71**, 132–136 (1992).
67. Bogatov, V. V. New data on Unioniformes of Sakhalin Island. *Byulleten Dalnevostochnogo Malakologicheskogo Obshchestva* **5**, 71–77 (2001).
68. Klishko, O. K., Lopes-Lima, M., Froufe, E., Bogan, A. E. & Abakumova, V. Y. Unravelling the systematics of *Nodularia* (Bivalvia, Unionidae) species from eastern Russia. *Systematics and Biodiversity* **16**, 287–301; DOI:10.1080/14772000.2017.1383527 (2018).
69. Liu, X. et al. Genetic structure and diversity of *Nodularia douglasiae* (Bivalvia: Unionida) from the middle and lower Yangtze River drainage. *PloS ONE* **12**, e0189737; DOI:10.1371/journal.pone.0189737 (2017).
70. Klishko, O. et al. Taxonomic reassessment of the freshwater mussel genus *Unio* (Bivalvia: Unionidae) in Russia and Ukraine based on morphological and molecular data. *Zootaxa* **4286**, 93–112; DOI:10.11646/zootaxa.4286.1.4 (2017).
71. Araujo, R., Buckley, D., Nagel, K. O., García-Jiménez, R. & Machordom, A. Species boundaries, geographic distribution and evolutionary history of the Western Palaearctic freshwater mussels *Unio* (Bivalvia: Unionidae). *Zoological Journal of the Linnean Society* **182**, 275–299; DOI:10.1093/zoolinnean/zlx039 (2017).

72. Prié, V., Puillandre, N. & Bouchet, P. Bad taxonomy can kill: molecular reevaluation of *Unio mancus* Lamarck, 1819 (Bivalvia: Unionidae) and its accepted subspecies. *Knowledge and Management of Aquatic Ecosystems* **405**, 08; DOI:10.1051/kmae/2012014 (2012).
73. Retzius A.J. *Dissertatio historico-naturalis sistens nova Testaceorum genera* (Berling, 1788).
74. Andreeva, S. I., Vinarski, M. V. & Karimov, A. V. The first record of *Unio* species (Bivalvia: Unionidae) in the Irtysh River basin (Western Siberia, Russia). *Mollusca* **27**, 87–91 (2009).
