# Supplementary material for: Integrative taxonomy, biogeography and conservation of freshwater mussels (Unionidae) in Russia
Source: Sci Rep. 2020 Feb 20;10:3072. doi: 10.1038/s41598-020-59867-7 (PMC7033218; doi:10.1038/s41598-020-59867-7)
Supplement: Supplementary file 3 — Dataset 2 [file 41598_2020_59867_MOESM3_ESM.pdf]

Please cite this work as follows: Ivan N. Bolotov, Alexander V. Kondakov, Ekaterina S. Konopleva, Ilya V. Vikhrev, Olga V. Aksenova, Andrey S. Aksenov, Yulia V. Bespalaya, Alexey V. Borovskoy, Petr P. Danilov, Gennady A. Dvoryankin, Mikhail Y. Gofarov, Mikhail B. Kabakov, Olga K. Klishko, Yulia S. Kolosova, Artem A. Lyubas, Alexander P. Novoselov, Dmitry M. Palatov, Grigory N. Savvinov, Nikolay M. Solomonov, Vitaly M. Spitsyn, Svetlana E. Sokolova, Alena A. Tomilova, Elsa Froufe, Arthur E. Bogan, Manuel Lopes-Lima, Alexander A. Makhrov & Maxim V. Vinarski (2020) **Integrative taxonomy, biogeography and conservation of freshwater mussels (Unionidae) in Russia**. *Scientific Reports*.

Dataset 2. National Red List of freshwater mussel species (Bivalvia: Unionidae) of Russia

Taxonomic classification, distribution and conservation status of freshwater mussel (Unionida) species in Russia. Abbreviations: CR, critically endangered; DD, data deficient; EN, endangered; NA, not applicable; NE, not evaluated; VU, vulnerable.

Taxon	Conservation status		% of global population in Russia
	National	Global	
Family Unionidae			
Subfamily Unioninae			
Tribe Anodontini			
***Anodonta anatina* (Linnaeus, 1758)**	LC	LC	65
***Anodonta cygnea* (Linnaeus, 1758)**	LC	LC	35
***Pseudanodonta complanata* (Rossmässler, 1835)**	LC	VU	35
Tribe Cristariini			
***Amuranodonta kijaensis* Moskvicheva, 1973**	LC	NE	>50
***Beringiana beringiana* (Middendorff, 1851)**	LC	LC	40
***Buldowskia shadini* (Moskvicheva, 1973)**	LC	NE	>50
***Buldowskia suifunica* (Lindholm, 1925)**	VU	NE	>75
***Cristaria plicata* (Leach, 1814)**	LC	DD	20
***Sinanodonta lauta* (Martens, 1877)**	EN	NE	30
***Sinanodonta schrenkii* (Lea, 1870)**	LC	NE	>50
Tribe Lanceolariini			
***Lanceolaria grayii* (Griffith & Pidgeon, 1833)**	LC	DD	20
Tribe Unionini			
***Middendorffinaia mongolica* (Middendorff, 1851)**	LC	NE	>50
***Nodularia douglasiae* (Griffith & Pidgeon, 1833)**	LC	LC	20
***Unio crassus* Retzius, 1788**	LC	EN	45
***Unio pictorum* (Linnaeus, 1758)**	LC	LC	40
***Unio tumidus* Retzius, 1788**	LC	LC	40

SCIENTIFIC NAME

***Anodonta anatina* (Linnaeus, 1758)**

TAXONOMIC NOTES

The taxonomic history of this species in Russia is complex, because multiple ecophenotypes were previously accepted as separate, valid species (Starobogatov et al. 2004; Bogatov and Kijashko 2016; Vinarski and Kantor 2016). However, all these nominal taxa from European Russia and Siberia were recently considered synonyms of the widespread *Anodonta anatina* based on morphological and multi-locus molecular data (Graf 2007; Lopes-Lima et al. 2017; Klishko et al. 2018; this study).

RED LIST ASSESSMENT

Least Concern (LC).

JUSTIFICATION

It is the most widespread freshwater mussel in Russia with an estimated EOO of more than 10,000,000 km^2. No major threats are known for the species; therefore, this species is here listed as Least Concern (LC) in Russia.

GEOGRAPHIC RANGE

The species occurs across European Russia, Urals and east to Siberia eastward to the Lena River basin (Lopes-Lima et al. 2017; Klishko et al. 2018). A local introduced population has been recorded in a channel artificially heated by the Kola Nuclear Power Plant in Lake Imandra, Kola Peninsula (Valkova and Kashulin 2013).

HABITAT AND ECOLOGY

It is considered a habitat generalist species, being found in a variety of rivers, streams, channels, lakes, and ponds with different kinds of substrate. In Russia, larvae release occurs from March to Early May in the Volga Delta (Antonova 1991), and from May to Early June in the Oka River, Volga Basin (Zhadin 1938). The fish hosts are *Perca fluviatilis* Linnaeus, 1758, *Rutilus* spp., and other widespread fish species (O. Klishko, pers. obs.).

THREATS

There are no known major threats to this species in Russia.

CONSERVATION ACTIONS

No major conservation actions are required for this species in Russia.

RESEARCH NEEDS

The species needs research on its population trends, detailed distribution and phylogeographic structure to delineate the primary evolutionary significant units throughout Russia.

REFERENCES

Antonova, A.A. Connection of reproductive cycles of the Unionidae of the Volga Delta with environmental factors. *Proceedings of the Zoological Institute, Academy of Sciences of the USSR* **228**, 12–29 (1991).

Bogatov, V. V. & Kijashko, P. V. Class of bivalve mollusks – Bivalvia Linnaeus, 1758. in *Key of freshwater zooplankton and zoobenthos of the fresh water in European Russia. Vol. 2* (Alekseev, V. R. & Tsalolikhin, S. J., eds.) 285–334 (KMK Scientific Press Ltd., 2016).

Graf, D. L. Palearctic freshwater mussel (Mollusca: Bivalvia: Unionoida) diversity and the Comparatory Method as a species concept. *Proceedings of the Academy of Natural Sciences of Philadelphia* **156**, 71–89; DOI:10.1635/0097-3157(2007)156[71:PFMMBU]2.0.CO;2 (2007).

Klishko, O. K., Lopes-Lima, M., Bogan, A. E., Matafonov, D. V. & Froufe, E. Morphological and molecular analyses of Anodontinae species (Bivalvia, Unionidae) of Lake Baikal and Transbaikalia. *PloS ONE* **13**, e0194944; DOI:10.1371/journal.pone.0194944 (2018).

Lopes-Lima, M. et al. Conservation status of freshwater mussels in Europe: state of the art and future challenges. *Biological Reviews* **92**, 572–607; DOI:10.1111/brv.12244 (2017).

Starobogatov, Y. I., Prozorova, L. A., Bogatov, V. V. & Saenko, E. M. Mollusks. in Key to freshwater invertebrates of Russia and adjacent lands. Vol. 6. Molluscs, polychaetes, nemerteans (Tsalolikhin, S. Y., ed.). 9–491 (Nauka, 2004).

Valkova, S. A. & Kashulin, N. A. Features of the structure and functioning of benthic communities in conditions of heat pollution. *Proceedings of the Kola Scientific Center of the RAS: Applied Ecology of the North* **16**, 94–103 (2013).

Vinarski, M. V. & Kantor, Y. I. Analytical catalogue of fresh and brackish water molluscs of Russia and adjacent countries (A. N. Severtsov Institute of Ecology and Evolution of RAS, 2016).

Zhadin, V. I. Fam. Unionidae. *Faune de l'URSS, Mollusques* **4**, 1–170 (1938).

SCIENTIFIC NAME

Anodonta cygnea **(Linnaeus, 1758)**

TAXONOMIC NOTES

Here, we follow the traditional concept of this species (Graf 2007; Lopes-Lima et al. 2017).

RED LIST ASSESSMENT

Least Concern (LC).

JUSTIFICATION

This species is widespread and common throughout European Russia. It has an estimated EOO more than 2,000,000 km^2. Therefore, this species is here listed as Least Concern (LC) in Russia.

GEOGRAPHIC RANGE

European Russia (water bodies of the Baltic, Black, Azov, and Caspian Sea drainage basins).

HABITAT AND ECOLOGY

This species generally occurs in lentic habitats of rivers, channels, and lakes with different kinds of substrate. There is no data about the hosts, or other aspects of its life cycle in Russia, although the species is known to use a wide variety of hosts in others parts of its distribution (Lopes-Lima et al. 2017).

THREATS

There are no known major threats to this species in Russia.

CONSERVATION ACTIONS

No major conservation actions are required for this species in Russia.

RESEARCH NEEDS

The species needs research on its detailed distribution and population trends, basic life-history traits such as age of maturity, life span, growth and fish hosts use, as well as on its phylogeographic structure to delineate the primary evolutionary significant units.

REFERENCES

Graf, D. L. Palearctic freshwater mussel (Mollusca: Bivalvia: Unionoida) diversity and the Comparatory Method as a species concept. *Proceedings of the Academy of Natural Sciences of Philadelphia* **156**, 71–89; DOI:10.1635/0097-3157(2007)156[71:PFMMBU]2.0.CO;2 (2007).

Lopes-Lima, M. et al. Conservation status of freshwater mussels in Europe: state of the art and future challenges. *Biological Reviews* **92**, 572–607; DOI:10.1111/brv.12244 (2017).

SCIENTIFIC NAME

***Pseudanodonta complanata* (Rossmässler, 1835)**

TAXONOMIC NOTES

Here, we follow the traditional concept of this species (Graf 2007; Lopes-Lima et al. 2017).

RED LIST ASSESSMENT

Least Concern (LC).

JUSTIFICATION

This species is widespread and common throughout European Russia, with the exception of northern areas. It has an estimated EOO more than 2,000,000 km^2. The population trends are unknown. Therefore, this species is here listed as Least Concern (LC) in Russia.

GEOGRAPHIC RANGE

European Russia (water bodies of the Baltic, Black, Azov, and Caspian Sea drainage basins). Not known from the Ural River (Russia and Kazakhstan).

HABITAT AND ECOLOGY

The species is found in slowly flowing rivers and lakes with different kinds of bottom substrate. Larvae release occurs in June (Zhadin 1938), but there is no information about the fish hosts in Russia.

THREATS

There are no known major threats to this species in Russia.

CONSERVATION ACTIONS

No major conservation actions are required for this species in Russia.

RESEARCH NEEDS

The species needs research on its detailed distribution and population trends, basic life-history traits such as age of maturity, life span, growth and fish hosts use, as well as on its phylogeographic structure to delineate the primary evolutionary significant units. Given that the species is declining dramatically in other European countries, the long-term monitoring is needed in order to evaluate the population trends of Russian populations.

REFERENCES

Graf, D. L. Palearctic freshwater mussel (Mollusca: Bivalvia: Unionoida) diversity and the Comparatory Method as a species concept. *Proceedings of the Academy of Natural Sciences of Philadelphia* **156**, 71–89; DOI:10.1635/0097-3157(2007)156[71:PFMMBU]2.0.CO;2 (2007).

Lopes-Lima, M. et al. Conservation status of freshwater mussels in Europe: state of the art and future challenges. *Biological Reviews* **92**, 572–607; DOI:10.1111/brv.12244 (2017).

Zhadin, V. I. Fam. Unionidae. *Faune de l'URSS, Mollusques* **4**, 1–170 (1938).

SCIENTIFIC NAME

***Amuranodonta kijaensis* Moskvicheva, 1973**

TAXONOMIC NOTES

A single *Amuranodonta* species having an elongated, narrow shell is known from the Amur Basin. Moskvicheva (1973) introduced two nominal *Anemina*-like taxa with an elongated shell: *Amuranodonta kijaensis* and *A. parva*. Acting as the First Revisers, we have chosen *Amuranodonta kijaensis* as the valid name for this species. Six additional nominal taxa having the elongated shell were described from the Amur Basin (Moskvicheva 1973; Zatravkin and Bogatov 1987; Bogatov and Starobogatov 1996; Qian et al. 2015). These taxa are considered junior synonyms of *Amuranodonta kijaensis*.

RED LIST ASSESSMENT

Least Concern (LC).

JUSTIFICATION

The species is common throughout the Amur Basin with an estimated EOO of more than 1,000,000 km^2. No major threats are known, therefore, the species is here listed as Least Concern (LC) in Russia.

GEOGRAPHIC RANGE

This species inhabits Amur Basin and Lake Arey in Transbaikalia.

HABITAT AND ECOLOGY

It is generally found in lakes and slowly flowing river sections in silty, clay and sandy substrates. The age of maturity is 2 years and maximum life span is 8 years (usually up to 6-7 years) (O. Klishko, pers. obs.). Larvae release occurs from May to August; the host fish is *Rhodeus sericeus* (Pallas, 1776); glochidial development on fins and body of the host fish takes 2-3 weeks (Klishko 2012).

THREATS

There are no known threats to this species in Russia, but abundance of some local populations may be decreasing due to extreme floods, water pollution, and habitat degradation.

CONSERVATION ACTIONS

No major conservation actions are required for this species in Russia.

RESEARCH NEEDS

The species needs research on its phylogeographic structure to delineate the primary evolutionary significant units throughout the Amur Basin.

REFERENCES

Moskvicheva, I. M. Molluscs of the subfamily Anodontinae (Bivalvia, Unionida) in the Amur and Marine Territory Basin. *Zoologicheskii Zhurnal* **52**, 822–834 (1973).

Bogatov, V.V. & Starobogatov, Y. I. Anodontinae (Bivalvia) in the Amur River basin. *Zoologicheskii Zhurnal* **75**, 972–977 (1996).

Klishko, O.K. Some data on reproductive biology of the freshwater mussels (Margaritiferidae, Unionidae) and their relationships with bitterlings (Cyprinidae) in Transbaikalye. *The Bulletin of the Russian Far East Malacological Society* **15/16**, 31–55 (2012).

Qian, Z., Fang, Y. & He, J. Description of two new freshwater mussels from China. *Shell Discoveries* **1**, 32–33 (2015).

Zatravkin, M. N. & Bogatov, V. V. *Large bivalve molluscs in fresh and brackish waters of the Far East of the USSR: Keys* (Far Eastern Branch of the USSR Academy of Sciences, 1987).

SCIENTIFIC NAME

Beringiana beringiana **(Middendorff, 1851)**

TAXONOMIC NOTES

This variable and widespread species has multiple names. *Beringiana beringiana* is the oldest available name. The majority of younger available names represent morphological varieties with minute differences in shell shape and convexity that were thought to be endemics of certain lakes, streams, and rivers (Zatravkin and Starobogatov 1984; Zatravkin and Bogatov 1987; Bogatov and Zatravkin 1988; Bogatov and Starobogatov 1996; Sayenko and Bogatov 1998; Labay and Shulga 1999; Bogatov et al. 1999; Bogatov and Starobogatov 2001). We place these names into the synonymy of *Beringiana beringiana* based on conchological similarity, multi-locus molecular data, and geographic evidence. In contrast, *Beringiana georgiensis* Bogatov & Starobogatov, 2001 (type locality: Fort George, Oregon, USA) (Bogatov and Starobogatov 2001) belongs to another genus and is a junior synonym of *Anodonta oregonensis* Lea, 1838 (based on molecular sequences of the topotypes from the Columbia River basin; our unpublished data).

RED LIST ASSESSMENT

Least Concern (LC).

JUSTIFICATION

This widespread species has an estimated EOO more than 3,000,000 km^2. No major threats are known for this species, therefore, the species is here listed as Least Concern (LC) in Russia.

GEOGRAPHIC RANGE

This species inhabits rivers east of the Lena Basin (Kolyma Highlands, Chukotka, and Kamchatka), coastal rivers of the Japan and Okhotsk Sea drainage basins northeast of Vladivostok (from the Kievka River), Kuriles and Sakhalin islands.

HABITAT AND ECOLOGY

This species is found in lentic habitats such as lakes, ponds and slowly flowing river sections on silty and clay substrates. Larvae release occurs from June to August (Sayenko et al. 2001). The host fishes are *Gasterosteus aculeatus* Linnaeus, 1758, *Pungitius pungitius* (Linnaeus, 1758), *Hypomesus olidus* (Pallas, 1814), *Oncorhynchus kisutch* (Walbaum, 1792), *O. nerka* (Walbaum, 1792), *Salvelinus malma* (Walbaum, 1792), *S. albus* Glubokovsky, 1977, and *S. leucomaenis* (Pallas, 1814) (Sayenko et al. 2001).

THREATS

There are no known threats to this species in Russia.

CONSERVATION ACTIONS

No major conservation actions are required for this species in Russia.

RESEARCH NEEDS

The species needs research on its population trends, detailed distribution and basic life-history traits such as age of maturity, life span, and growth, as well as on its phylogeographic structure to delineate the primary evolutionary significant units throughout Asiatic Russia.

REFERENCES

Bogatov, V. V. & Starobogatov Y. I. Anodontinae (Bivalvia) of the genus *Beringiana*. *Zoologicheskii Zhurnal* **80**, 26–31 (2001).

Bogatov, V. V., Sayenko, E. M. & Starobogatov, Y. I. Anodontine bivalves of the genus *Kunashiria* Starabogatov from the southern Kurile islands, with descriptions of two new species. *Ruthenica* **9**, 57–62 (1999).

Bogatov, V. V. & Starobogatov, Y. I. Anodontinae (Bivalvia) in eastern and southern Primorye. *Zoologicheskii Zhurnal* **75**, 1326–1335 (1996).

Bogatov, V.V. & Zatravkin, M. N. New species of the order Unioniformes (Mollusca, Bivalvia) from the south of the Soviet Far East. *Trudy Zoologicheskogo Instituta* **187**, 155–168 (1988).

Labay, V. S. & Shulga, O. P. Two new species and a new subspecies of large Bivalvia (Unionidae) from fresh waters of Sakhalin Island. *Ruthenica* **9**, 77–80 (1999).

Sayenko, E.M. & Bogatov, V.V. A new species of the genus *Beringiana* (Bivalvia, Unionidae). *Zoologicheskii Zhurnal* **77**, 1414–1418 (1998).

Saenko E. M., Shed'ko M. B., Kholin S. K. Morphology and feature of bionomics of glochidia mollusks of the genus *Beringiana* (Bivalvia, Unionidae) of Kamchatka and Northern Kuriles. *Vestnik zoologii* **35**, 59—68 (2001).

Zatravkin, M. N. & Bogatov, V. V. *Large bivalve molluscs in fresh and brackish waters of the Far East of the USSR: Keys* (Far Eastern Branch of the USSR Academy of Sciences, 1987).

Zatravkin, M. N. & Starobogatov, Y. I. New species of the superfamily Unionoidea (Bivalvia, Unioniformes) from the Soviet Far East. *Zoologicheskii Zhurnal* **63**, 1785–1791 (1984).

SCIENTIFIC NAME

***Buldowskia shadini* (Moskvicheva, 1973)**

TAXONOMIC NOTES

A single *Buldowskia* species with ovate shell is known from the Amur Basin. Moskvicheva (1973) introduced this species under three different names. Acting as the First Revisers, we have chosen *Buldowskia shadini* as the valid name for this species. Other nominal *Anemina*-like taxa with ovate shell described from the Amur Basin (Moskvicheva 1973; Bogatov and Starobogatov 1996) are considered synonyms of this species.

RED LIST ASSESSMENT

Least Concern (LC).

JUSTIFICATION

The species is common throughout the Amur Basin. It has an estimated EOO more than 1,000,000 km^2. No major threats are known, therefore, the species is here listed as Least Concern (LC) in Russia.

GEOGRAPHIC RANGE

This species inhabits Amur Basin and Lake Arey in Transbaikalia.

HABITAT AND ECOLOGY

It is generally found in lakes and slowly flowing river sections on silty, clay and sandy-clay substrates. The age of maturity is 2 years and maximum life span is 12 years (usually up to 7-10 years) (O. Klishko, pers. obs.). Larvae release occurs from April to May; the host fish is *Rhodeus sericeus* (Pallas, 1776) (O. Klishko, pers. obs.).

THREATS

There are no known major threats to this species in Russia, but abundance of some local populations have decreased due to extreme floods, water pollution, and by muskrat *Ondatra zibethicus* (Linnaeus, 1766) predation (O. Klishko, pers. obs.).

CONSERVATION ACTIONS

No major conservation actions are required for this species in Russia.

RESEARCH NEEDS

The species needs research on its population trends, detailed distribution and phylogeographic structure to delineate the primary evolutionary significant units throughout the Amur Basin.

REFERENCES

Moskvicheva, I. M. Molluscs of the subfamily Anodontinae (Bivalvia, Unionida) in the Amur and Marine Territory Basin. *Zoologicheskii Zhurnal* **52**, 822–834 (1973).

Bogatov, V.V. & Starobogatov, Y. I. Anodontinae (Bivalvia) in the Amur River basin. *Zoologicheskii Zhurnal* **75**, 972–977 (1996).

SCIENTIFIC NAME

***Buldowskia suifunica* (Lindholm, 1925)**

TAXONOMIC NOTES

Buldowskia Moskvicheva, 1973 was until recently considered a synonym of *Anemina* Haas, 1969 (Graf 2007). However, this clade is phylogenetically distant from the *Amuranodonta* and *Anemina* s. str. clades (this study). In addition to *Buldowskia suifunica* and *B. shadini*, this genus contains *B. flavotincta* (Martens, 1905), a lineage endemic to South Korea (Lee 2017), and some undescribed species from South Korea and Japan (M. Lopes-Lima, pers. comm., 2019).

RED LIST ASSESSMENT

Vulnerable (VU) B1ab(i,ii,iii,iv)

JUSTIFICATION

This species has an estimated EOO of less than 20,000 km^2 in Russia, being severely fragmented. There has been an observed decline in terms of its EOO, AOO, habitat extent and number of subpopulations. Therefore, the species is here listed as Vulnerable (VU) in Russia.

GEOGRAPHIC RANGE

This species is endemic to the Razdolnaya River basin and coastal rivers southwest of Vladivostok; should also be found in northeastern China (at least within the Razdolnaya Basin) and North Korea.

HABITAT AND ECOLOGY

It is generally found in lakes in silty and clay substrates. There is no knowledge about either fish hosts, or other aspects of its life cycle in Russia.

THREATS

Urban and industrial pollution can cause the extirpation of local populations, mainly near villages and cities.

CONSERVATION ACTIONS

This species would benefit from the restoration and maintenance of ecological flows, wastewater treatment and whole catchment management.

RESEARCH NEEDS

The species needs research on its basic life-history traits such as age of maturity, life span, growth and fish hosts use. Long-term surveys are needed to monitor the demographic trends of the most important populations.

REFERENCES

Graf, D. L. Palearctic freshwater mussel (Mollusca: Bivalvia: Unionoida) diversity and the Comparatory Method as a species concept. *Proceedings of the Academy of Natural Sciences of Philadelphia* **156**, 71–89; DOI:10.1635/0097-3157(2007)156[71:PFMMBU]2.0.CO;2 (2007).

Lee J. H. *Systematic study of Korean unionids (Bivalvia: Unionidae) based on morphological and molecular data. PhD dissertation* (Kyungpook National University, 2017).

SCIENTIFIC NAME

***Cristaria plicata* (Leach, 1814)**

TAXONOMIC NOTES

Here, we follow the commonly accepted concept of this species (Graf 2007; Klishko et al. 2014, 2016).

RED LIST ASSESSMENT

Least Concern (LC).

JUSTIFICATION

This species has a wide distribution range with an estimated EOO more than 1,000,000 km^2. No major threat is known for the species, therefore, this species is here listed as Least Concern (LC) in Russia.

GEOGRAPHIC RANGE

This species is widespread throughout the Amur Basin; one record is known from Tym' River, central Sakhalin (Prozorova et al. 2004).

HABITAT AND ECOLOGY

Generalist species, which is found in a variety of lakes, channels, ponds, and slowly flowing river sections with silty, clay, and sandy substrates. Life span is 13-15 years (O. Klishko, pers. obs.). Larvae release occurs from April to May (Prozorova and Sayenko 2001; Sayenko et al. 2005). The host fishes are unknown.

THREATS

There are no known threats to this species in Russia.

CONSERVATION ACTIONS

No major conservation actions are required for this species in Russia.

RESEARCH NEEDS

The species needs research on its population trends, detailed distribution and basic life-history traits such as fish hosts use, age of maturity, and growth.

REFERENCES

Graf, D. L. Palearctic freshwater mussel (Mollusca: Bivalvia: Unionoida) diversity and the Comparatory Method as a species concept. *Proceedings of the Academy of Natural Sciences of Philadelphia* **156**, 71–89; DOI:10.1635/0097-3157(2007)156[71:PFMMBU]2.0.CO;2 (2007).

Klishko, O. K., Lopes-Lima, M., Froufe, E. & Bogan, A. E. Are Cristaria herculea (Middendorff, 1847) and Cristaria plicata (Leach, 1815) (Bivalvia, Unionidae) separate species? *ZooKeys* **438**, 1–15; DOI:10.3897/zookeys.438.7493 (2014).

Klishko, O. K., Lopes-Lima, M., Froufe, E., Bogan, A. E. & Abakumova, V. Y. Systematics and distribution of Cristaria plicata (Bivalvia, Unionidae) from the Russian Far East. *ZooKeys* **580**, 13–27; DOI:10.3897/zookeys.580.7588 (2016).

Prozorova, L.A. & Sayenko, E. M. To biology of the freshwater mussel genus *Cristaria* (Bivalvia, Unionidae). *Ruthenica* **11**, 33–36 (2001).

Prozorova, L. A., Bogatov, V. V. & Sayenko, E. M. New data on the freshwater mollusk fauna of Sakhalin Island. Flora and fauna of Sakhalin Island: Materials of International Sakhalin Project 1, 138–144 (2004).

Sayenko, E. M., Pearce, T. A. & Shea, E. K. Glochidial morphology of selected species of the genera *Cristaria* Schumacher, 1817 and *Sinanodonta* Modell, 1945 (Bivalvia: Unionidae) from far Eastern Russia. *American Malacological Bulletin* **20**, 11–21 (2005).

SCIENTIFIC NAME

Sinanodonta lauta **(Martens, 1877)**

TAXONOMIC NOTES

This interesting species has a broad range in East Asia, but in Russia its native populations are known from a few coastal freshwater basins close to the boundary of North Korea (Bogatov and Starobogatov 1996). Bespalaya et al. (2018) and Kondakov et al. (2018) mentioned this species as *Sinanodonta ovata* based on the results of molecular analyses of the topotypes of this nominal taxon from the Gladkaya River. Kondakov et al. (2018) assumed that this species may have an older available name among the Japanese nominal taxa. *Sinanodonta lauta* seems to be the oldest name for this species. Two nominal taxa described from the Gladkaya River (Bogatov and Starobogatov 1996) are considered synonyms of *Sinanodonta lauta*.

RED LIST ASSESSMENT

Endangered (EN) B1ab(i,ii,iii,iv).

JUSTIFICATION

This species has an estimated EOO of less than 5,000 km^2 in Russia, being severely fragmented. There has been an observed decline in terms of its EOO, AOO, habitat extent and number of subpopulations. Therefore, the species is here listed as Endangered (EN) B1ab(i,ii,iii,iv) in Russia.

GEOGRAPHIC RANGE

Native to coastal rivers southwest of Vladivostok. A non-native population was recorded in the Yenisei River, Eastern Siberia (Bespalaya et al., 2018).

HABITAT AND ECOLOGY

It is found in slowly flowing river sections in silty and clay substrates. There is no knowledge about the fish hosts, or other aspects of its life cycle in Russia.

THREATS

The species is threatened by water pollution and habitat degradation.

CONSERVATION ACTIONS

This species would benefit from restoration and maintenance of ecological flows, wastewater treatment and whole catchment management.

RESEARCH NEEDS

The species needs research on its population trends, detailed distribution and basic life-history traits such as fish hosts use, age of maturity, life span, and growth. Long-term surveys are needed to monitor the demographic trends of the most important populations.

REFERENCES

Bespalaya, Y. V. et al. DNA barcoding reveals invasion of two cryptic *Sinanodonta* mussel species (Bivalvia: Unionidae) into the largest Siberian river. *Limnologica* **69**, 94–102; DOI:10.1016/j.limno.2017.11.009 (2018).

Bogatov, V. V. & Starobogatov, Y. I. Anodontinae (Bivalvia) in eastern and southern Primorye. *Zoologicheskii Zhurnal* **75**, 1326–1335 (1996).

Kondakov, A. V. et al. DNA analysis of a non-native lineage of *Sinanodonta woodiana* species complex (Bivalvia: Unionidae) from Middle Asia supports the Chinese origin of the European invaders. *Zootaxa* **4462**, 511–522; DOI:10.11646/zootaxa.4462.4.4 (2018).

SCIENTIFIC NAME

***Sinanodonta schrenkii* (Lea, 1870)**

TAXONOMIC NOTES

A single *Sinanodonta* species inhabits the Amur and Razdolnaya river basins. Sayenko et al. (2017) resurrected this species as *Sinanodonta amurensis* Moskvicheva, 1973. However, Kondakov et al. (2018) showed that *Sinanodonta schrenkii* (Lea, 1870) is the oldest available name for this taxon. Other nominal taxa of *Sinanodonta* described from the Amur and Razdolnaya basins are considered synonyms of this species based on conchological similarity, multi-locus molecular data, and geographic evidence (this study).

RED LIST ASSESSMENT

Least Concern (LC).

JUSTIFICATION

The species has an estimated EOO more than 1,000,000 km^2. Therefore, the species is here listed as Least Concern (LC) in Russia.

GEOGRAPHIC RANGE

This species is widespread throughout the Amur and Razdolnaya basins.

HABITAT AND ECOLOGY

Generalist species, which is found in floodplain lakes and slowly flowing river sections in silty, sandy-gravel, and clay substrates. The maximum life span is 20 years (usually up to 15-17 years) (O. Klishko, pers. obs.). The host fishes and life cycle are unknown.

THREATS

There are no major threats to this species in Russia, but abundance of some local populations could decrease due to water pollution and habitat degradation.

CONSERVATION ACTIONS

No major conservation actions are required for this species in Russia.

RESEARCH NEEDS

The species needs research on its population trends, detailed distribution and basic life-history traits such as fish hosts use, age of maturity, and growth.

REFERENCES

Kondakov, A. V. et al. DNA analysis of a non-native lineage of *Sinanodonta woodiana* species complex (Bivalvia: Unionidae) from Middle Asia supports the Chinese origin of the European invaders. *Zootaxa* **4462**, 511–522; DOI:10.11646/zootaxa.4462.4.4 (2018).

Sayenko, E. M., Soroka, M. & Kholin, S. K. Comparison of the species *Sinanodonta amurensis* Moskvicheva, 1973 and *Sinanodonta primorjensis* Bogatov et Zatrawkin, 1988 (Bivalvia: Unionidae: Anodontinae) in view of variability of the mitochondrial DNA cox1 gene and conchological features. *Biology Bulletin* **44**, 266–276; DOI:10.1134/S1062359017030086 (2017).

SCIENTIFIC NAME

***Lanceolaria grayii* (Griffith & Pidgeon, 1833)**

TAXONOMIC NOTES

A single *Lanceolaria* species inhabits the Khanka Lake, Ussuri, and Lower Amur basins. While several species-group names were introduced for this Russian lineage (Schrenck 1867; Simpson 1900; Moskvicheva 1973; Zatravkin and Starobogatov 1984), in this study it is found to be a local population of the widespread *Lanceolaria grayii*. Five nominal taxa described from Russia are considered synonyms of this species.

RED LIST ASSESSMENT

Least Concern (LC).

JUSTIFICATION

This species has a wide distribution in Russia with an estimated EOO of more than 100,000 km^2. Although local declines have been reported, no major threats are known to be affecting the whole population, therefore, the species is here listed as Least Concern (LC) in Russia.

GEOGRAPHIC RANGE

This species is recorded from Lake Khanka, Ussuri Basin and Lower Amur River.

HABITAT AND ECOLOGY

It is found in lakes and slowly flowing downstream river sections in clay and sandy substrates. There is no knowledge about the fish hosts, or other aspects of its life cycle in Russia.

THREATS

There are no known major threats to this species in Russia, but abundance of some local populations have been declining due to water pollution and habitat degradation.

CONSERVATION ACTIONS

No major conservation actions are required for this species in Russia.

RESEARCH NEEDS

The species needs research on its population trends, detailed distribution and basic life-history traits such as fish hosts use, age of maturity, life span, and growth. Long-term surveys are needed to monitor the demographic trends of the most important populations.

REFERENCES

Moskvicheva, I. M. Unionoidea (Bivalvia) of the Amur and the Marine Territory Basin. *Zoologicheskii Zhurnal* **52**, 1458–1471 (1973).

Schrenck, L. v. Mollusken des Amur-Landes und des Nordjapanischen Meers. *Reisen und Forschungen im Amur-Lande in den Jahren 1854-1856* **2**, 260–976 (1867).

Simpson, C. T. Synopsis of the naiades, or pearly fresh-water mussels. *Proceedings of the United States National Museum* **22**, 501–1044 (1900).

Zatravkin, M. N. & Starobogatov, Y. I. New species of the superfamily Unionoidea (Bivalvia, Unioniformes) from the Soviet Far East. *Zoologicheskii Zhurnal* **63**, 1785–1791 (1984).

SCIENTIFIC NAME

Middendorffinaia mongolica **(Middendorff, 1851)**

TAXONOMIC NOTES

The taxonomic history of this species in Russia is complex, because multiple ecophenotypes were previously accepted as separate, valid species (Starobogatov et al. 2004; Vinarski and Kantor 2016). However, all these nominal taxa were recently considered synonyms of the widespread *Middendorffinaia mongolica* based on morphological and multi-locus molecular data (Graf 2007; Klishko et al. 2019; this study).

RED LIST ASSESSMENT

Least Concern (LC).

JUSTIFICATION

This species has an estimated EOO more than 1,000,000 km^2. No major threats are known for this species, therefore, the species is here listed as Least Concern (LC) in Russia.

GEOGRAPHIC RANGE

This species is widespread throughout the Amur and Razdolnaya basins, coastal rivers of the Japan Sea drainage west of Nakhodka (Partizanskaya and Artemovka rivers (Sayenko and Kholin 2007) and southwest of Vladivostok up to the boundary with North Korea. Putative endemic species to the region having a rather fragmentary distribution due to its preference to specific habitats, i.e. fast flowing rivers with gravel-sandy and sandy bottom. The record from

the Kuchtuy River Basin (Okhotsk Region) (Bogatov 2000; Klishko et al. 2019) belongs to *Nodularia douglasiae*.

HABITAT AND ECOLOGY

This species is an ecological specialist to lotic habitats, being generally found in fast flowing rivers and streams with gravel-sandy and sandy bottom substrate. There is no knowledge about the fish hosts, or other aspects of its life cycle in Russia.

THREATS

There are no known major threats to this species in Russia, while river damming may cause the extirpation of local populations (e.g. in the Zeya River basin).

CONSERVATION ACTIONS

This species would benefit from the restoration and maintenance of ecological flows, wastewater treatment and whole catchment management.

RESEARCH NEEDS

The species needs research on its population trends, detailed distribution and basic life-history traits such as fish hosts use, age of maturity, life span, and growth. Long-term surveys are needed to monitor the demographic trends of the most important populations.

REFERENCES

Bogatov, V. V. The first *Middendorffinaia* (Bivalvia, Unionoidea) in Okhotsk Region. *Zoologicheskii Zhurnal* **79**, 862–862 (2000).

Graf, D. L. Palearctic freshwater mussel (Mollusca: Bivalvia: Unionoida) diversity and the Comparatory Method as a species concept. *Proceedings of the Academy of Natural Sciences of Philadelphia* **156**, 71–89; DOI:10.1635/0097-3157(2007)156[71:PFMMBU]2.0.CO;2 (2007).

Klishko, O. K., Lopes-Lima, M., Froufe, E. & Bogan, A. E. Solution of taxonomic status of *Unio mongolicus* Middendorff, 1851 (Bivalvia: Unionidae) from the type locality in Transbaikalia and history of its taxonomy. *Ruthenica* **29**, 55–70 (2019).

Sayenko, E. M. & Kholin, S. K. New data on rare pearl mussels in the genus *Middendorffinaia* (Bivalvia: Unionidae: Nodulariinae). in *VIII Far Eastern Conference on Nature Reserves Management and Studies. Vol. 2* (ed. Starchenko, V.M.) 32–36 (Far Eastern Branch of the Russian Academy of Sciences, 2007).

Starobogatov, Y. I., Prozorova, L. A., Bogatov, V. V. & Saenko, E. M. Mollusks. in Key to freshwater invertebrates of Russia and adjacent lands. Vol. 6. Molluscs, polychaetes, nemerteans (Tsalolikhin, S. Y., ed.). 9–491 (Nauka, 2004).

Vinarski, M. V. & Kantor, Y. I. *Analytical catalogue of fresh and brackish water molluscs of Russia and adjacent countries* (A. N. Severtsov Institute of Ecology and Evolution of RAS, 2016).

SCIENTIFIC NAME

***Nodularia douglasiae* (Griffith & Pidgeon, 1833)**

TAXONOMIC NOTES

The taxonomic history of this species in Russia is complex, because multiple ecophenotypes were previously accepted as separate, valid species (Starobogatov et al. 2004; Vinarski and Kantor 2016). However, all these nominal taxa were considered synonyms of the widespread *Nodularia douglasiae* based on morphological and multi-locus molecular data (Graf 2007; Klishko et al. 2018; this study).

RED LIST ASSESSMENT

Least Concern (LC).

JUSTIFICATION

The species has a wide distribution with an estimated EOO of more than 1,000,000 km^2. No major threats are known for the species, therefore, this species is here listed as Least Concern (LC) in Russia.

GEOGRAPHIC RANGE

This species is widespread throughout the Amur and Razdolnaya basins (Klishko et al. 2018) and coastal rivers of the Okhotsk Sea drainage basin (Bogatov 2000; Martynov and Chernyshov 1992; Klishko et al. 2018); one record is known from Langry River, northwestern Sakhalin (Bogatov 2001).

HABITAT AND ECOLOGY

The species is a habitat generalist being found in a variety of rivers, streams, channels, lakes, and ponds with different kinds of substrate. The maximum life span is 13 years (usually up to 8-10 years) (O. Klishko, pers. obs.). Larvae release occurs from mid-June to July (O. Klishko,

pers. obs.). *Rhodeus sericeus* (Pallas, 1776) was occasionally recorded as the host fish in Transbaikalia (O. Klishko, pers. obs.).

THREATS

There are no known major threats to this species in Russia.

CONSERVATION ACTIONS

No major conservation actions are required for this species in Russia.

RESEARCH NEEDS

The species needs research on its population trends, detailed distribution and phylogeographic structure to delineate the primary evolutionary significant units throughout the distribution range.

REFERENCES

Bogatov, V. V. The first *Middendorffinaia* (Bivalvia, Unionoidea) in Okhotsk Region. *Zoologicheskii Zhurnal* **79**, 862–862 (2000).

Bogatov, V. V. New data on Unioniformes of Sakhalin Island. *Byulleten Dalnevostochnogo Malakologicheskogo Obshchestva* **5**, 71–77 (2001).

Graf, D. L. Palearctic freshwater mussel (Mollusca: Bivalvia: Unionoida) diversity and the Comparatory Method as a species concept. *Proceedings of the Academy of Natural Sciences of Philadelphia* **156**, 71–89; DOI:10.1635/0097-3157(2007)156[71:PFMMBU]2.0.CO;2 (2007).

Klishko, O. K., Lopes-Lima, M., Froufe, E., Bogan, A. E. & Abakumova, V. Y. Unravelling the systematics of *Nodularia* (Bivalvia, Unionidae) species from eastern Russia. *Systematics and Biodiversity* **16**, 287–301; DOI:10.1080/14772000.2017.1383527 (2018).

Martynov, A.V. & Chernyshev, A.V. New and rare species of freshwater bivalves from the Soviet Far East. *Zoologicheskii Zhurnal* **71**, 18–23 (1992).

Starobogatov, Y. I., Prozorova, L. A., Bogatov, V. V. & Saenko, E. M. Mollusks. in Key to freshwater invertebrates of Russia and adjacent lands. Vol. 6. Molluscs, polychaetes, nemerteans (Tsalolikhin, S. Y., ed.). 9–491 (Nauka, 2004).

Vinarski, M. V. & Kantor, Y. I. *Analytical catalogue of fresh and brackish water molluscs of Russia and adjacent countries* (A. N. Severtsov Institute of Ecology and Evolution of RAS, 2016).

SCIENTIFIC NAME

***Unio crassus* Retzius, 1788**

TAXONOMIC NOTES

The taxonomic history of this species in Russia is complex, because multiple ecophenotypes were previously accepted as separate, valid species (Starobogatov et al. 2004; Bogatov and Kijashko 2016; Vinarski and Kantor 2016). However, all these nominal taxa were recently considered synonyms of the widespread *Unio crassus* based on morphological and multi-locus molecular data (Graf 2007; Lopes-Lima et al. 2017; Klishko et al. 2017; this study).

RED LIST ASSESSMENT

Least Concern (LC).

JUSTIFICATION

This widespread species has a wide distribution with an estimated EOO of more than 2,000,000 km^2. No major threats are known to be affecting the Russian range, therefore, the species is here listed as Least Concern (LC) in Russia.

GEOGRAPHIC RANGE

This species is widespread throughout European Russia (water bodies of Baltic, Black, Azov, and Caspian Sea drainage basins), western Urals, and Ural River drainage in Russia and Kazakhstan.

HABITAT AND ECOLOGY

It is found in slowly flowing river sections in clay and sandy substrates. While Zhadin (1938) noted that larvae release occurred in July in the Oka River, Volga Basin, there is no data about the fish hosts, or any other aspects of its life cycle in Russia.

THREATS

There are no known major threats to this species in Russia.

CONSERVATION ACTIONS

No major conservation actions are required for this species in Russia.

RESEARCH NEEDS

The species needs research on its population trends, detailed distribution and basic life-history traits such as fish hosts use, age of maturity, life span, and growth, as well as on its

phylogeographic structure to delineate the primary evolutionary significant units throughout Russia.

REFERENCES

Bogatov, V. V. & Kijashko, P. V. Class of bivalve mollusks – Bivalvia Linnaeus, 1758. in *Key of freshwater zooplankton and zoobenthos of the fresh water in European Russia. Vol. 2* (Alekseev, V. R. & Tsalolikhin, S. J., eds.) 285–334 (KMK Scientific Press Ltd., 2016).

Graf, D. L. Palearctic freshwater mussel (Mollusca: Bivalvia: Unionoida) diversity and the Comparatory Method as a species concept. *Proceedings of the Academy of Natural Sciences of Philadelphia* **156**, 71–89; DOI:10.1635/0097-3157(2007)156[71:PFMMBU]2.0.CO;2 (2007).

Klishko, O. et al. Taxonomic reassessment of the freshwater mussel genus *Unio* (Bivalvia: Unionidae) in Russia and Ukraine based on morphological and molecular data. *Zootaxa* **4286**, 93–112; DOI:10.11646/zootaxa.4286.1.4 (2017).

Lopes-Lima, M. et al. Conservation status of freshwater mussels in Europe: state of the art and future challenges. *Biological Reviews* **92**, 572–607; DOI:10.1111/brv.12244 (2017).

Starobogatov, Y. I., Prozorova, L. A., Bogatov, V. V. & Saenko, E. M. Mollusks. in Key to freshwater invertebrates of Russia and adjacent lands. Vol. 6. Molluscs, polychaetes, nemerteans (Tsalolikhin, S. Y., ed.). 9–491 (Nauka, 2004).

Vinarski, M. V. & Kantor, Y. I. *Analytical catalogue of fresh and brackish water molluscs of Russia and adjacent countries* (A. N. Severtsov Institute of Ecology and Evolution of RAS, 2016).

Zhadin, V. I. Fam. Unionidae. *Faune de l'URSS, Mollusques* **4**, 1–170 (1938).

SCIENTIFIC NAME

Unio pictorum **(Linnaeus, 1758)**

TAXONOMIC NOTES

The taxonomic history of this species in Russia is complex, because multiple ecophenotypes were previously accepted as separate, valid species (Starobogatov et al. 2004; Bogatov and Kijashko 2016; Vinarski and Kantor 2016). However, all these nominal taxa from European Russia were considered synonyms of the widespread *Unio pictorum* based on morphological

and multi-locus molecular data (Graf 2007; Lopes-Lima et al. 2017; Klishko et al. 2017; this study).

RED LIST ASSESSMENT

Least Concern (LC).

JUSTIFICATION

This species has wide distribution with an estimated EOO more than 3,000,000 km^2 and no major threats are known in Russia, therefore, the species is here listed as Least Concern (LC) in Russia.

GEOGRAPHIC RANGE

This species is widespread throughout European Russia and western Urals; introduced in Lake Kenon, Amur Basin, Transbaikalia (Klishko et al. 2017).

HABITAT AND ECOLOGY

Generalist species, which is found in a variety of lakes, channels, and slowly flowing river sections with clay and sandy substrates. The age of maturity is 2-3 years. Larvae release occurs from Late May to August in the Volga Delta (Antonova 1991) and in the Kenon Lake in Transbaikalia (Klishko 2012), while Zhadin (1938) noted that the larvae release occurred in July in the Oka River, Volga Basin. The host fishes are *Perca fluviatilis* Linnaeus, 1758, *Gymnocephalus cernua* (Linnaeus, 1758), *Rutilus* spp., *Leuciscus idus* (Linnaeus, 1758), *Pelecus cultratus* (Linnaeus, 1758), *Alburnus alburnus* (Linnaeus, 1758), and others (Zhadin 1938). The host fishes of an introduced population in the Kenon Lake are *Perca fluviatilis* Linnaeus, 1758 and *Rhodeus* spp. (Klishko 2012; O. Klishko, pers. obs.).

THREATS

There are no known major threats to this species in Russia.

CONSERVATION ACTIONS

No major conservation actions are required for this species in Russia.

RESEARCH NEEDS

The species needs research on its population trends, detailed distribution and phylogeographic structure to delineate the primary evolutionary significant units throughout Russia.

REFERENCES

Antonova, A.A. Connection of reproductive cycles of the Unionidae of the Volga Delta with environmental factors. *Proceedings of the Zoological Institute, Academy of Sciences of the USSR* **228**, 12–29 (1991).

Bogatov, V. V. & Kijashko, P. V. Class of bivalve mollusks – Bivalvia Linnaeus, 1758. in *Key of freshwater zooplankton and zoobenthos of the fresh water in European Russia. Vol. 2* (Alekseev, V. R. & Tsalolikhin, S. J., eds.) 285–334 (KMK Scientific Press Ltd., 2016).

Graf, D. L. Palearctic freshwater mussel (Mollusca: Bivalvia: Unionoida) diversity and the Comparatory Method as a species concept. *Proceedings of the Academy of Natural Sciences of Philadelphia* **156**, 71–89; DOI:10.1635/0097-3157(2007)156[71:PFMMBU]2.0.CO;2 (2007).

Klishko, O.K. Some data on reproductive biology of the freshwater mussels (Margaritiferidae, Unionidae) and their relationships with bitterlings (Cyprinidae) in Transbaikalye. *The Bulletin of the Russian Far East Malacological Society* **15/16**, 31–55 (2012).

Klishko, O. et al. Taxonomic reassessment of the freshwater mussel genus *Unio* (Bivalvia: Unionidae) in Russia and Ukraine based on morphological and molecular data. *Zootaxa* **4286**, 93–112; DOI:10.11646/zootaxa.4286.1.4 (2017).

Lopes-Lima, M. et al. Conservation status of freshwater mussels in Europe: state of the art and future challenges. *Biological Reviews* **92**, 572–607; DOI:10.1111/brv.12244 (2017).

Starobogatov, Y. I., Prozorova, L. A., Bogatov, V. V. & Saenko, E. M. Mollusks. in Key to freshwater invertebrates of Russia and adjacent lands. Vol. 6. Molluscs, polychaetes, nemerteans (Tsalolikhin, S. Y., ed.). 9–491 (Nauka, 2004).

Vinarski, M. V. & Kantor, Y. I. *Analytical catalogue of fresh and brackish water molluscs of Russia and adjacent countries* (A. N. Severtsov Institute of Ecology and Evolution of RAS, 2016).

Zhadin, V. I. Fam. Unionidae. *Faune de l'URSS, Mollusques* **4**, 1–170 (1938).

SCIENTIFIC NAME

***Unio tumidus* Retzius, 1788**

TAXONOMIC NOTES

The taxonomic history of this species in Russia is complex, because multiple ecophenotypes were previously accepted as separate, valid species (Starobogatov et al. 2004; Bogatov and Kijashko 2016; Vinarski and Kantor 2016). However, all these nominal taxa were considered synonyms of the widespread *Unio tumidus* based on morphological and multi-locus molecular data (Graf 2007; Lopes-Lima et al. 2017; Klishko et al. 2017; this study).

RED LIST ASSESSMENT

Least Concern (LC).

JUSTIFICATION

This species has a wide distribution with an estimated EOO of more than 3,000,000 km^2 and no major threats, therefore, the species is here listed as Least Concern (LC) in Russia.

GEOGRAPHIC RANGE

This species is widespread throughout European Russia and western Urals, including the Ural River. There are few records from the Irtysh Basin in Western Siberia (Andreeva et al. 2009) and Kazakhstan, and historical records of freshly dead shells from the Ob' River in Western Siberia (Zhadin 1938). This species was occasionally introduced in Lake Kenon and the Upper Amur Basin in Transbaikalia (Klishko et al. 2017).

HABITAT AND ECOLOGY

Generalist species, which is found in a variety of lakes, channels, and slowly flowing river sections with clay and sandy substrates. Larvae release occurs from Late May to August in the Volga Delta (Antonova 1991) and in the Kenon Lake in Transbaikalia (Klishko 2012), while Zhadin (1938) noted that the larvae release occurred in July in the Oka River, Volga Basin. The host fishes are *Perca fluviatilis* Linnaeus, 1758, *Gymnocephalus cernua* (Linnaeus, 1758), *Rutilus* spp., *Leuciscus idus* (Linnaeus, 1758), *Pelecus cultratus* (Linnaeus, 1758), *Alburnus alburnus* (Linnaeus, 1758), and other widespread fish species (Zhadin 1938). The host fishes of an introduced population in the Upper Amur Basin are *Perca fluviatilis* Linnaeus, 1758, *Rhodeus* spp. (Klishko 2012; O. Klishko, pers. obs.), and *Brachymystax lenok* (Pallas, 1773) (this study).

THREATS

There are no known threats to this species in Russia.

CONSERVATION ACTIONS

No major conservation actions are required for this species in Russia.

RESEARCH NEEDS

The species needs research on its population trends, detailed distribution and phylogeographic structure to delineate the primary evolutionary significant units throughout Russia.

REFERENCES

Andreeva, S. I., Vinarski, M. V. & Karimov, A. V. The first record of *Unio* species (Bivalvia: Unionidae) in the Irtysh River basin (Western Siberia, Russia). *Mollusca* **27**, 87–91 (2009).

Antonova, A.A. Connection of reproductive cycles of the Unionidae of the Volga Delta with environmental factors. *Proceedings of the Zoological Institute, Academy of Sciences of the USSR* **228**, 12–29 (1991).

Bogatov, V. V. & Kijashko, P. V. Class of bivalve mollusks – Bivalvia Linnaeus, 1758. in *Key of freshwater zooplankton and zoobenthos of the fresh water in European Russia. Vol. 2* (Alekseev, V. R. & Tsalolikhin, S. J., eds.) 285–334 (KMK Scientific Press Ltd., 2016).

Graf, D. L. Palearctic freshwater mussel (Mollusca: Bivalvia: Unionoida) diversity and the Comparatory Method as a species concept. *Proceedings of the Academy of Natural Sciences of Philadelphia* **156**, 71–89; DOI:10.1635/0097-3157(2007)156[71:PFMMBU]2.0.CO;2 (2007).

Klishko, O.K. Some data on reproductive biology of the freshwater mussels (Margaritiferidae, Unionidae) and their relationships with bitterlings (Cyprinidae) in Transbaikalye. *The Bulletin of the Russian Far East Malacological Society* **15/16**, 31–55 (2012).

Klishko, O. et al. Taxonomic reassessment of the freshwater mussel genus *Unio* (Bivalvia: Unionidae) in Russia and Ukraine based on morphological and molecular data. *Zootaxa* **4286**, 93–112; DOI:10.11646/zootaxa.4286.1.4 (2017).

Lopes-Lima, M. et al. Conservation status of freshwater mussels in Europe: state of the art and future challenges. *Biological Reviews* **92**, 572–607; DOI:10.1111/brv.12244 (2017).

Starobogatov, Y. I., Prozorova, L. A., Bogatov, V. V. & Saenko, E. M. Mollusks. in Key to freshwater invertebrates of Russia and adjacent lands. Vol. 6. Molluscs, polychaetes, nemerteans (Tsalolikhin, S. Y., ed.). 9–491 (Nauka, 2004).

Vinarski, M. V. & Kantor, Y. I. *Analytical catalogue of fresh and brackish water molluscs of Russia and adjacent countries* (A. N. Severtsov Institute of Ecology and Evolution of RAS, 2016).

Zhadin, V. I. Fam. Unionidae. *Faune de l'URSS, Mollusques* **4**, 1–170 (1938).
